# Temporal Integrative Analysis of mRNA and microRNAs Expression Profiles and Epigenetic Alterations in Female SAMP8, a Model of Age-Related Cognitive Decline

**DOI:** 10.3389/fgene.2018.00596

**Published:** 2018-12-11

**Authors:** Marta Cosín-Tomás, María Jesús Álvarez-López, Júlia Companys-Alemany, Perla Kaliman, Celia González-Castillo, Daniel Ortuño-Sahagún, Mercè Pallàs, Christian Griñán-Ferré

**Affiliations:** ^1^Department of Pharmacology and Therapeutic Chemistry, Institute of Neuroscience, University of Barcelona, Barcelona, Spain; ^2^Departments of Human Genetics and Pediatrics, Research Institute of the McGill University Health Centre, Montreal, QC, Canada; ^3^Faculty of Health Sciences, Universitat Oberta de Catalunya, Barcelona, Spain; ^4^Tecnologico de Monterrey, Escuela de Medicina y Ciencias de la Salud, Zapopan, Mexico; ^5^Centro Universitario de Ciencias de la Salud, Instituto de Investigación en Ciencias Biomédicas, Universidad de Guadalajara, Guadalajara, Mexico

**Keywords:** epigenetics, aging, brain, Alzheimer's disease, chromatin-modifying enzymes, microRNA, DNA methylation, histone acetylation

## Abstract

A growing body of research shows that epigenetic mechanisms are critically involved in normal and pathological aging. The Senescence-Accelerated Mouse Prone 8 (SAMP8) can be considered a useful tool to better understand the dynamics of the global epigenetic landscape during the aging process since its phenotype is not fully explained by genetic factors. Here we investigated dysfunctional age-related transcriptional profiles and epigenetic programming enzymes in the hippocampus of 2- and 9-month-old SAMP8 female mice using the Senescent-Accelerated Resistant 1 (SAMR1) mouse strain as control. SAMP8 mice presented 1,062 genes dysregulated at 2 months of age, and 1,033 genes at 9 months, with 92 genes concurrently dysregulated at both ages compared to age-matched SAMR1. SAMP8 mice showed a significant decrease in global DNA methylation (5-mC) at 2 months while hydroxymethylation (5-hmC) levels were increased in SAMP8 mice at 2 and 9 months of age compared to SAMR1. These changes were accompanied by changes in the expression of several enzymes that regulate 5-mC and methylcytosine oxidation. Acetylated H3 and H4 histone levels were significantly diminished in SAMP8 mice at 2-month-old compared to SAMR1 and altered Histone DeACetylase (HDACs) profiles were detected in both young and old SAMP8 mice. We analyzed 84 different mouse miRNAs known to be altered in neurological diseases or involved in neuronal development. Compared with SAMR1, SAMP8 mice showed 28 and 17 miRNAs differentially expressed at 2 and 9 months of age, respectively; 6 of these miRNAs overlapped at both ages. We used several bioinformatic approaches to integrate our data in mRNA:miRNA regulatory networks and functional predictions for young and aged animals. In sum, our study reveals interplay between epigenetic mechanisms and gene networks that seems to be relevant for the progression toward a pathological aging and provides several potential markers and therapeutic candidates for Alzheimer's Disease (AD) and age-related cognitive impairment.

## Introduction

With the dramatic rise in life expectancy, the prevention or delay of age-related cognitive decline is becoming a critical issue to improve human health-span (Prince et al., 2013, 2015). Aging is a multifaceted process in the continuous, progressive, and inevitable cycle of life; it is responsible for various depletive alterations in the physiological function of organisms and is molecularly associated with an increase in transcriptional noise (López-Otín et al., 2013). Research on age-related disorders has recently focused in epigenetic mechanisms since they are known to regulate gene expression in a stable and potentially reversible way and allow for the integration of long-lasting non-genetic inputs in the genome. Thus, a growing number of investigations have revealed that epigenetic mechanisms can modify the gene expression of processes involved in the etiopathological processes of Alzheimer's Disease (AD): Oxidative Stress (OS); mitochondrial and immune function; neuroinflammation and neurotoxicity (Wang et al., 2008; Gräff et al., 2012; Hendrickx et al., 2014; Iwata et al., 2014; Gjoneska et al., 2015; Yu et al., 2015; Grossi et al., 2016; Smith et al., 2016).

Aging is the main risk factor for AD which is also influenced by genetic factors such as the presence of full penetrant mutations in *Amyloid precursor protein (APP), Presenilin 1* and *2 (PSEN1* and *PSEN2)* and *Apolipoprotein E (APOE)*, sex (prevalence and incidence of AD are higher in women) and environmental and lifestyle factors (Jiang et al., 2013; Benayoun et al., 2015; Sohn et al., 2018). Converging evidence suggests that the pathophysiological process of AD starts decades before the first clinical symptoms. Although the etiology of the disease remains unclear, studies in both humans and rodents have shown that gene expression dysregulation is present in the development and progression of the emerging pathology (Heerboth et al., 2014; Mufson et al., 2016; Block and El-Osta, 2017).

One of the most characterized ways of epigenetic regulation is through the chromatin remodeling by DNA methylation or hydroxymethylation of the fifth position of cytosine (5-mC and 5-hmC, respectively) as well as by acetylation of histone residues. These mechanisms play a pivotal role in several brain functions, as well as in cell senescence (Sidler et al., 2017) and highly influence aging, neuropsychiatric disorders and neurodegenerative diseases such as AD (Irier and Jin, 2012; Coppieters et al., 2014; Millan, 2014; Spiegel et al., 2014; Wen et al., 2016; Ponnaluri et al., 2017). 5-hmC is the hydroxymethylated form of 5-mC, which is unusually abundant in the adult brain (Coppieters et al., 2014). It is well-known that 5-mC is highly present in CpG islands (CGIs) and is principally associated with transcriptional silencing of genes (Jones, 2012). In contrast, 5-hmC and acetylation of histone lysine residues correlate with gene activation (Feng et al., 2007; Sun et al., 2014). Controversy exists about the magnitude of global 5-mC, 5-hmC and acetylation changes reported in the AD brain most probably because different brain regions have been analyzed and different detection techniques employed (Lashley et al., 2015; Sanchez-Mut and Gräff, 2015).

MicroRNAs (miRNAs), a class of small non-coding RNAs, are also involved in gene expression regulation. These molecules promote the post-transcriptional silencing of their target mRNAs, and alterations in miRNA profiles have been associated with aging (Jung and Suh, 2012) and cognitive and neurodegenerative disorders (Hébert et al., 2009; Goodall et al., 2013; Nadim et al., 2017). Particularly, abnormal levels of specific miRNAs have been detected in several regions of the AD brain (Femminella et al., 2015).

The Senescence-Accelerated Prone 8 (SAMP8) mouse strain is a mouse model of accelerated aging, phenotypically selected from the AKR / J strain by Dr. Takeda lab at Kyoto University (Miyamoto, 1997). These mice prematurely exhibit traits of an aging process in several organs and systems, including the brain, and show significant shortening of life expectancy (Miyamoto, 1997; Nomura and Okuma, 1999). Behavioral abnormalities, cognitive decline as well as characteristic features of AD are detectable in old mice (e.g., Aβ accumulation Tau hyperphosphorylation, neuronal loss, dendritic spine density and microgliosis), but also in young adults (e.g., astrogliosis and spongiform degeneration patterns) (Morley et al., 2012a,b). In general, SAMP8 mice are considered an excellent model to study brain aging and neurodegeneration (Pallàs, 2012), while the Senescent-Accelerated Resistant 1 (SAMR1) mice, with a similar genetic background to SAMP8 mice and normal aging characteristics, have been extensively used as an appropriate control model. The SAMP8 mouse model can be considered a useful tool to better understand the dynamics of the global epigenetic landscape during the aging process, since its phenotype is not fully explained by genetic factors (Griñán-Ferré et al., 2018).

The present work hypothesizes that early and late-onset transcriptional alterations triggered by a dysfunctional age-related epigenetic programming contribute, at least in part, to the accelerated senescence phenotype and cognitive decline observed in the SAMP8 mouse model. Given the central role of the hippocampus in memory and learning (Leuner and Gould, 2010; Jahn, 2013), and the fact that this tissue is particularly impaired in AD patients, we explored changes in the expression of 22,000 genes, and measured levels of epigenetic marks (5-mC/5-hmC, histone acetylation), miRNAs and chromatin-modifying enzymes in the hippocampus of 2- and 9 month-old SAMP8 and SAMR1 mice. We chose to conduct the experiments in female mice since women have a higher risk of developing AD and females are underrepresented in animal models of the disease (Beery and Zucker, 2010; Lin and Doraiswamy, 2015; Nebel et al., 2018; Sohn et al., 2018).

## Methods

### Mouse Handling

Female SAMR1 (*n* = 40) and female SAMP8 mice (*n* = 40) (Envigo), of 2 and 9 months of age were used for the present experiments. Animals had free access to food and water and were maintained under standard temperature conditions (22 ± 2°C) and 12:12 h light-dark cycles (300 lx/0 lx).

Studies were performed following the Institutional Guidelines for the Care and Use of Laboratory Animals established by the Animal Experimentation Ethics Committee (CEEA) at the University of Barcelona.

### Brain Processing and Subcellular Fractionation

Animals were euthanized by cervical dislocation. Subsequently, brains were immediately removed and, the hippocampus was then isolated, frozen on powdered dry ice, and maintained at −80°C until protein extraction, RNA and, DNA isolation.

For subcellular fractionation, 150 μL of lysis buffer (10 mM HEPES pH 7.9, 10 mM KCl, 0.1 mM EDTA pH 8, 0.1 mM EGTA pH 8, 1 mM DTT, 1 mM PMSF, protease inhibitors) was added to each sample and incubated on ice for 15 min. Samples were then homogenized with a tissue homogenizer, and 12.5 μL of Igepal 1% was added to each Eppendorf before vortexing for 15 s. Following 30 s of full-speed centrifugation at 4°C, supernatants were collected (cytoplasmic fraction); 80 μL of lysis buffer (with 20 mM HEPES pH 7.9, 0.4 M NaCl, 1 mM EDTA pH 8, 0.1 mM EGTA pH 8, 20% Glycerol 1 mM DTT, 1 mM PMSF, and protease inhibitors) was added to each pellet and incubated under agitation at 4°C for 15 min. Subsequently, the samples were centrifuged for 10 min at full speed at 4°C. Supernatants were collected (nuclear fraction) and 40 μL of lysis buffer + HCl (lysis buffer with 0.2 N HCl) was added to the pellet. After a 30-min incubation on ice, samples were centrifuged, again at full speed, at 4°C for 10 min, and the supernatants were collected (histone fraction).

### Western Blotting

For Western blot (Wb), 15 μg of nuclear and cytoplasmic hippocampal fractions and 5 μg of histone fraction were used. Protein samples from 16 females (*n* = 4 per group) were separated by Sodium Dodecyl Sulfate-PolyAcrylamide Gel Electrolysis (SDS–PAGE) (8–18%) and transferred into PVDF membranes (Millipore). The membranes were blocked in 5% non-fat milk in TBS containing 0.1% Tween 20 (TBS-T) for 1 h at room temperature, followed by an overnight incubation at 4°C with the primary antibodies listed in Supplementary Material 1. Membranes were then washed and incubated with secondary antibodies for 1 h at room temperature. Immunoreactive protein was viewed with a chemiluminescence-based detection kit, following the manufacturer's protocol (ECL Kit; Millipore), and digital images were acquired using a ChemiDoc XRS+ System (BioRad). Semi-quantitative analyses were carried out using Image Lab software (BioRad), and results were expressed in Arbitrary Units (AU). Protein loading was routinely monitored by phenol red staining of the membrane or by immunodetection of TBP and GADPH.

### Global DNA Methylation and Hydroxymethylation Determination

Isolation of genomic DNA from 16 samples (*n* = 4 per group) was conducted using the FitAmp^TM^ Blood and Cultured Cell DNA Extraction Kit according to the manufacturer's instructions. Then, Methylflash Methylated DNA Quantification Kit (Epigentek, Farmingdale, NY, United States) and MethylFlash HydroxyMethylated DNA Quantification Kit were used in order to detect methylated and hydroxymethylated DNA. Briefly, these kits are based on specific antibody detection of 5-mC and 5-hmC residues, which trigger an ELISA-like reaction that allows colorimetric quantification by reading absorbance at 450 nm using a Microplate Photometer. The absolute amount of methylated or hydroxymethylated DNA (proportional to the Optical Density [OD] intensity) was measured and quantified using a standard curve plotting OD values vs. five serial dilutions of a control methylated and hydroxymethylated DNA (0.5–10 ng).

### RNA Extraction and MicroRNAs Expression Array

For microRNAs expression array, total RNA was extracted employing the *mir*Vana™ RNA Isolation Kit (Applied Biosystems) according to the manufacturer's instructions. The yield, purity, and quality of the samples were determined by the A260/280 ratio in a NanoDrop® ND-1000 apparatus (Thermo Scientific).

RNA samples from 16 females (*n* = 4 per group) were converted into cDNA through a Reverse Transcription (RT) reaction using the miScript II RT Kit (Qiagen) according to the manufacturer's instructions. The expression of 84 mature miRNAs was then analyzed using miScript® miRNA PCR Array-Neurological Development & Disease miRNA PCR Array (Qiagen). miRNAs expression was measured in a CFX384 Touch™ Real-Time PCR Detection System (BioRad through SYBR® Green-based real-time PCR). The data obtained were processed using the Web-based miScript miRNAs PCR Array online software data-analysis tool. The mean of the relative gene expression of the small non-coding RNAs (sncRNA) *SNORD68, SNORD72, SNORD95*, and *SNORD96A* was used to normalize results since they presented similar expression levels between groups and the lowest Standard Deviations (SD) among all of the housekeepings proposed.

### Microarray Printing, Probe Preparation, Hybridization, Data Acquisition, and Analysis

Complementary DNA (cDNA) was synthesized from 10 μg of total RNA isolated from whole hippocampus (*n* = 3 per group), incorporating dUTP-Cy3 or dUTP-Cy5. Equal quantities of the labeled cDNA were hybridized to the *Mus musculus* 22 thousand 65-mer Oligo Library from Sigma-Genosys, as described previously (Rojas-Mayorquín et al., 2008; Ortuño-Sahagún et al., 2012). Acquisition and quantification of the microarray images were performed in a ScanArray 4,000 apparatus employing the accompanying ScanArray 4,000 software (Packard BioChips; Perkin-Elmer, MN, United States). All of the data and normalized microarray generated in this study were deposited in the NCBI Gene Expression Omnibus (GEO). All images were captured as described (Rojas-Mayorquín et al., 2008). In all cases, the fluorescence signal was from seven to 10 times more intensive than the background signal, and the background evaluation was always made just beside the labeled spot.

Microarray data analysis was performed using the GenArise free software, developed by the Computing Unit at the Institute of Cellular Physiology of the National Autonomous University of Mexico (UNAM) (Gómez-Mayen et al., 2006) to identify genes that are good candidates for differential expression by calculating an intensity-dependent score. GeneArise performs a number of transformations: background correction, lowest normalization, intensity filter, replicate analysis, and selection of differentially expressed genes. According to these criteria, elements with a Z-score of more than 2 standard deviations are genes likely to be differentially expressed (Gómez-Mayen et al., 2006; Ortuño-Sahagún et al., 2012).

### RNA Extraction and Gene Expression Determination

Total RNA isolation was carried out using TRIzol® reagent according to manufacturer's instructions. The yield, purity, and quality of RNA were determined spectrophotometrically with a NanoDrop™ ND-1000 (Thermo Scientific) apparatus and an Agilent 2100B Bioanalyzer (Agilent Technologies). RNAs with 260/280 ratios and RIN higher than 1.9 and 7.5, respectively, were selected. Reverse Transcription-Polymerase Chain Reaction (RT-PCR) was performed as follows: 2 μg of messenger RNA (mRNA) was reverse-transcribed using the High Capacity cDNA Reverse Transcription Kit (Applied Biosystems). Real-time quantitative PCR (qPCR) was used to quantify mRNA expression of chromatin-modifying genes as well as to validate selected genes from microarray data results.

SYBR® Green real-time PCR was performed in a Step One Plus Detection System (Applied-Biosystems) employing SYBR® Green PCR Master Mix (Applied-Biosystems). Each reaction mixture contained 7.5 μL of complementary DNA (cDNA) (which concentration was 2 μg), 0.75 μL of each primer (which concentration was 100 nM), and 7.5 μL of SYBR® Green PCR Master Mix (2X).

TaqMan-based real-time PCR (Applied Biosystems) was also performed in a Step One Plus Detection System (Applied-Biosystems). Each 20 μL of TaqMan reaction contained 9 μL of cDNA (25 ng), 1 μL 20X probe of TaqMan Gene Expression Assays and 10 μL of 2X TaqMan Universal PCR Master Mix.

Data were analyzed utilizing the comparative Cycle threshold (Ct) method (ΔΔCt), where the housekeeping gene level was used to normalize differences in sample loading and preparation (Griñán-Ferré et al., 2016). Normalization of expression levels was performed with β*-actin* for SYBR® Green-based real-time PCR results and TATA-binding protein *(Tbp)* for TaqMan-based real-time PCR. Primers and TaqMan probes are listed in Supplementary Material 2. Each sample (*n* = 4 per group) was analyzed in duplicate, and the results represent the n-fold difference of the transcript levels among different groups.

### MicroRNAs Validation by Single Real-Time PCR

TaqMan-based real-time PCR was performed on the detection system StepOnePlus (Applied Biosystems) for microRNA expression. In compliance with the TaqMan Small RNA Assays Protocol, each reaction mixture contained 1 μL of TaqMan Small RNA Assay (20X), 1.33 μL of product from RT reaction, 10 μL of Quantimix Easy Kit probes (BioTools) (instead of TaqMan Universal PCR Master Mix II [2X]), and 7.67 μL of nuclease-free water. Data were analyzed using the comparative cycle threshold (Ct) method (ΔΔCt), in which the U6 snRNA transcript level was employed to normalize differences in sample loading and preparation. Each sample (*n* = 4 per group) was analyzed in duplicate, and the results represent the n-fold difference of the transcript levels among different groups.

### Data Analysis

The statistical analysis was conducted using GraphPad Prism ver. Six statistical software. Data are expressed as the mean ± Standard Error of the Mean (SEM) of at least 3 samples per group. Time and group effects for mRNA expression profile and epigenetic marks were assessed by the Two-Way ANOVA analysis of variance, followed by Tukey *post-hoc* analysis. Comparisons between groups and mRNA:miRNA validation were also performed by two-tail Student's *T-*test for independent samples. Statistical significance was considered when *p* < 0.05. The Statistical outliers were determined with Grubbs' test and subsequently removed from the analysis.

### Prediction of mRNA:miRNA Pairs, Network Construction and Go Pathway Analysis

A miRNA target gene prediction tool from an R package, RmiR, was used to predict putative target genes of differentially expressed miRNAs (Favero, 2018). Putative mRNA:miRNA pairs were predicted by at least five of the following six commonly used miRNA target databases: Target Scan, miRanda, mirTarget, tarBase and, miRBase. The threshold for the target was predicted by ≥5 algorithms. miRNAs up or downregulate the expression of its target genes post-transcriptionally, so the target genes whose expressions were inversely correlated with corresponding miRNAs were selected as miRNA targets with high accuracy based on the obtained gene expression data. We assigned priority to the mRNA:miRNA pairs included in networks that are more likely to play critical roles in aging, neurodegeneration, and AD according to the bibliography. Using GeneMANIA, an App from Cytoscape (http://www.cytoscape.org/) (Kutmon et al., 2013), miRNA-target gene interaction networks were constructed (Santiago and Potashkin, 2014).

### Functional Annotation

To analyse the function and the potential pathways of miRNA-target genes, functional annotations by Gene Ontology (GO) enrichment analysis of molecular functions, biological processes, among others, were performed using Enrichr (Chen et al., 2013) and the interactive and collaborative HTML5 gene list enrichment analysis tool. Enrichr determines the distribution of the target gene list across GO terms and pathways. The *p*-value was calculated using the right-sided hypergeometric test and derived from the DataSet statistics tab of the Enrichr main page; GO categories with *p*-values and Benjamini adjustment < 0.05 were considered statistically significant.

## Results

### Hippocampal mRNAs Expression Profile Is Altered in Young and Aged Female SAMP8 Mice

**Figure 1:**
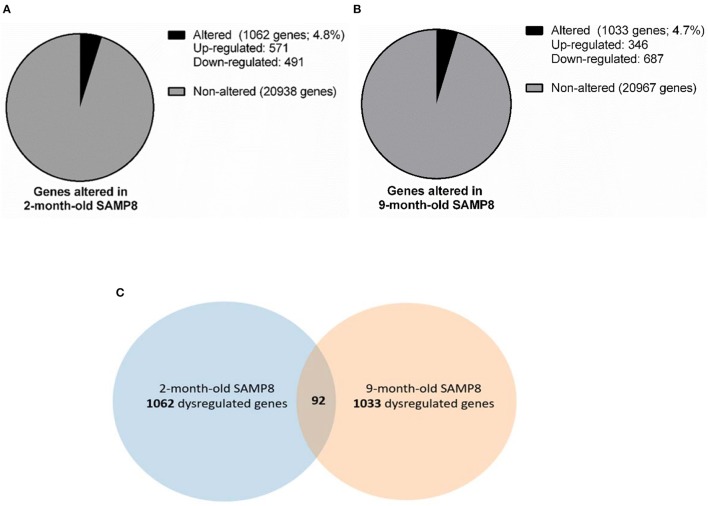
Gene dysregulation in 2- and 9-month-old SAMP8 mice compared to age-matched SAMR1. Representative pie chart of genes altered in the hippocampus of 2-month-old SAMP8 mice compared to age-matched SAMR1 **(A)**. Representative pie chart of genes altered in the hippocampus of 9-month-old SAMP8 mice compared to age-matched SAMR1 **(B)**. Venn diagram highlighting hippocampal genes concurrently altered in SAMP8 mice at 2 and 9 months of age **(C)**. Venn diagram includes genes significantly dysregulated in 2-month-old SAMP8 mice compared to age-matched SAMR1 mice (left circle), and genes significantly dysregulated in 9-month-old SAMP8 mice compared to age-matched SAMR1 mice (right circle). The intersection of circles corresponds to genes that are altered in SAMP8 at both early adulthood and aged stages.

With the aim of identifying early and late age-related genes involved in the brain (pathological) aging, we performed microarray studies in the hippocampus from 2 and 9 months of age SAMP8 and SAMR1 mice. From a total of 22,000 genes tested we selected those genes that yield a z-score >2.0 for further comparison analysis (i.e., genes whose expression in the SAMP8 mice is at least 2 standard deviations away from the reference group mean). Several genes were altered in SAMP8 mice compared to SAMR1 mice. At 2-month-old, 1,062 genes were dysregulated, 571 of which were upregulated and 491 downregulated (Figure 1A), while at 9 months, 1,033 genes were dysregulated, 346 of which were upregulated and 687 were downregulated (Figure 1B) (dysregulated genes are listed in Supplementary Material 3). Finally, according to microarray results, 20,938 genes were similarly expressed in both strains at 2 months, and 20,967 genes were similarly expressed in both strains at 9 months (Figures 1A,B).

Genes differentially expressed in SAMP8 and SAMR1 mice at 2 and 9 months of age were integrated into a Venn diagram. Remarkably, only 92 genes, approximately 5% of the total altered genes, were concurrently dysregulated in SAMP8 mice at both 2 and 9 months (Figure 1C, Venn diagram). The overlap between genes dysregulated at 2 and 9 months of age in SAMP8 mice was statistically significant, as indicated by Chi-square test with Yates correction (χ2 = 38.324, *p* < 0.001).

Additionally, to identify possible different expression profile between sexes, we performed a comparison of our results in mRNA expression profile with the results of the other two main studies of mRNA expression profile performed in male SAMP8 hippocampus at 2, 6 and 12 months of age from Cheng et al. (2007) and Cheng et al. (2013). Notably, analysis rendered that only 7 genes were concurrently dysregulated in both sexes, while 22 genes were concurrently dysregulated in the males SAMP8 studies. Results of dysregulated genes are listed and presented in a Venn diagram (Supplementary Material 6).

### Temporal Alterations in Hippocampal DNA Methylation and Hydroxymethylation Levels in Female SAMP8 Mice

**Figure 2:**
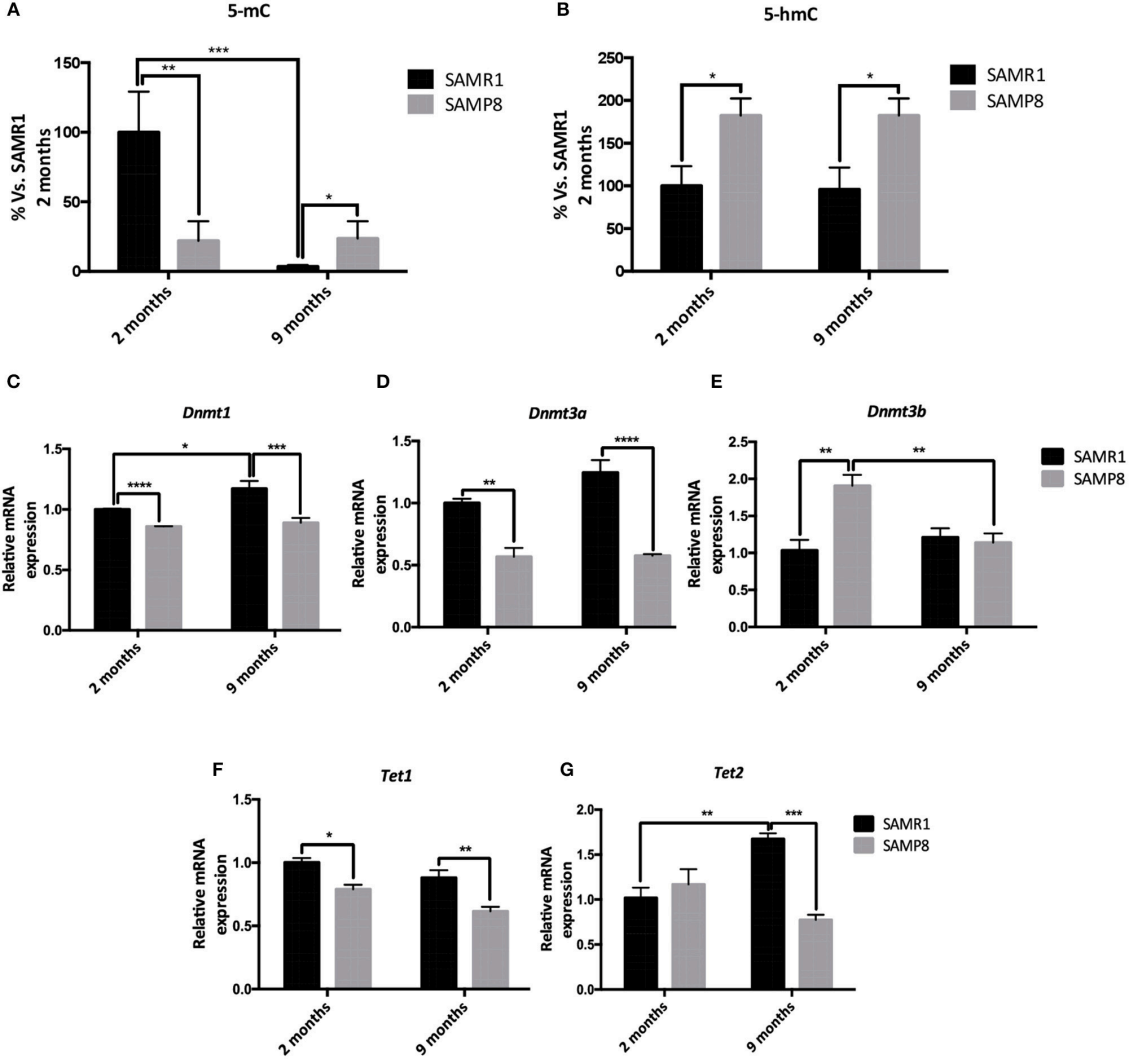
Global levels of 5-methylated cytosines and 5-hydroxymethylated cytosines in the hippocampus of 2 and 9 months of age SAMP8 and SAMR1 mice **(A,B)**. Relative gene expression of *Dnmt1, Dnmt3a, Dnmt3b, Tet1*, and *Tet2* in the hippocampus of 2- and 9-month-old SAMP8 and SAMR1 mice **(C,D,E,F,G)**. Gene expression was measured by real-time PCR analysis from hippocampal mRNA and expressed relative to *Tbp* or β*-actin* (*n* = 4–5/group). Mean ± standard error performed in triplicates are represented; Two-way ANOVA followed by Tukey's *post-hoc* test results are indicated as ^*^p < 0.05; ^**^p < 0.01; ^***^p < 0.001 and; ^****^p < 0.0001.

We first explored the potential contribution of 5-mC and 5-mhC on the temporal gene expression changes observed in SAMP8 mice. We determined global levels of 5-mC as well as 5-hmC in DNA samples from 2- and 9-month-old SAMP8 and SAMR1 mice. Compared to SAMR1, SAMP8 mice showed a significant decrease in global 5-mC at 2 months (Tukey's *post-hoc, p* < 0.01; Table 1 and Figure 2A). However, at 9 months SAMR1 mice presented a significant loss of this same epigenetic mark (Tukey's *post-hoc, p* < 0.001), while SAMP8 mice maintained the same levels as in 2 months (Tukey's *post-hoc, p* = ns; Figure 2A). Regarding 5-hmC levels, they were increased in SAMP8 mice at 2 months and remained increased at 9 months compared to SAMR1 mice [two-way ANOVA, *F*_(1, 8)_ = 14.32, *p* < 0.01, Table 1 and Figure 2B].

**Table 1:** Results of Two-way ANOVA analysis for epigenetic marks and epigenetic enzymes in the hippocampus of 2- and 9-month -old SAMP8 and SAMR1 mice.

		**Two-way ANOVA**					
		**Interaction**		**Age**		**Strain**	
**Epigenetic mark**	***N*****/group**	***F (DFn, DFd)***	***P-*****value**	***F (DFn, DFd)***	***P-*****value**	***F (DFn, DFd)***	***P-*****value**
5-mC	3	*F*_(1, 8)_ = 24.03	0.001	*F*_(1, 8)_ = 22.5	0.002	*F*_(1, 8)_ = 8.335	0.020
5-hmC	3	*F*_(1, 8)_ = 0.007	0.933	*F*_(1, 8)_ = 0.009	0.927	*F*_(1, 8)_ = 14.32	0.005
Ac-H3	4	*F*_(1, 12)_ = 6.375	0.027	*F*_(1, 12)_ = 15.2	0.002	*F*_(1, 12)_ = 3.163	0.101
Ac-H4	4	*F*_(1, 12)_ = 2.928e-005	0.996	*F*_(1, 12)_ = 7.807	0.016	*F*_(1, 12)_ = 2.274	0.157
**Epigenetic enzyme mRNA**							
*Dnmt1*	4	*F*_(1, 12)_ = 3.598	0.082	*F*_(1, 12)_ = 7.353	0.019	*F*_(1, 12)_ = 32.12	<0.001
*Dnmt3a*	4	*F*_(1, 12)_ = 3.454	0.088	*F*_(1, 12)_ = 4.001	0.069	*F*_(1, 12)_ = 74.96	<0.001
*Dnmt3b*	4	*F*_(1, 12)_ = 12.47	0.004	*F*_(1, 12)_ = 4.871	0.048	*F*_(1, 12)_ = 9.045	0.011
*Hdac1*	4	*F*_(1, 12)_ = 2.181	0.166	*F*_(1, 12)_ = 38.18	<0.001	*F*_(1, 12)_ = 10.32	0.007
*Hdac2*	4	*F*_(1, 12)_ = 17	0.001	*F*_(1, 12)_ = 14.23	0.003	*F*_(1, 12)_ = 22.92	<0.001
*Sirt1*	4	*F*_(1, 12)_ = 1.455	0.251	*F*_(1, 12)_ = 3.242	0.097	*F*_(1, 12)_ = 27.83	<0.001
*Sirt2*	4	*F*_(1, 12)_ = 1.982	0.184	*F*_(1, 12)_ = 1.208	0.293	*F*_(1, 12)_ = 4.512	0.055
*Sirt6*	4	*F*_(1, 12)_ = 7.19	0.020	*F*_(1, 12)_ = 19	<0.001	*F*_(1, 12)_ = 21.22	<0.001
*Tet1*	4	*F*_(1, 12)_ = 0.385	0.547	*F*_(1, 12)_ = 11.79	0.005	*F*_(1, 12)_ = 30.94	<0.001
*Tet2*	4	*F*_(1, 12)_ = 23.07	<0.001	*F*_(1, 12)_ = 1.401	0.260	*F*_(1, 12)_ = 11.78	0.005

5-mC is catalyzed by the specialized *de novo* DNA methyltransferase enzymes DNMT3A and DNMT3B, and perpetuated through cell divisions by DNMT1 (Ito et al., 2011); whereas Ten-Eleven Translocation Methylcytosine Dioxygenases (TETs) family enzymes are involved in the oxidation of 5-mC to 5-hmC as well as in the active DNA demethylation pathway (Lu et al., 2015). Therefore, we analyzed the gene expression of relevant DNMTs and TETs family members. *Dnmt1, Dnmt3a*, and *Tet1* genes were significantly downregulated in SAMP8 compared to SAMR1 mice at both 2 and 9 months of age [two-way ANOVA, *F*_(1, 12)_ = 32.12, *p* < 0.001; two-way ANOVA, *F*_(1, 12)_ = 74.96, *p* < 0.001; two-way ANOVA, *F*_(1, 12)_ = 9.045, *p* < 0.05, respectively; Table 1 and Figures 2C,D,F]. Interestingly, *Dnmt1* was significantly upregulated in the control group at 9 months compared to 2 months (Tukey's *post-hoc, p* < 0.05). *Dnmt3b* gene expression levels were increased in the senescent strain at 2 months (Tukey's *post-hoc, p* < 0.01); while at 9 months, it was downregulated, reaching similar levels than the control group (Tukey's *post-hoc, p* = ns; Table 1 and Figure 2E). Finally, *Tet2* gene expression was unaltered at 2 months of age, but was upregulated in SAMR1 mice at 9 months (Tukey's *post-hoc, p* < 0.01) reaching higher levels than age-matched SAMP8 (Tukey's *post-hoc, p* < 0.001; Table 1 and Figure 2G).

### Temporal Alterations in Hippocampal Histone Acetylation Levels in Female SAMP8 Mice

**Figure 3:**
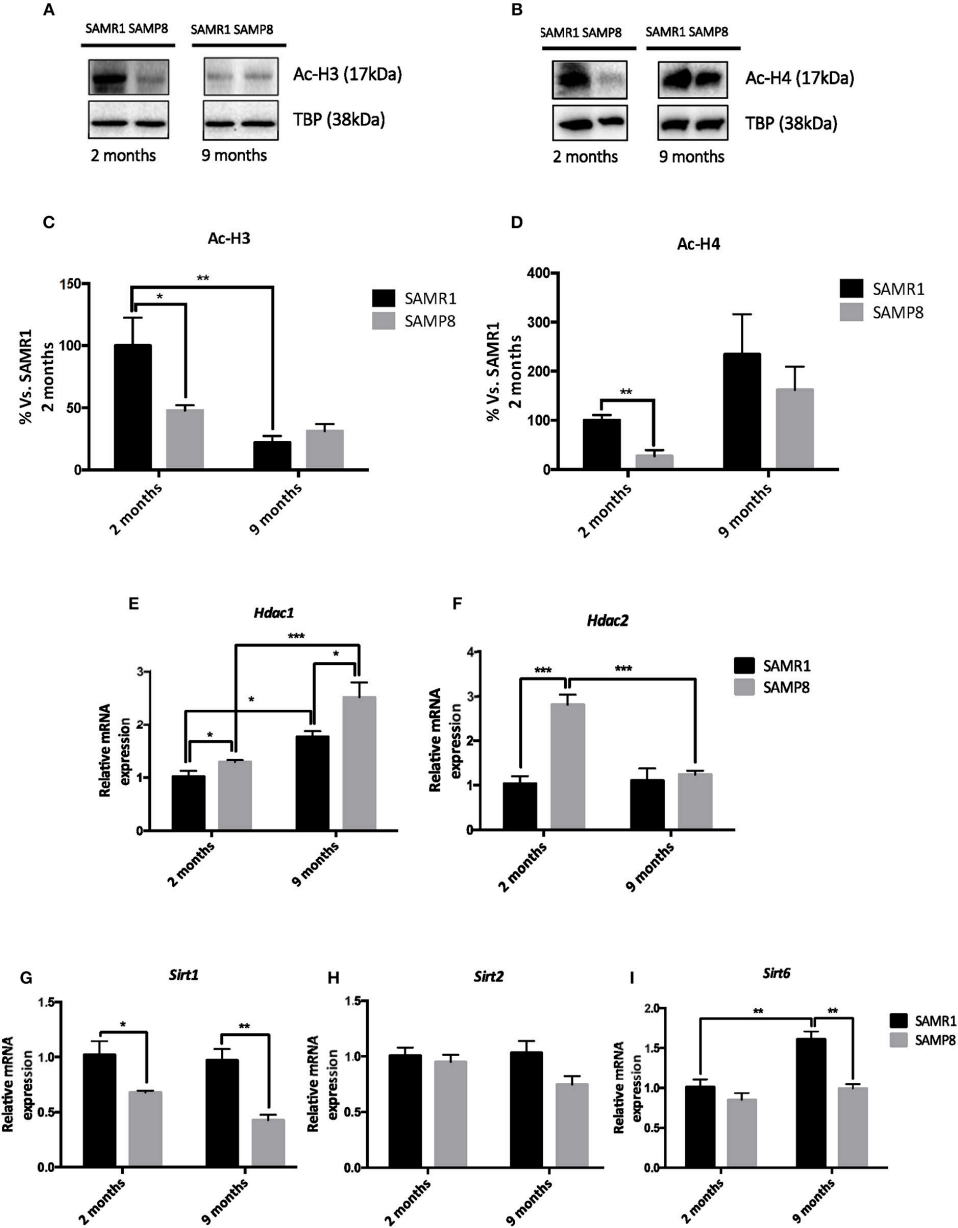
Representative Western blot (Wb) for acetylated and total H3 and H4 protein levels **(A,B)**, and quantification **(C,D)**. Relative gene expression of *Hdac1, Hdac2, Sirt1, Sirt2*, and *Sirt6* in the hippocampus of 2-and 9-month-old SAMP8 and SAMR1 mice **(E,F,G,H,I)**. Values in bar graphs are adjusted to 100% for protein levels of SAMR1 at different ages. Gene expression was measured by real-time PCR analysis from hippocampal mRNA and expressed relative to β*-actin* (*n* = 4–5/group). Mean ± standard error from five independent experiments performed in duplicates are represented; Two-way ANOVA followed by Tukey's *post-hoc* test results are indicated as ^*^p < 0.05; ^**^p < 0.01 and; ^***^p < 0.001.

We next explored whether changes in histone acetylation levels and its enzymatic machinery could be contributing to the temporal transcriptional dysregulation observed in SAMP8 mice. Acetylated H3 and H4 protein levels were significantly lower in SAMP8 mice at 2 months of age compared to SAMR1 mice, although in the case of H4 only by *T-*test (Tukey's *post-hoc, p* < 0.05; *T-*test, *p* < 0.05, respectively). Both groups of mice showed downregulation of acetylated H3 and upregulation of acetylated H4 marks at 9 months [two-way ANOVA, *F*_(1, 12)_ = 41.37, *p* < 0.01, two-way ANOVA, *F*_(1, 12)_ = 35.36, *p* < 0.05, respectively, Table 1], leading to similar levels between strains at this later stage (Tukey's *post-hoc, p* = ns; Figures 3A–D). In addition, we studied the gene expression of the main members of HDACs families I and III, which are known to be expressed in the hippocampus and to play a role in memory and learning, particularly in the context of AD (Kilgore et al., 2010; Volmar and Wahlestedt, 2015). The expression of both *Hdac1* and *Hdac2* (representative of HDACs class I family) genes was significantly increased in SAMP8 mice at 2 months compared to SAMR1 mice, although in the case of *Hdac1* only by *T-*test (*T-*test, *p* < 0.05; Tukey's *post-hoc, p* < 0.001, respectively). At 9 months, *Hdac1* was significantly upregulated in both groups [two-way ANOVA, *F*_(1, 12)_ = 38.18, *p* < 0.001, Table 1], but SAMP8 kept presenting higher gene expression levels of the enzyme (Tukey's *post-hoc, p* < 0.05); while for *Hdac2*, SAMP8 was downregulated at 9 months (Tukey's *post-hoc, p* < 0.001) reaching similar levels to the control group (Tukey's *post-hoc*, p = ns; Figures 3E,F). Regarding HDAC family III genes, we observed a significant reduction in *Sirt1* gene expression at 2 months in SAMP8 mice compared to age-matched SAMR1, which was maintained at 9 months [two-way ANOVA, *F*_(1, 12)_ = 27.83, *p* < 0.001, Table 1 and Figure 3G]. Similarly, *Sirt6* was significant downregulated in the senescent strain compared to SAMR1 mice, but only at 9 months (Tukey's *post-hoc, p* < 0.01; Figure 3I). We did not observe significant differences in gene expression of *Sirt2* among groups or ages (Table 1 and Figure 3H).

### Temporal Changes in Hippocampal miRNAs Expression Profile in Female SAMP8 Mice

**Figure 4:**
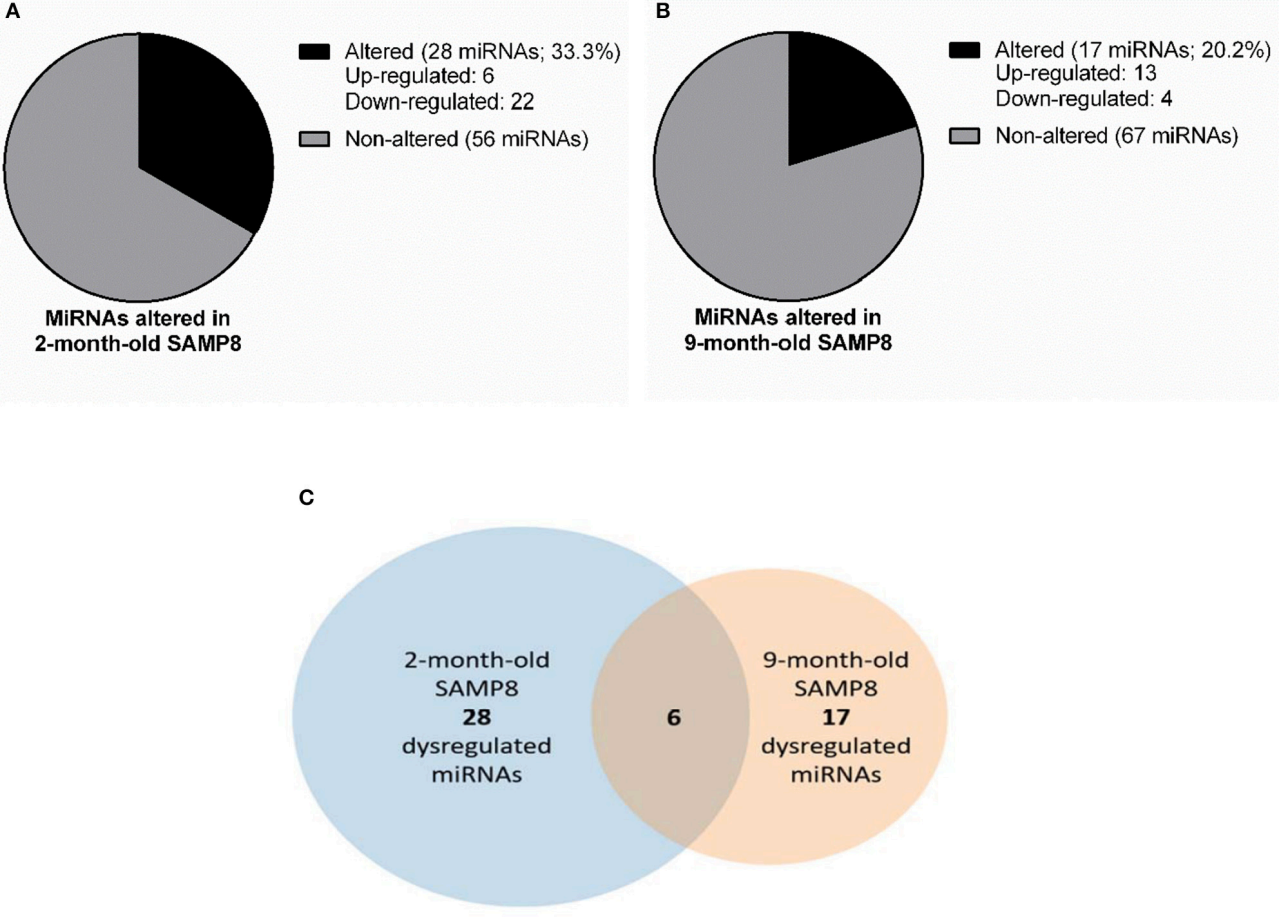
miRNAs dysregulation in SAMP8 mice compared to SAMR1 mice at 2 and 9 months of age. Representative pie chart of miRNAs altered in the hippocampus of 2-month-old SAMP8 mice compared to age-matched SAMR1 **(A)**. Representative pie chart of miRNAs altered in the hippocampus of 9-month-old SAMP8 mice compared to age-matched SAMR1 **(B)**. Venn diagram highlighting hippocampal miRNAs concurrently altered in SAMP8 mice at 2 and 9 months of age **(C)**. Venn diagram includes miRNAs significantly dysregulated in 2-month-old SAMP8 mice compared to age-matched SAMR1 (left circle), and miRNAs significantly dysregulated in 9-month-old SAMP8 mice compared to age-matched SAMR1 (right circle). The intersection of circles corresponds to miRNAs that are altered in SAMP8 mice at both early adulthood and aged stages.

To identify miRNAs that could be underlying the transcriptional changes observed in both young and aged SAMP8, we conducted a microRNA PCR array using hippocampal samples from 2 and 9 months of age SAMP8 and SAMR1 mice. This array analyzed 84 different mouse miRNAs known to be altered in neurological diseases or involved in neuronal development. We found that 39 miRNAs were altered in SAMP8 mice compared to SAMR1: 28 miRNAs were altered at 2 months of age, 6 of which were upregulated, and 22 downregulated (Figure 4A). At 9 months of age, 17 miRNAs were altered, 13 of which were upregulated, and 4 downregulated (Figure 4B). Altered miRNAs are listed in Supplementary Material 4.

MicroRNAs differentially expressed in SAMP8 and SAMR1 mice at 2 and 9 months were integrated into a Venn diagram. Only 6 miRNAs, approximately 15% of the total altered miRNAs, were found dysregulated in SAMP8 at both 2 and 9 months (Figure 4C Venn diagram). However, the overlap between miRNAs dysregulated in 2- and 9-month-old SAMP8 mice was not statistically significant, as indicated by Fisher's exact test (*p* > 0.1).

### Integrated mRNA:miRNA Regulatory Networks in Young and Aged Female SAMP8 Mice

**Figure 5:**
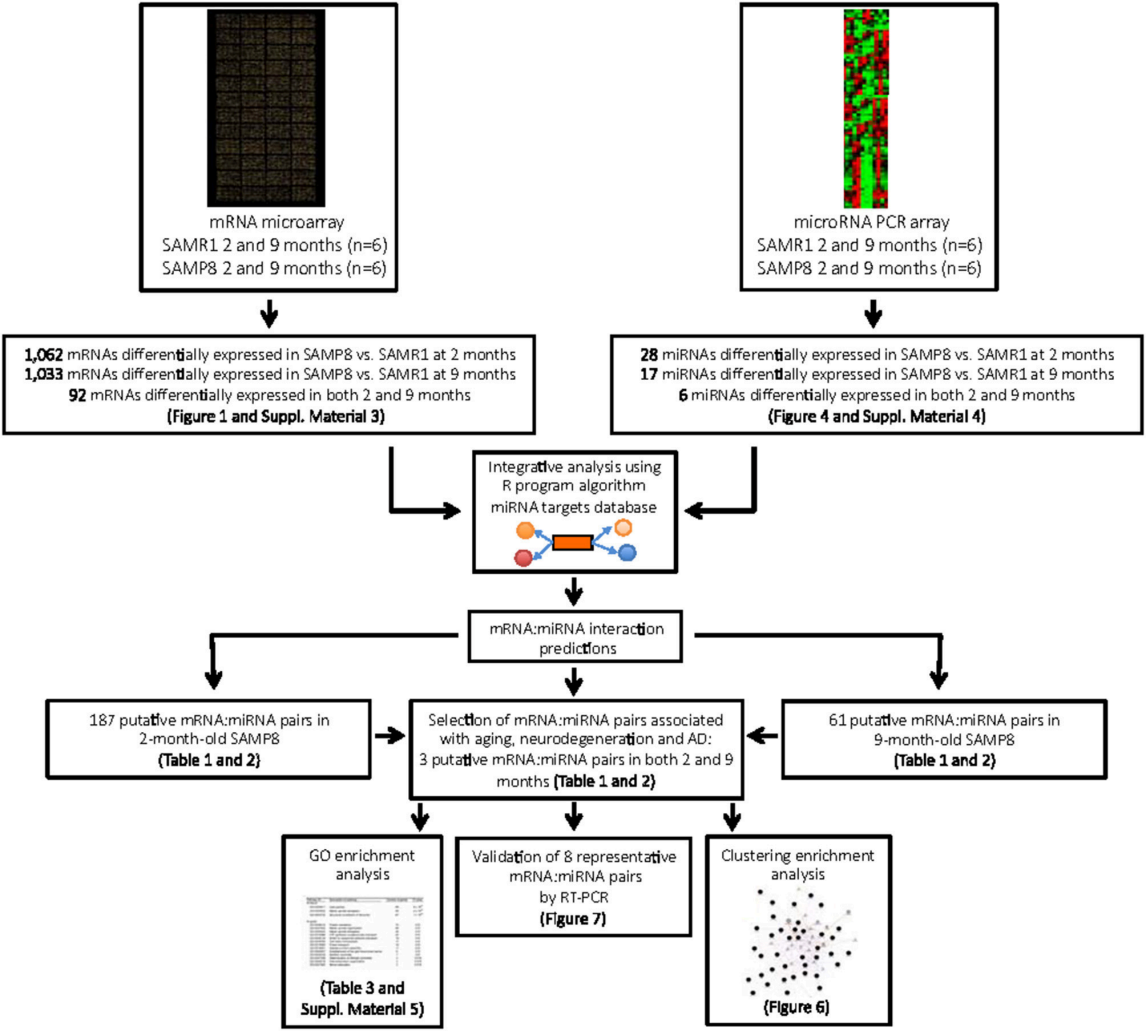
Systematic workflow used to explore mRNA:miRNA pairs differentially expressed in young and aged SAMP8 mice compared to age-matched SAMR1 , and their potential role in regulatory networks related with aging, neurodegeneration, and AD.

With the purpose of establishing possible regulatory relationships between significantly dysregulated genes and miRNAs in SAMP8 aging mice, we constructed co-expression networks at 2 and 9 months of age. We integrated six commonly used miRNA target databases to predict miRNA targets using a tool from an R package: RmiR (Yang et al., 2017). We calculated all the pair expression correlations from target relations using miRNAs target databases: Target Scan, miRanda, mirTarget, tarBase and, miRBase and selected putative mRNA:miRNA pairs by at least five of the six databases. Significant interactions of mRNA:miRNA were predicted by the co-expression network. We assigned priority to the mRNA:miRNA pairs included in networks that are more likely to play critical roles in aging, neurodegeneration and AD (Figure 5). At 2 months, we detected 187 putative mRNA:miRNA pairs, of which 140 consisted on upregulated target genes and downregulated miRNAs, and 47 on downregulated target genes and upregulated miRNAs (Figure 5). At 9 months, we detected 61 putative mRNA:miRNA pairs, of which 21 consisted on upregulated target genes and downregulated miRNAs, and 40 on downregulated target genes and upregulated miRNAs (Figure 5). Only 3 putative mRNA:miRNA pairs were found at both 2 and 9 months of age (Figure 5). Complete lists of upregulated or downregulated mRNAs and miRNAs that are part of putative mRNA:miRNA pairs at 2 and 9 months of age are listed separately in Tables 2, 3, respectively. Table 4 summarizes mRNA:miRNA integrative analysis at different ages.

**Table 2:** Messenger RNAs differentially expressed in the hippocampus of SAMP8 that belong to mRNA:miRNA predicted pairs.

**2-month-old SAMP8**				**9-month-old SAMP8**	
**Upregulated mRNA**			**Downregulated mRNA**	**Upregulated mRNA**	**Downregulated mRNA**
**Gene symbol/Accession number/z-score**			**Gene symbol/Accession number/z-score**	**Gene symbol/Accession number/z-score**	**Gene symbol/Accession number/z -score**
*Adam10*/NM_007399.1/2.2	*Nr6a1*/NM_010264.4/3.84	*Srf*/NM_020493.1/2.60	*Adss*/NM_007422.3/−2.18	*Ncoa3*/NM_008679.3/2.06	*Capza2*/NM_007604.3/−2.64
*Bsn*/NM_007567.2/2.69	*Gpr37*/NM_010338.2/2.91	*Nup160*/NM_021512.2/3.08	*Ctnnd1*/NM_007615.2/−2.63	*Pbx1*/NM_008783.1/2.64	*Cav1*/NM_007616.4/−2.06
*Crcp*/NM_007761.2/2.89	*Hoxa5*/NM_010453.1/2.81	*Rhot1*/NM_021536.8/3.80	*Comt*/NM_007744.2/−2.52	*Mxd1*/NM_010751.2/2.58	*Chuk*/NM_007700.2/−2.55
*Csf3r*/NM_007782.3/2.71	*Ids*/NM_010498.1/2.55	*Midn*/NM_021565.2/2.46	*Cyp7b1*/NM_007825.4/−2.67	*Pald1*/NM_013753.2/2.89	*Dll3*/NM_007866.1/−2.59
*Ncan*/NM_007789.3/2.85	*Klf12*/NM_010636.3/3.97	*Tsc1*/NM_022887.1/1.99	*Flt4*/NM_008029.1/−2.53	*Trp53rk*/NM_023815.4/2.51	*Drd4*/NM_007878.3/−2.77
*Cyb561*/NM_007805.5/3.1	*Matn3*/NM_010770.4/3.3	*Cmtm3*/NM_024217.3/2.19	*Ltf*/NM_008522.1/−1.99	*Klhl13*/NM_026167.3/2.63	*Pdia3*/NM_007952.2/−2.50
*Elk4*/NM_007923.2/2.27	*Ndufs4*/NM_010887.2/2.6	*Tceanc2*/NM_025617.2/2.52	*Masp1*/NM_008555.2/−2.61		*Gcdh*/NM_008097.2/−2.60
*Gabrg2*/NM_008073.1/2.0	*Pik3c2a*/NM_011083.2/2.51	*Srek1ip1*/NM_026075.1/2.73	*P2rx1*/NM_008771.1/−2.51		*Gria1*/NM_008165.4/−2.10
*Gata5*/NM_008093.1/2.00	*Ppara*/NM_011144.1/2.60	*Fbxo32*/NM_026346.1/2.53	*Pcp4*/NM_008791.1/−2.47		*H3f3b*/NM_008211.2/−2.52
*Gif*/NM_008118.1/2.64	*Cyth3*/NM_011182.4/2.58	*Nacc2*/NM_026495.3/3.54	*Nptn*/NM_009145.2/−2.25		*Htr1f*/NM_008310.3/−2.51
*Slc6a9*/NM_008135.1/2.53	*Ptpre*/NM_011212.3/2.32	*Efcab2*/NM_026626.3/2.39	*Sepp1*/NM_009155.1/−4.52		*Kcnj8*/NM_008428.1/−2.08
*Hlx*/NM_008250.2/2.25	*Tcfeb*/NM_011549.1/2.06	*Cxcr4*/NM_009911.3/2.23	*Atp8a1*/NM_009727.2/−2.66		*Clpb*/NM_009191.4/−3.44
*Hmg20b*/NM_008252.3/3.1	*Tdg*/NM_011561.3/2.78	*Stx5a*/NM_019829.4/2.44	*Gabra1*/NM_010250.4/−2.57		*Itga5*/NM_010577.4/−4.12
*Hyal1*/NM_008317.5/3.83	*Ercc5*/NM_011729.2/3.06	*C1qtnf1*/NM_019959.1/2.81	*Maff*/NM_010755.4/2.32		*Itk*/NM_010583.3/−2.46
*Kif5b*/NM_008448/2.73	*Mrps12*/NM_011885.5/2.1	*Runx1*/NM_009821.3/2.00	*Man1a2*/NM_010763.1/−2.69		*Cyth3*/NM011182.4/−2.40
*Kpna1*/NM_008465/2.59	*Chst4*/NM_011998.4/2.14	*Sncg*/NM_011430.1/2.77	*Ndst2*/NM_010811.2/−3.02		*Hsp90b1*/NM_011631.2/−2.50
*Zfp239*/NM_008616.3/2.16	*Add2*/NM_013458.5/3.48		*Serf2*/NM_011354.1/−2.53		*Hs2st1*/NM_011828.4/−2.63
*Nsd1*/NM_008739.3/2.10	*Crem*/NM_013498.2/1.99		*Skil*/NM_011354.2/−2.53		*Spry4*/NM_011898.3/−2.71
*Pde7a*/NM_008802.3/2.60	*Eif4a2*/NM_013506.3/2.28		*Coro1c*/NM_011779.2/−2.76		*Map2k7*/NM_011944.3/−2.69
*Pik3c2a*/NM_011083.2/2.5	*Mmp9*/NM_013599.1/2.00		*Abca1*/NM_013454.3/−2.12		*Fgf9*/NM_013518.4/−1.99
*Pml*/NM_008884.5/3.57	*Pam*/NM_013626.3/2.05		*Atp6v0d1*/NM_013477.1/−2.58		*Atp11a/*NM_015804.1/−2.60
*Pou3f2*/NM_008899.2/2.06	*Pax6*/NM_013627.6/2.00		*Tcp1*/NM_013686.4/−3.76		*Mapk6*/NM_015806.1/−2.00
*Rab23*/NM_008999.4/2.60	*Sh3yl1*/NM_013709.5/2.15		*Pdk4*/NM_013743.2/−3.09		*Slc5a3*/NM_017391.1/−2.00
*Ret*/NM_009050.1/2.00	*Mga*/NM_013720.1/2.22		*Pbx3*/NM_016768.4/−2.05		*Socs6*/NM_018821.4/−2.58
*Rfng*/NM_009053.2/1.99	*Rasal1*/NM_013832.1/2.0		*Hp*/NM_017370.1/−1.99		*Thsd1*/NM_019576.2/−2.77
*Sema5a*/NM_009154.2/2.48	*Slc33a1*/NM_015728.1/2.0		*Pdlim4*/NM_019417.3/−2.78		*B3galtn1*/NM_020026.4/−2.17
*Snca*/NM_009221.2/2.37	*Dkk3*/NM_015814.3/2.65		*Ykt6*/NM_019661.4/−2.79		*Kcna4*/NM_021275.4/−2.98
*Tarpb2*/NM_009319.3/2.77	*Galns*/NM_016722.4/2.39		*Kox17*/NM_021559.2/−2.85		*Txnip*/NM_023719.2/−2.00
*Tbxa2r*/NM_009325.4/2.70	*Dnajc5*/NM_016775.1/2.0		*Mesdc2*/NM_023403.1/−2.71		*Vps50*/NM_024260.5/−3.17
*Tgfbr1*/NM_009370.3/2.31	*Irf6*/NM_016851.2/2.03		*Tmem167*/NM_025335.2/−2.85		*Arl4d*/NM_025404.3/−2.01
*Thrb*/NM_009380.3/2.96	*Slc5a3*/NM_017391.1/2.5		*Serbp1*/NM_025814.2/−2.14		*Cdip1*/NM_025670.2/−1.99
*Unc5c*/NM_009472.4/2.50	*Chst2*/NM_018763.2/2.12		*Lipt2*/NM_026010.1/−2.52		*Mmachc*/NM_025962.3/−3.23
*Nrsn1*/NM_009513.2/3.48	*Cldn8*/NM_018778.3/2.35		*Atg12*/NM_026217.2/−2.66		*Ergic1*/NM_026170.3/−2.14
*Zhx1*/NM_009572.4/2.24	*Grpbp2*/NM_019581.1/2.5		*Loh12cr1*/NM_026371.1/−2.78		*Chac2*/NM_026527.3/−2.44
*Neurod6*/NM_009717.1/2.53	*Rbms2*/NM_019711.1/2.9		*Mrpl19*/NM_026490.1/−2.86		*Cttnbp2nl*/NM_030249.4/−3.37
*Atp2b2*/NM_009723.6/2.16	*Plp2*/NM_019755.5/3.43		*Manba*/NM_027288.4/−2.62		*Gpr63*/NM_030733.3/−1.99
*C3ar1*/NM_009779.1/2.64	*Amotl2*/NM_019764.2/2.59		*Trps1*/NM_032000.2/−2.49		*Dnajc3*/NM_008929.3/−2.10
*Casp6*/NM_009811.4/3.55	*Cyp2d22*/NM_019823.4/3.6		*Kremen1*/NM_032396.2/−2.54	
*En1*/NM_010133.2/2.56	*Pnkd*/NM_019999.2/2.21			
*Esr2*/NM_010157.3/2.56	*Insm2*/NM_020287.1/2.65			

**Table 3:** MicroRNAs differentially expressed in the hippocampus of SAMP8 that belong to mRNA:miRNA predicted pairs.

**2-month-old SAMP8**		**9-month-old SAMP8**	
**Upregulated microRNAs**	**Downregulated microRNAs**	**Upregulated microRNAs**	**Downregulated microRNAs**
miRNA/Fold Change/p-value	miRNA/Fold Change/p-value	miRNA/Fold Change/p-value	miRNA/Fold Change/p-value
*mmu-let-7b-5p*/ 1.311 /0.025	*mmu-miR-107-3p*/0.616/0.039	*mmu-miR-128-3p*/1.400/0.049	*mmu-let-7b-5p*/0.711/0.046
*mmu-let-7e-5p*/1.505/0.050	*mmu-miR-132-3p*/0.836/0.049	*mmu-miR-140-5p*/2.617/0.047	*mmu-let-7c-5p*/0.580/0.020
*mmu-miR-148b-3p*/1.384/0.020	*mmu-miR-134-5p*/0.622/0.00031	*mmu-miR-148b-3p*/1.498/0.045	*mmu-let-7d-5p*/0.707/0.0222
*mmu-miR-151-3p*/1.211/0.031	*mmu-miR-146a-5p*/0.436/0.060	*mmu-miR-342-3p*/1.09/0.049	*mmu-let-7e-5p*/0.738/0.054
*mmu-miR-298-5p*/1.34/0.042	*mmu-miR-181a-5p*/0.341/0.014	*mmu-miR-98-5p*/3.820/0.034	
	*mmu-miR-181d-5p*/0.625/0.000603	*mmu-miR-107-3p*/1.397/0.049	
	*mmu-miR-24-3p*/0.716/0.035		
	*mmu-miR-26b-5p*/0.263/0.0082		
	*mmu-miR-27a-3p*/0.715/0.0051		
	*mmu-miR-29a-3p*/0.171/0.014		
	*mmu-miR-30e-5p*/0.788/0.048		
	*mmu-miR-7a-5p*/0.611/0.030		
	*mmu-miR-92a-3p*/0.668/0.035		

**Table 4:** mRNAs and microRNAs from significant mRNA:miRNA predicted pairs and their associated Top 10 biological processes after pathway enrichment analysis.

	**MicroRNAs**	**Target mRNAs**	**Top 10 GO Biological process**	***p*-value**	**Z.score**	**Combined score**
**2-MONTH-OLD SAMP8**						
miRNAs up: mRNAs down	*mmu-miR-298-5p, mmu-miR-148-5p, mmu-miR-151-3p, mmu-let-7b-5p, mmu-let-7e-5p*	*Adss, Ctnnd1, Comt, Cyp7bp1, Flt4, Ltf, Masp1, P2rx1, Pcp4, Nptn, Sepp1, Slc17a1, Atp8a1, Gabra1, Maff, Man1a2, Ndst2, Serf2, Skil, Coro1c, Abca1, Atp6v0d1, Tcp1, Pdk4, Pbx3, Hp, Pdlim4, Ykt6, Kox17, Mesdc2, Tmem167, Serpb1, Lipt2, Atg12, Loh12cr1, Mrpl19, Manba, Trps1, Kremen1*	Regulation of membrane lipid distribution (GO:0097035)	0.00260	−2.53	4.08
			Cellular response to extracellular stimulius (GO:0031668)	0.00299	−2.26	3.66
			Regulation of extrinsic apoptotic signaling pathway via death domain receptors (GO:1902041)	0.00555	−2.37	3.33
			Wnt signaling pathway (GO:0016055)	0.01844	−2.26	3.18
			Brain development (GO:0007420)	0.01021	−2.25	3.16
			Neuron-neuron synaptic transmission (GO:0007270)	0.00891	−2.05	2.88
			Insuline receptor signaling pathway (GO: 0008286)	0.04929	−1.97	2.77
			Cellular ion homeostasis (GO:0006873)	0.07075	−1.96	2.76
			Positive regulation of neuron projection development (GO:0010976)	0.04696	−1.89	2.65
			Locomotory behavior (GO:0007626)	0.07133	−1.83	2.57
miRNAs down: mRNAs up	*mmu-miR-92a-3p, mmu-miR-24-3p, mmu-miR-27a-3p, mmu-miR-30e-5p, mmu-miR-107-3p, mmu-miR-26b-5p, mmu-miR-7a-5p, mmu-miR-181a-5p, mmu-miR-181d-5p, mmu-miR-132-3p, mmu-miR-146a-5p, mmu-miR-134-5p, mmu-miR-29a-3p*	*Adam10, Bsn, Crcp, Csf3r, Ncan, Cyp561, Elk4, Gabrg2, Gata5, Gif, Slc6a9, H3f3b, Hlx, Hmg20b, Hyal1, Kif5b, Kpna1, Zfp239, Nsd1, Pde7a, Pik3ca, Pml, Pou3f2, Rab23, Ret, Rfng, Sema5a, Snca, Tarpb2, Tbx2r, Tgfbr1, Thrb, Unc5c, Nrsn1, Zhx1, Neurod6, Atp2b2, C3ar1, Casp6, Runx1, Cxcr4, En1, Esr2, Nr6a1, Gpr37, Hoxa5, Ids, Klf12, Matn3, Ndufs4, Pik3c2a, Ppara, Cyth3, Ptpre, Sncg, Tfeb, Tdg, Ercc5, Mrps12, Chst4, Add2, Crem, Eif4a2, Mmp9, Pam, Pax6, Sh3yl1, Mga, Rasal1, Slc33a1, Dkk3, Galns, Dnajc5, Irf6, Slc5a3, Chst2, Cldn8, Grpbp2, Rbms2, Plp2, Amotl2, Cyp2d22, Stx5a, C1qtnf1, Pnkd, Insm2, Srf, Nup160, Rhot1, Midn, Tsc1, Cmtm3, Tceanc2, Srek1ip1, Fbxo32, Nacc2, Efcab2*	Embryonic morphogenesis (GO:0048598)	0.00079	−2.40	6.34
			Brain development (GO:0007420)	0.00591	−2.13	4.17
			Synapse organization (GO:0050808)	0.00501	−2.06	4.07
			Neuron migration (GO:0001764)	0.00583	−2.05	4.01
			Neuron development (GO:0048666)	0.00672	−2.06	3.97
			Behavior (GO:0007610)	0.01035	−2.18	3.60
			Regulation of synaptic transmission (GO:0050804)	0.01713	−2.04	3.16
			Response to oxygen levels (GO:0070482)	0.02114	−2.09	3.11
			Cellular senescence (GO:0090398)	0.01385	−1.90	2.98
			Inflammatory response (GO:0006954)	0.02707	−1.91	2.65
miRNAs up: mRNAs down	*mmu-miR-107-3p, mmu-miR-140-5p, mmu-miR-148b-3p, mmu-miR-342-3p, mmu-miR-98-5p, mmu-miR-128-3p*	*Capza2, Cav1, Chuk, Dll3, Drd4, Pdia3, Gcdh, Gria1, H3f3b, Htr1f, Kcnj8, Dnajc3, Clpb, Itga5, Itk, Cyth3, Hsp90b1, Hs2st1, Spry4, Map2k7, Fgf9, Atp11a, Mapk6, Slc5a3, Socs6, Thsd1, B3galtn1, Kcna4, Txnip, Vps50, Arl4d, Cdip1, Mmachc, Ergic1, Chac2, Cttnbp2nl, Gpr63*	Memory (GO:0007613)	0.00138	−2.23	4.11
			Response to tumor necrosis factor (GO:0034612)	0.00247	−2.31	3.96
			Synaptic transmission (GO:007268)	0.03559	−2.32	3.88
			Activation of innate immune response (GO:0002218)	0.00574	−2.21	3.70
			Learning or memory (GO:0007611)	0.01189	−2.20	3.63
			Cognition (GO:0050890)	0.01703	−2.16	3.57
			Positive regulation of canonical Wnt signaling pathway (GO:0090263)	0.00920	−2.11	3.49
			Cellular response to unfolded protein (GO:0034620)	0.01849	−2.01	3.32
			Neuron-neuron synaptic transmission (GO:0007270)	0.00891	−2.01	3.32
			Response to carbohydrate (GO:0009743)	0.04411	−1.89	3.11
**9-MONTH-OLD SAMP8**						
miRNAs down: mRNAs up	*mmu-let-7b-5p, mmu-let-7c-5p, mmu-let-7d-5p, mmu-let-7e-5p*	*Ncoa3, Pbx1, Mxd1, Pald1, Trp53rk, Klhl13*	Positive regulation of cell cycle G2/M phase transition (GO:1902751)	0.00420	−2.74	9.30
			Positive regulation of G2/M transition of mitotic cell cycle (GO:0010971)	0.00420	−2.74	9.29
			Positive regulation of mitotic cell cycle (GO:0045931)	0.02884	−2.15	6.24
			Intracellular steroid hormone receptor signaling pathway (GO:0030518)	0.02059	−2.07	6.09
			Negative regulation of sequence-specific DNA binding transcription factor activity (GO:0043433)	0.03514	−2.10	5.95
			Histone acetylation (GO:0016573)	0.02829	−2.05	5.93
			Developmental growth (GO:0048589)	0.04520	−2.16	5.83
			Negative regulation of neuron differentiation (GO:0045665)	0.04085	−2.15	5.83
			Positive regulation of cell cycle process (GO:0090068)	0.06054	−2.29	5.74
			Negative regulation of neurogenesis (GO:0050768)	0.05032	−2.14	5.56

**Figure 6:**
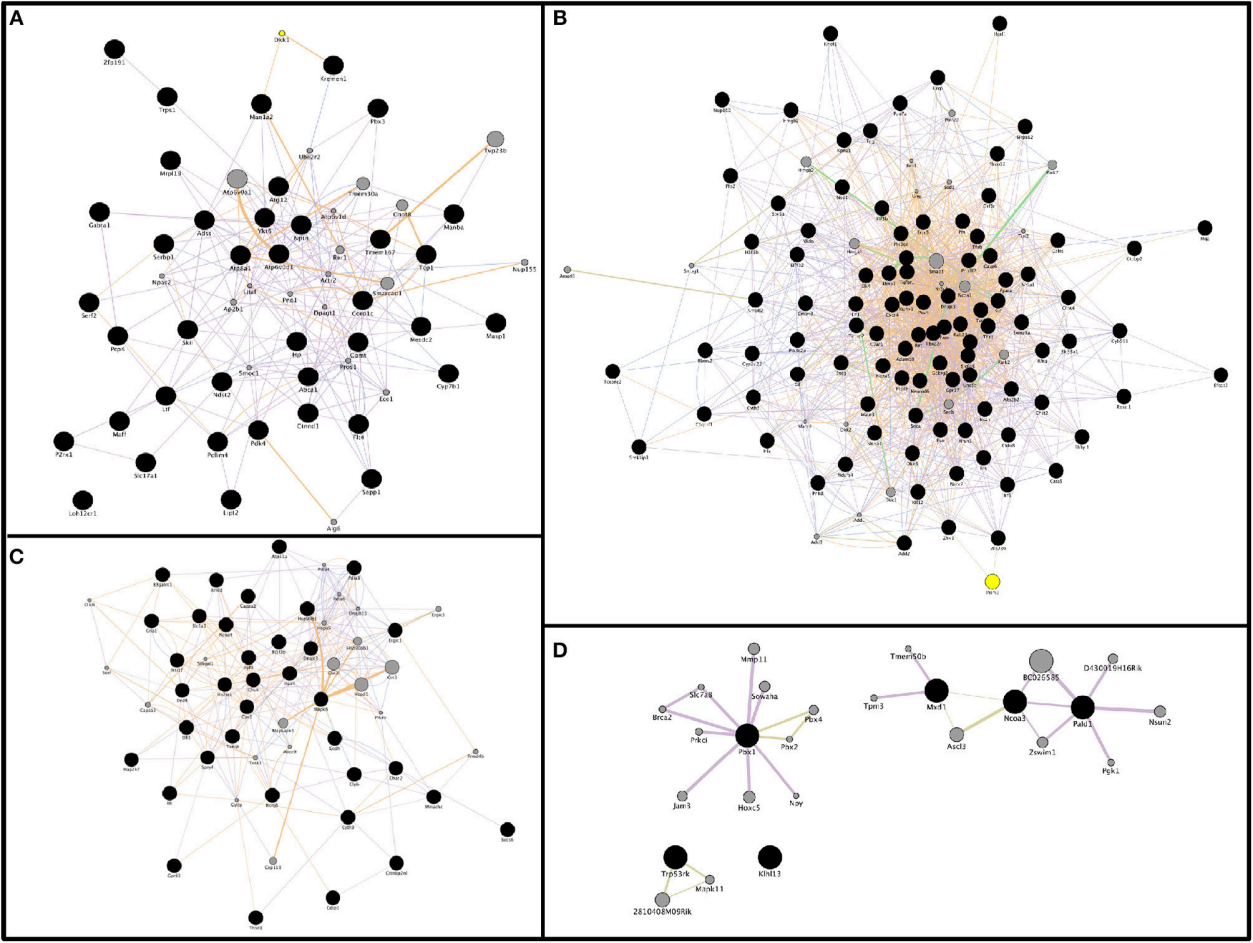
Representative gene interaction networks of microRNAs target genes (from mRNA:miRNA predicted pairs) built with GeneMANIA App. Network of 39 genes for the condition miRNAs up: messenger RNAs (mRNAs) down in 2-month-old SAMP8 mice **(A)**, network of 97 genes for the condition miRNAs down: mRNAs up in SAMP8 mice at 2 months of age **(B)**, network of 37 genes for the condition of miRNAs up: mRNAs down in 9-month-old SAMP8 mice **(C)**, and network of 6 genes for the condition miRNAs down: mRNAs up in SAMP8 mice at 9 months of age **(D)**. Genes-of-interest are represented as black circles, and their related genes as gray circles. Genetic interactions are displayed as light green lines, predicted related genes as orange lines, physical interactions as red lines, co-localization as blue lines, shared protein domains as dark green lines, and co-expression as violet lines. Node size is proportional to the number of edges (interactions, represented as lines) for each circle.

To better understand the impact of miRNA perturbation on gene expression, mRNA:miRNA regulatory networks were constructed based on these predicted mRNA:miRNA interaction pairs using GeneMania Cytoscape app (Santiago and Potashkin, 2014). Enrichment terms were scored by *p*-value, Z-score, and combined score. Thus, interaction networks were mapped and visualized for each condition (upregulated miRNAs and downregulated mRNAs, or downregulated miRNAs and upregulated mRNAs) at both 2 and 9 months of age. Figures 6A,B show two regulatory networks altered in 2-month-old SAMP8 mice based on the interactions between 6 upregulated miRNAs and their 491 downregulated targets (Figure 6A), and on 22 downregulated miRNAs and their 571 upregulated targets (Figure 6B). On the other hand, Figures 6C,D show two regulatory networks altered in 9-month-old SAMP8 based on the interactions between 13 upregulated miRNAs and their 687-downregulated targets (Figure 6C), and on 4 downregulated miRNAs, and their 346-upregulated targets (Figure 6D).

### Functional Categories Based on Gene Ontology and Biological Pathways

To integrate the functions of the potentially affected mRNAs, we performed pathway enrichment analysis on the different networks using Gene Ontology (GO) database. The results show that at 2 months the main associated GO cell processes or pathways correspond to brain development (GO: 0007420); Wnt signaling pathway (GO: 0016055); regulation of extrinsic apoptotic signaling (GO: 1902041); insulin receptor signaling (GO: 00082686); embryonic morphogenesis (GO: 0048598); calcium ion homeostasis (GO: 0055074); neuronal development (GO: 0048666); neuronal migration (GO: 0001764) and behavior (GO: 0007610); response to oxygen levels (GO: 0070482); cellular senescence (GO: 0090398), and inflammatory response (GO: 0006954). On the other hand, at 9 months, the main associated GO cell processes or pathways correspond to: memory (GO: 0007613); synaptic transmission (GO: 0007268); activation of the innate immune response (GO: 0002218); learning or memory (GO: 0007611); cognition (GO: 0050890); neuron-neuron synaptic transmission (GO: 0007270); histone acetylation (GO: 0016573); negative regulation of neuronal differentiation (GO: 0045665), and negative regulation of neurogenesis (GO: 0050768), among others. Remarkably, all these pathways are critically involved in aging, neurodegeneration, and AD (listed in Table 4 and Supplementary Material 5).

### Validation of a Representative Subset of mRNA:miRNA Pairs Involved in Brain Aging and Neurodegeneration

**Figure 7:**
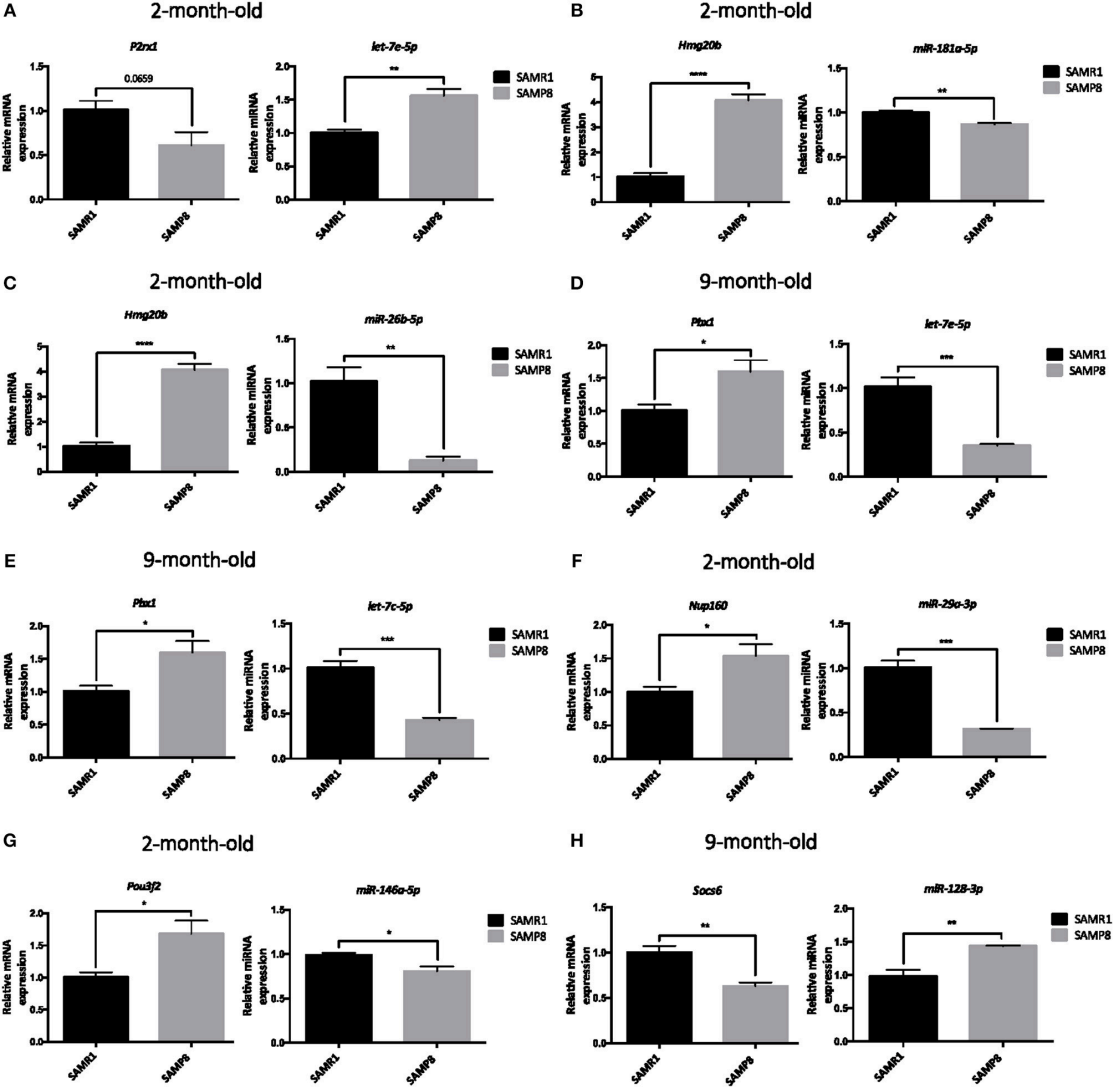
Validation of a representative subset of mRNA:miRNA pairs involved in the brain aging and neurodegeneration. Relative expression of *let-7c-5p, miR-181a-5p, miR-26b-5p, let-7e-5p, miR-29a-3p, miR-146a-5p, and miR-128-3p* in the hippocampus of 2 and/or 9 months of age SAMP8 and age-matched SAMR1 mice **(A–H)**. Relative expression of *P2rx1, Hmg20b, Pbx1, Nup160, Pou3f2*, and *Socs6* in the hippocampus of 2- or 9-month-old SAMP8 and age-matched SAMR1 mice **(A–H)**. mRNAs and miRNAs expression was measured by real-time PCR analysis from hippocampal mRNA and small RNA fractions, and expressed relative to *U6 snRNA* transcript or β*-actin* levels, respectively (*n* = 4–5/group). Mean ± standard error from five independent experiments performed in duplicates are represented. Student's *T*-test results are indicated as ^*^p < 0.05; ^**^p < 0.01; ^***^p < 0.001 and; ^****^p < 0.0001.

To validate previous results from microarray and miRNA PCR array, expression levels of a representative subset of mRNA:miRNA pairs from Table 5 were measured by single real-time PCR in hippocampal samples from 2- and 9-month-old SAMP8 and SAMR1 mice. After bibliographic research to concrete pairs with the highest association with neurodegeneration and aging, eight mRNA:miRNA pairs were chosen for validation. The selected mRNA:miRNA pairs correspond to *P2rx1:let-7e-5p, Hmgb20:miR-181a-5p, Hmgb20:miR-26b-5p, Pbx1:let-7e-5p, Pbx1:let-7c-5p, Nup160:miR-29a-3p, Pou3f2:miR-146a-5p*, and *Socs6:miR-128-3p*. Notably, real-time PCR results demonstrated a strong inverse correlation between mRNA:miRNA selected pairs and results were coincident with microarray and miRNA PCR array data (Table 5 and Figures 7A–H).

**Table 5:** Results of *T*-test analysis for the real-time PCR validation of mRNA:miRNA pairs in the hippocampus of 2- and 9-month-old SAMP8 mice.

			***T*****-test**	
	**miRNA**	***N*/group**	***t***	***P*-value**
2 months	*Let-7e-5p*	4	5.048	0.002
	*miR-26b-5p*	4	5.457	0.002
	*miR-29a-3p*	4	8.992	<0.001
	*miR-146a-5p*	4	3.059	0.022
	*miR-181a-5p*	4	5.107	0.002
9 months	*Let-7c-5p*	4	7.032	<0.001
	*Let-7e-5p*	4	6.336	<0.001
	*miR-128-3p*	4	4.784	0.003
	*miR-191*	4	29.79	<0.0001
	**mRNA**			
2 months	*Hmg-20b*	4	10.95	<0.001
	*Nup160*	4	2.842	0.047
	*Pou3f2*	4	3.093	0.037
	*P2rx1*	4	2.245	0.066
9 months	*Pbx1*	4	2.976	0.025
	*Socs6*	4	4.737	0.003

### Brain-Derived Neurotrophic Factor (BDNF) Is Diminished in 9-Month-Old SAMP8 Mice

**Figure 8:**
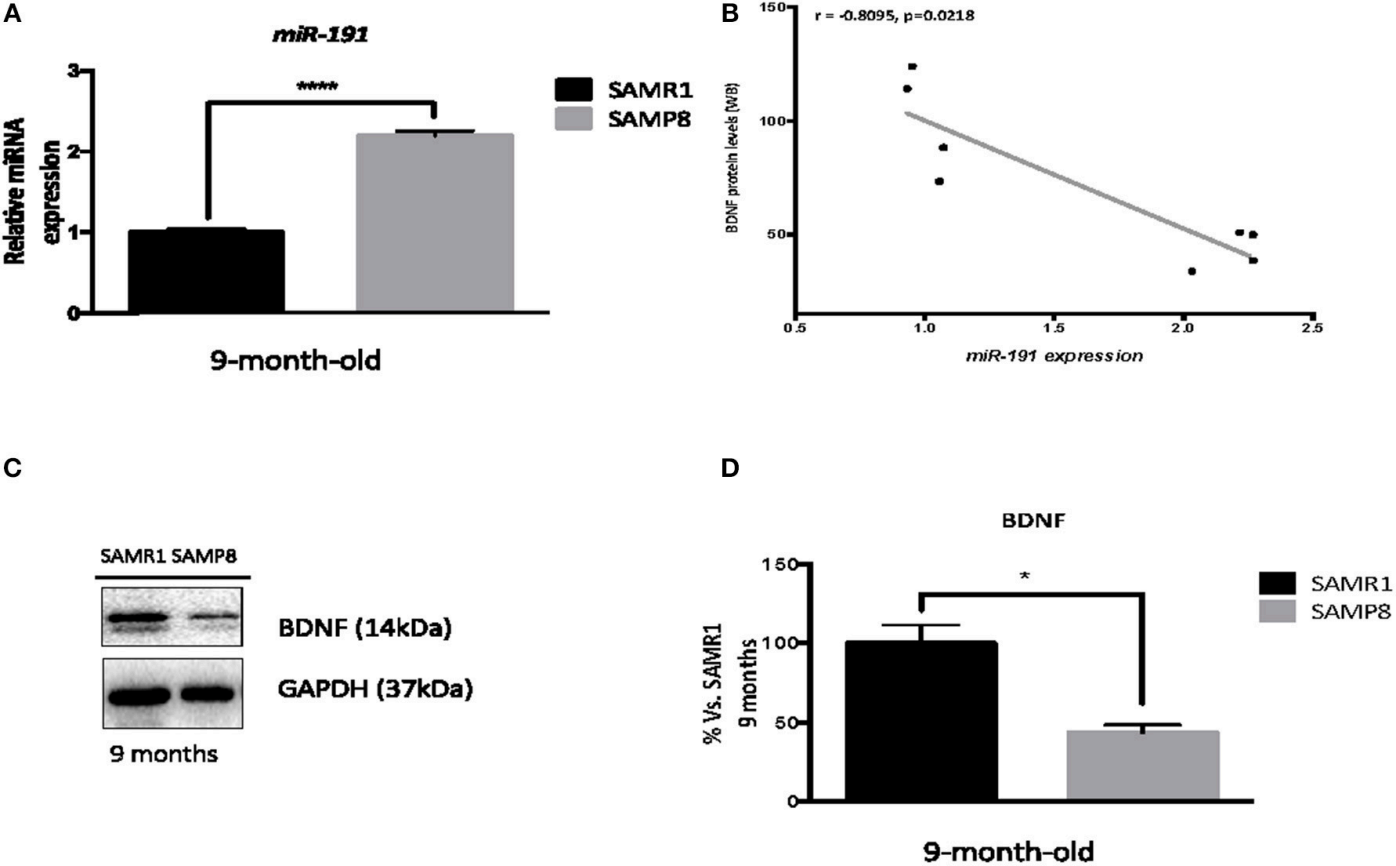
Brain-Deprived Neurotrophic Factor dysregulation in the hippocampus of 9-month-old SAMP8 mice. Validation of hippocampal *miR-191* expression in 9-month-old SAMP8 and age-matched SAMR1 mice **(A)**. Correlation between BDNF protein levels and *miR-191* gene expression **(B)**. Representative Western blot (Wb) for determination of BDNF protein levels in 9-month-old SAMP8 and age-matched SAMR1 mice **(C)**, and quantification **(D)**. MicroRNA expression was measured by real-time PCR analysis from hippocampal small RNAs fraction and expressed relative to *U6 snRNA* transcript levels (*n* = 4–5/group). Spearman correlation was performed between protein levels of BDNF and *miR-191* gene expression in both groups (*n* = 16). *R* and *p*-value were indicated on graphs. Mean ± standard error from three independent experiments performed in duplicates is represented; Values in Wb bar graphs are adjusted to 100% for protein levels of 9-month-old SAMR1. Student's *T*-test results are indicated as ^*^p < 0.05 and ^****^p < 0.001.

Since 9-month-old SAMP8 mice present dysregulated levels of *miR-191*, a miRNA known to target *Bdnf* mRNA, we proceeded to confirm this potential regulatory relationship. *miR-191* upregulation by single real-time PCR in 9-month-old hippocampus samples from SAMP8 and SAMR1 mice was validated (*T-*test, *p* < 0.0001; Figure 8A). Consistent with PCR experiments, a significant reduction of BDNF protein levels was found in 9-month-old SAMP8 mice compared to SAMR1 mice (*T-*test, *p* < 0.05; Figures 8C–D). In addition, Spearman's correlation analysis revealed a strong negative correlation between BDNF protein levels and *miR-191*, further supporting this regulatory association (*r* = −0.8095, *p* < 0.05, Figure 8B).

## Discussion

The present study aimed to evaluate the global epigenetic landscape, as well as the contribution of miRNAs on the unique AD-like and age-related phenotype of SAMP8 mice. Importantly, taking into account that women have a higher risk of developing AD and females are underrepresented in animal models of the disease (Beery and Zucker, 2010; Lin and Doraiswamy, 2015; Nebel et al., 2018; Sohn et al., 2018) the current study was performed in female SAMP8 mice.

We assessed global levels of DNA 5-mC/5-hmC mark, histone H3 and H4 acetylation and their regulatory enzymes, as well as the expression profiles of mRNAs and miRNAs in the hippocampus of 2 and 9 months of age SAMP8 female mice compared to control SAMR1 mice. In brief, we identified temporal changes in hippocampal transcriptional profiles, epigenetic marks, and mRNA:miRNA regulatory pairs and networks.

Aberrant 5-mC and 5-hmC is linked to neurodegeneration and AD by altering gene expression, thereby altering neuronal function (Delgado-Morales and Esteller, 2017; Stricker and Götz, 2018). 2-month-old SAMP8 mice showed reduced global 5-mC levels, downregulation of *Dnmt1* and *Dnmt3a*, and upregulation of *Dnmt3b* gene expression. Since the loss of 5-mC is a well-known hallmark of aging brain and the loss of DNMT1 and DNMT3a proteins is known to trigger a cognitive decline in mice (Xu, 2015), these findings may indicate a premature impact of DNA methylation disturbances on SAMP8 mice cognition.

At 9 months of age, 5-mC levels were reduced in SAMR1 mice as expected due to aging, whereas in SAMP8 mice did not differ from those at 2 months of age. *Dnmt1* was slightly upregulated in SAMR1 at 9 months, which could be a potential compensatory mechanism. However, *Dnmt1* and *Dnmt3a* remained lower in 9-month-old SAMP8 mice, and *Dnmt3b* gene expression was reduced to SAMR1 levels, suggesting that the dysregulation of this enzyme is a feature of early stages, possibly as an attempt to reestablish 5-mC levels.

5-hmC is mainly involved in the up-regulation of gene expression and, recent studies have highlighted its potential role in neurodegenerative disorders (Sherwani and Khan, 2015). The levels of 5-hmC in the brain increase progressively during aging (Chouliaras et al., 2012). For instance, it has been reported that accelerated aging and transgenic AD mice models exhibit an increase in hippocampal 5-hmC levels, which could affect DNA repair and neurogenesis (Chouliaras et al., 2012; Yokoyama et al., 2017). Consistently, we observed higher global 5-hmC levels in the hippocampus of SAMP8 mice compared to SAMR1 mice at both ages. In contrast, we did not observe a regulation of 5-hmC by age in SAMR1, possibly because in this particular model 9-month-old mice are too young to manifest this age-related hallmark. Future studies should include older mice to better understand the dynamics of this epigenetic mark along the aging process. Regarding the enzymatic machinery, *Tet1* gene expression was downregulated in 2- and 9 -month-old SAMP8, whereas *Tet2* gene expression was only decreased at 9 months of age. These results are in line with one study reporting a reduction of hippocampal *TET1* and *TET2* gene expression in aged-mice (Jessop and Toledo-Rodriguez, 2018). Moreover, loss of TET2 has been reported to be associated with regenerative decline while restoring its levels rescues neurogenesis and enhances cognition in the hippocampus of mice. Therefore, the increase of this enzyme in SAMR1 might constitute a natural protective mechanism against age-related cognitive impairment (Gontier et al., 2018), which is absent in SAMP8 mice. Future research could assess changes in these enzymes at older stages to see if SAMR1 mice fail to maintain high *Tet2* levels as age-related cognitive impairment progresses. On the other hand, we found discrepancies with our previous results in 5xFAD mice model, which had lower levels of 5-hmC at 2 and 8 months of age, as well as opposite changes in the expression of epigenetic machinery (DNMTs and TETs) (Griñán-Ferré et al., 2016). These differences among strains highlight the importance of genetic background and age when studying the epigenetic contribution to pathological aging and AD.

Histone acetylation, which is essential for hippocampal gene expression, synaptic plasticity, and memory, decreases with aging and lower levels are associated with neurodegenerative diseases such as AD (Lu et al., 2015). Several studies have shown that HDAC class I inhibitors exhibit neuroprotective effects in AD mouse models, but the role of specific HDACs regarding learning and memory in age-related diseases is still controversial (Yang et al., 2017). Here, we observed that H3 and H4 acetylation levels were lower in 2-month-old SAMP8 mice compared to age-matched SAMR1. Consistently, *Hdac1* and *Hdac2* gene expression was higher in 2-month-old SAMP8 mice compared to age-matched SAMR1. This increase of HDACs mRNA levels could contribute to the early loss of acetylation marks as a mechanism underlying the pathological phenotype of the strain. Compared to the same group of mice at 2 months of age, 9-month-old SAMR1 mice showed a downregulation of H3 acetylation levels, which is considered a feature of normal aging, together with an upregulation of *Hdac1*. For its part, SAMP8 showed further downregulation of *Hdac1* at 9 months of age. These findings reinforce the role of this HDAC on age-related histone acetylation loss. Regarding the Sirtuin protein family, we found reduced gene expression of *Sirt1* in SAMP8 mice at both ages, further confirming our former results (Cosín-Tomás et al., 2014). SIRT1 knockout mice are associated with cognitive deficits and alterations in synaptic plasticity (Michán et al., 2010). *Sirt6* gene expression was also downregulated at 9-month-old SAMP8 mice compared to age-matched SAMR1. Notably, Kaluski et al. reported severe behavioral and cognitive impairments in SIRT6 KO mice, together with increased Tau hyperphosphorylation (Kaluski et al., 2017) as occurs in SAMP8 mice (Casadesús et al., 2012).

These findings provide further evidence that alterations in acetylation enzymes have an impact on chromatin remodeling which might be contributing to SAMP8 mice pathological phenotype. Future research should explore locus-specific epigenetic changes in genes identified here and/or in other studies to establish potential mechanisms and markers of age-related cognitive decline and AD.

It is well-known that changes in mRNAs and their respective pathways have been associated with the aging process and aging-related disease (Cao et al., 2010; Deschênes and Chabot, 2017). Here, we showed dramatic changes in mRNA expression consisting on 1,062 genes dysregulated at an early age, and 1,033 at a later stage (aproximately 9% of the total genes), of which 92 genes were concurrently dysregulated in SAMP8 mice compared to SAMR1 mice at both ages. This suggests that expression of a number of genes is differentially regulated in SAMP8 mice across time. Additionally, integrating data from other relevant transcriptional studies on SAMP8, we found that only 7 genes were commonly dysregulated in SAMP8 females and males, whereas up to 22 genes were concurrently dysregulated when comparing studies performed in males only. These data support the sex differences in gene expression involved in learning and memory and illustrate the complexity underlying sex and gender differences in neurodegenerative processes (Bundy et al., 2017), reinforcing the need to include both sexes in research studies. However, it is important to note that differences in the age of animals could explain part of the gene expression profile differences between studies.

In contrast to mRNAs, little is known about changes in miRNAs and their target genes in age-related cognitive decline and AD (Dorval et al., 2013). For this reason, there is an increasing interest in miRNAs as potential biomarkers and therapeutic targets for age-related cognitive decline and AD (Cosín-Tomás et al., 2017). The miRNA array results revealed that a considerable percentage of the 84 studied miRNAs are altered in SAMP8 vs. SAMR1 mice at 2 months (33.3%) and at 9 months of age (20.2%). Six of these miRNAs (*let-7b-5p, let-7e-5p, miR-101b-3p, miR-107-3p, miR-148b-3p, miR-151-3p*) were equally altered at both ages, although the overlap did not reach the statistical significance. Notably, a number of these miRNAs were predicted to regulate the expression of genes detected with the microarray analysis. At 2 months, our integrative analysis of mRNAs and miRNAs allowed us to identify 187 putative mRNA:miRNA pairs, most of which consisted of upregulated target genes and downregulated miRNAs. In contrast, at 9 months, we identified 61 putative mRNA:miRNA pairs, most of which consisted of downregulated target genes and upregulated miRNAs. Interestingly, only 6 miRNAs and 3 mRNA:miRNA pairs were altered at both ages. This distinct temporal pattern of mRNA:miRNA pairs suggests that the progression to brain degeneration might be partly orchestrated by the participation of different miRNAs across time.

Two mRNA:miRNA regulatory networks were constructed for each age based on the predicted mRNA:miRNA interaction pairs. The enrichment analysis with all mRNAs comprised in the regulatory networks confirmed that altered mRNA:miRNA pairs found likely have an impact on pathways involved in aging, neurodegeneration and, AD (Table 4 and Supplementary Material 5).

Finally, a representative subset of mRNA:miRNA pairs were successfully validated by RT-PCR: *P2rx1:let-7e-5p; Pou3f2:miR-146a-5p; Hmgb20:miR-26b-5p*, and *Pbx1:let-7c-5p* in 2-month-old mice; and *Pbx1:let-7e-5p; Hmgb20:miR-181a-5p; Nup160:miR-29a-3p;* and *Socs6:miR-128-3p* in 9-month-old mice. These miRNAs, therefore, could be considered early-onset miRNAs involved in post-transcriptional age-related dysregulations persistent throughout adulthood.

miRNA *let-7e-5p* was upregulated, whereas its target gene, a purinergic receptor P2X, ligand-gated ion channel *(P2rx1)* was downregulated in 2-month-old SAMP8. The let-7 family is associated with neurological diseases, such as Parkinson Disease (PD), Amyotrophic Lateral Sclerosis (ALS), and AD, among others (Grasso et al., 2014). Moreover, it has been found that miRNAs of this family regulate Amyloid Precursor Protein (APP) levels in an AD model (Niwa et al., 2008). It is well-known that purinergic signaling plays an important role in neurotransmission and neuromodulation, being involved in pathophysiological processes such as neuroinflammation and ROS production in AD and other neurodegenerative diseases (Burnstock, 2017). In fact, there is an increasing interest in developing P2X receptors antagonists as a therapy for these kinds of disorders (Woods et al., 2016). As widely reported SAMP8 mice display ROS as early as 2 months of age (Griñán-Ferré et al., 2016), the downregulation of P2X receptor by *let-7e-5p* could be an early mechanism of the brain to prevent inflammation and ROS production. In contrast, in 9-month-old SAMP8, *let-7c-5p* and *let-7e-5p* were significantly downregulated, while their target gene *Pbx1* was upregulated. *Pbx1*, which encodes a nuclear protein that belongs to the PBX homeobox family of transcriptional factors, is involved in neurogenesis during development and adulthood (Grebbin et al., 2016). Of note, a general downregulation of let-7 family members, as well as an increase in *Pbx1* gene expression, has been previously described in AD patients (Maes et al., 2009; Bennett and Keeney, 2018).

Another miRNA that plays a role in cognitive function and its alteration is associated with brain disorders is *miR-128* (Lin et al., 2011; Ching and Ahmad-Annuar, 2015). Notably, overexpression of *miR-128* correlates with impaired amyloid degradation in patients with sporadic AD (Tiribuzi et al., 2014) and its target *Socs6*, which encodes a cytokine-signaling suppressor (SOCS), is involved in inflammatory processes and its expression is decreased in the β-amyloid peptide (Aβ)- and inflammatory-stimulated microglia (Walker et al., 2015). *miR-128* upregulation and reduced *Socs6* gene expression was found in 9-month-old SAMP8 mice, suggesting that this mRNA:miRNA pair contribute to the chronic inflammation characteristic of the SAMP8 phenotype.

Interestingly, a downregulation of both *miR-181a-5p* and *miR-26b-5p*, and an upregulation of their target gene *Hmg20b* were found in 2-month-old SAMP8. *miR-26b-5p* is dysregulated in the brain of sporadic AD patients and is known to be involved in Tau and apoptosis pathophysiological processes (Hébert et al., 2008; Hu et al., 2016), while *miR-181-a-5p* is involved in hippocampus-dependent memory formation (Zhang et al., 2017) and is also downregulated in CSF and brain of AD patients (Cogswell et al., 2008). *Hmg20b* (or *Braf35*) gene encodes a DNA-binding protein involved in neuronal differentiation that forms complexes with other transcriptional and epigenetic factors mediating repression of neuron-specific genes (Hakimi et al., 2002; Ceballos-Chávez et al., 2012). These findings highlight a potential early epigenetic regulation of *Hmg20b* that can be related to gene expression alterations reported in the brain of SAMP8 mice.

*miR-146a-5p* has been proposed to play a role in the early pro-inflammatory response of AD by downregulating complement factor H, interleukin-1 receptor, and tetraspanin-12 and found to be upregulated in the hippocampus of AD patients at early stages; consistently, we found increased levels of this miRNA in 2-month-old SAMP8. Moreover, we detected a downregulation of its target gene *Pou3f2*, which is a transcription factor highly expressed in post-mitotic neurons (Hagino-Yamagishi et al., 1997). Interestingly, this factor is involved in neuron differentiation and interacts with other AD-related proteins involved in endoplasmic reticulum stress (Huang et al., 2005). *miR-29a-3p*, a brain-enriched miRNA known to modulate BACE1 expression was upregulated in SAMP8 mice at 2 months of age. The association between this miRNA and AD pathology has been controversial since it has been found to be both upregulated and downregulated in AD patient's tissues by different authors (Miya Shaik et al., 2018). In this study, an upregulation of this miRNA and a downregulation of one of its target genes, *Nup160* was determined. *Nup160* encodes a key component of the nuclear pore complex, which mediates nucleoplasmic transport. Age-related defects in NUP160 and the nuclear pore complex has been proposed to contribute to abnormal protein trafficking, and in turn to neurodegenerative diseases (Woulfe et al., 2002; D'Angelo et al., 2009).

BDNF is an important neurotrophic factor involved in neuroprotection. It has been described to be regulated by histone acetylation, DNA methylation, and miRNAs. Thus, it can be considered a representative example of the complex regulatory network formed by these three mechanisms. *miR-191* expression, which is known to control BDNF gene expression (Nagpal et al., 2013; Varendi et al., 2014) was found upregulated in aged SAMP8 correlating with decreased BDNF protein levels. These findings support the existence of an effective epigenetic control of the *Bdnf* gene, likely influencing brain function and cognitive capabilities in SAMP8 mice. In the light of all results presented here, it can be hypothesized that changes in 5-mC and histone acetylation levels in the hippocampus might also be contributing to the repression of *Bdnf* gene (Boulle et al., 2012; Koppel and Timmusk, 2013).

Overall, we identified temporal alterations in epigenetic marks, chromatin-modifier enzymes, and miRNAs that appear to be carefully orchestrating hippocampal gene expression changes along the onset and development of pathological aging features and AD-like pathology in SAMP8 mice. We hypothesize that these gene expression changes are affecting processes related to neurodegeneration such as OS, inflammation, APP processing, Tau hyperphosphorylation, abnormal protein trafficking, cell cycle dysregulation, neurogenesis, Endoplasmic Reticulum (ER) stress, and behavioral changes among others, which may lead to SAMP8 senescent and AD-like phenotype. This study points to an interplay between epigenetic mechanisms and gene networks that seems to be relevant for the progression toward a pathological aging. At the same time, it provides several potential markers as well as therapeutic candidates within the epigenetic landscape and miRNA profile of SAMP8 to prevent or delay the onset of age-related brain dysfunction, at least in the hippocampus. Importantly, most of these epigenetic marks, enzymes, and microRNAs are reported to be sensitive to pharmacological and environmental interventions (Alegría-Torres et al., 2011; Szyf, 2015; Christopher et al., 2016; Griñan-Ferré et al., 2016a,b,c). Thus, future studies could estimate the therapeutic potential of these interventions for preventing or delaying neurodegenerative diseases by focusing on these targets using the SAMP8 strain. Finally, since SAMP8 strain is a model of accelerated aging that presents some AD-like features, our results might be interpreted with caution (and general conclusions on AD in humans should be avoided).

## Author Contributions

CG-F, MC-T, MA-L, JC-A, and CG-C carried out the experimental intervention and genomic experiments. CG-F, MC-T, and DO-S analyzed the data. CG-F, MC-T, DO-S, PK, and MP drafted the manuscript. MP, PK, and DO-S supervised the study. All authors read and approved the final manuscript.

### Conflict of Interest Statement

The authors declare that the research was conducted in the absence of any commercial or financial relationships that could be construed as a potential conflict of interest.
