# Supplementary material for: Temporal Integrative Analysis of mRNA and microRNAs Expression Profiles and Epigenetic Alterations in Female SAMP8, a Model of Age-Related Cognitive Decline
Source: Front Genet. 2018 Dec 11;9:596. doi: 10.3389/fgene.2018.00596 (PMC6297390; doi:10.3389/fgene.2018.00596)
Supplement: Supplementary file 1 [file Table_1.docx]

**Supplementary material 1.** Antibodies used in Western blot studies.

| Antibody | Host | Source/Catalog | WB dilution |
| --- | --- | --- | --- |
| Acetyl Histone H3 | Rabbit | Millipore/06-599 | 1:1000 |
| Acetyl Histone H4 | Sheep | R&D Systems/AF5215 | 1:1000 |
| TBP | Mouse | Abcam/51841 | 1:1000 |
| BDNF | Rabbit | Santa Cruz Biotech/ H-117 | 1:500 |
| GAPDH | Mouse | Millipore/MAB374 | 1:5000 |
| Donkey-anti-goat HRP conjugated |  | Santa Cruz Biotech/ sc-2020 | 1:3000 |
| Goat-anti-mouse HRP conjugated |  | Biorad/# 170-5047 | 1:2000 |
| Rabbit-anti-sheep HRP conjugated |  | Abcam/ab97130 | 1:2000 |
| Goat-anti-rabbit HRP conjugated |  | Cell Signaling/# 7074 | 1:2000 |
