# Supplementary material for: Temporal Integrative Analysis of mRNA and microRNAs Expression Profiles and Epigenetic Alterations in Female SAMP8, a Model of Age-Related Cognitive Decline
Source: Front Genet. 2018 Dec 11;9:596. doi: 10.3389/fgene.2018.00596 (PMC6297390; doi:10.3389/fgene.2018.00596)
Supplement: Supplementary file 2 [file Table_2.docx]

**Supplementary material 2**. Primers and probes used in real-time qPCR studies.

SYBR-Green primers

| Target | Product size (bp) | Forward primer (5’-3’) | Reverse primer (5’-3’) |
| --- | --- | --- | --- |
| *Dnmt1* | 85 | ACCTGGAGAGCAGAAATGGC | TGAAAGGGTGTCACTGTCCG |
| *Dnmt3b* | 142 | TGCCAGACCTTGGAAACCTC | GCTGGCACCCTCTTCTTCAT |
| *Tet1* | 188 | CTGCCAACTACCCCAAACTCA | TCGGGGTTTTGTCTTCCGTT |
| *Tet2* | 113 | CCATCATGTTGTGGGACGGA | ATTCTGAGAACAGCGACGGT |
| *Hdac1* | 150 | TCACCGAATCCGCATGACTC | TCTGGGCGAATAGAACGCAG |
| *Hdac2* | 280 | CTATCCCGCTCTGTGCCCT | GAGGCTTCATGGGATGACCC |
| *Sirt1* | 229 | AACACACACACAAAATCCAGCA | TGCAACCTGCTCCAAGGTAT |
| *Sirt2* | 248 | TGCAGGAGGCTCAGGATTC | GTCACTCCTTCGAGGGTCAG |
| *Sirt6* | 150 | GTCTCACTGTGTCCCTTGTCC | GCGGGTGTGATTGGTAGAGA |
| *Nup160* | 172 | TGTGGGGGAAATGACACTAAC | GCGTTGGCGTGTAAGTTGTT |
| *Pou3f2* | 118 | TTCTGCCCGGACTCAGTTTC | TGTAGTCGAGGGGAGACAGG |
| *P2rx1* | 249 | AATGGGACAAACCGTCGTCA | GTCACGTTCACCCTCCCCAG |
| *Hmgb2* | 222 | AGAGGTGAAAAGAGCCGGGAAG | TCTGGAACAGAAGCCGGCAA |
| *Pbx1* | 191 | CCAACTCAGCGGGTGGATAC | CACGGGTCAGGTAGAAGAAGAC |
| *Socs6* | 239 | CCTTCAGTACACCGTGCCTT | GGCTCTGCAACATGACTCCT |
| *β-actin* | 218 | CTGTCCCTGTATGCCTCTG | ATGTCACGCACGATTTCC |

Taqman probes

| Target | | Product size (bp) | Reference |
| --- | --- | --- | --- |
| *Dnmt3a* | 58 | | Mm00432881_m1 |
| *Tbp* | 93 | | Mm00446971_m1 |
| *mmu-let-7c* | - | | 478577_mir |
| *mmu-let-7e* | - | | 478579_mir |
| *mmu-miR-26b* | - | | Mmu482965_mir |
| *mmu-miR-29a* | - | | Mmu481032_mir |
| *mmu-miR-29c* | - | | Mmu481034_mir |
| *mmu-miR-128* | - | | Mmu480912_mir |
| *mmu-miR-146a* | - | | Mmu478399_mir |
| *mmu-miR-181a* | - | | Mmu481485_mir |
| *mmu-miR-191* | - | | Mmu481584_mir |
| *U6 snRNA* | - | | 001973 |
