# Supplementary material for: Temporal Integrative Analysis of mRNA and microRNAs Expression Profiles and Epigenetic Alterations in Female SAMP8, a Model of Age-Related Cognitive Decline
Source: Front Genet. 2018 Dec 11;9:596. doi: 10.3389/fgene.2018.00596 (PMC6297390; doi:10.3389/fgene.2018.00596)
Supplement: Supplementary file 3 [file Table_3.docx]

Supplementary material 3. mRNAs differentially expressed in the hippocampus of SAMP8 compared to SAMR1 at 2 and 9 months of age.

| 2 months – mRNAs up in SAMP8 (571) | | | | |
| --- | --- | --- | --- | --- |
| Cy3 | **Cy5** | **Accesion Number** | **Gene Symbol** | **Zscore** |
| 2312 | 3685 | **NM_010293.3** | **Gykl1** | **5,21** |
| 9400 | 18463 | **NM_026850.4** | **Pdcl3** | **5,10** |
| 2501 | 3667 | **NM_175121.4** | **Slc38a2** | **4,86** |
| 8316 | 14775 | **NM_023153.3** | **Cwc15** | **4,84** |
| 12135 | 27410 | **NM_025318.3** | **Emc6** | **4,80** |
| 3685 | 5257 | **NM_025493.2** | **1700018B24Rik** | **4,64** |
| 6036 | 10057 | **NM_011807.3** | **Dlg2** | **4,62** |
| 2808 | 4149 | **NM_008902.3** | **Endou** | **4,52** |
| 1579 | 2155 | **NM_022327.5** | **Ralb** | **4,51** |
| 962 | 1256 | **NM_013859.2** | **Znhit2** | **4,48** |
| 5965 | 8512 | **NM_028270.4** | **Aldh1b1** | **4,34** |
| 1875 | 2391 | **NM_016854.2** | **Ppp1r3c** | **4,26** |
| 473 | 606 | **NM_010636.3** | **Klf12** | **3,97** |
| 3754 | 4833 | **NM_016859.3** | **Bysl** | **3,93** |
| 426 | 596 | **NM_027585.2** | **Cnbd2** | **3,92** |
| 2092 | 2776 | **NM_013601.2** | **Msx2** | **3,89** |
| 1156 | 1592 | **NM_010264.4** | **Nr6a1** | **3,84** |
| 2522 | 3264 | **NM_008317.5** | **Hyal1** | **3,83** |
| 1048 | 1320 | **NM_029612.4** | **Slamf9** | **3,81** |
| 2370 | 2881 | **NM_021536.8** | **Rhot1** | **3,80** |
| 2084 | 2834 | **NM_013684.3** | **Tbp** | **3,73** |
| 1842 | 2629 | **NM_029054.1** | **Slc25a54** | **3,70** |
| 777 | 973 | **null** | **Zfp125** | **3,68** |
| 5707 | 7248 | **NM_016909.2** | **Tsnax** | **3,63** |
| 3184 | 3958 | **NM_019823.4** | **Cyp2d22** | **3,60** |
| 895 | 1137 | **NM_025411.4** | **Pithd1** | **3,59** |
| 2925 | 3686 | **NM_021391.3** | **Ppp1r1a** | **3,58** |
| 2633 | 3311 | **NM_001277867.1** | **Mphosph9** | **3,58** |
| 5966 | 7577 | **NM_008884.5** | **Pml** | **3,57** |
| 17806 | 29414 | **NM_009811.4** | **Casp6** | **3,55** |
| 1137 | 1353 | **NM_026495.3** | **Nacc2** | **3,54** |
| 2438 | 3037 | **NM_009513.2** | **Nrsn1** | **3,48** |
| 4856 | 6249 | **NM_013458.5** | **Add2** | **3,48** |
| 30237 | 42962 | **null** | **1700003G18Rik** | **3,47** |
| 13582 | 21155 | **NM_028333.2** | **Angptl1** | **3,46** |
| 4182 | 5347 | **NM_139198.2** | **Plac8** | **3,46** |
| 2065 | 2776 | **null** | **2610008G14Rik** | **3,44** |
| 975 | 1220 | **NM_019755.5** | **Plp2** | **3,43** |
| 8888 | 12612 | **NM_023879.3** | **Rpgrip1** | **3,43** |
| 9113 | 13731 | **NM_011755.2** | **Zfp35** | **3,42** |
| 3167 | 4260 | **NM_011933.2** | **Decr2** | **3,41** |
| 23439 | 34991 | **NM_027925.3** | **Trnau1ap** | **3,40** |
| 525 | 721 | **NM_021480.5** | **Tdh** | **3,38** |
| 2135 | 2664 | **NM_011438.2** | **Sox12** | **3,38** |
| 7749 | 9966 | **null** | **Cdk19os** | **3,36** |
| 4251 | 5355 | **NM_010054.2** | **Dlx2** | **3,35** |
| 2749 | 3604 | **NM_019935.3** | **Ovol1** | **3,31** |
| 3620 | 4532 | **NM_010770.4** | **Matn3** | **3,31** |
| 7702 | 9798 | **NM_030261.4** | **Sesn3** | **3,31** |
| 1667 | 2124 | **NM_153577.2** | **Syne4** | **3,30** |
| 1687 | 2118 | **NM_029788.5** | **Rnft1** | **3,29** |
| 3921 | 5052 | **NM_145417.3** | **Rnpep** | **3,28** |
| 7039 | 9019 | **NM_001256158.1** | **4933402D24Rik** | **3,28** |
| 2078 | 2757 | **NM_007857.5** | **Dhh** | **3,28** |
| 8544 | 11604 | **null** | **4930558G05Rik** | **3,27** |
| 1867 | 2295 | **null** | **4930565D16Rik** | **3,25** |
| 20203 | 29104 | **NM_026933.2** | **Triap1** | **3,25** |
| 12013 | 18858 | **NM_001081063.2** | **Prss55** | **3,24** |
| 895 | 1115 | **NM_183271.2** | **Wfdc15a** | **3,23** |
| 2766 | 3571 | **null** | **1700016L21Rik** | **3,21** |
| 7142 | 9638 | **NM_013767.6** | **Csnk1e** | **3,21** |
| 1745 | 2257 | **NM_138301.2** | **Trpm2** | **3,20** |
| 413 | 520 | **NM_197991.2** | **Emc10** | **3,19** |
| 1946 | 2326 | **NM_009967.2** | **Crygs** | **3,19** |
| 11432 | 16280 | **NM_026940.4** | **Ydjc** | **3,18** |
| 83 | 191 | **NM_010875.3** | **Ncam1** | **3,16** |
| 6357 | 8091 | **NM_009344.3** | **Phlda1** | **3,16** |
| 2580 | 3150 | **NM_029324.3** | **Tex48** | **3,15** |
| 1542 | 1885 | **null** | **A930008L05Rik** | **3,14** |
| 493 | 636 | **NM_008954.3** | **Pspn** | **3,13** |
| 2793 | 3438 | **NM_007805.5** | **Cyb561** | **3,11** |
| 12008 | 16122 | **NM_023525.2** | **Cad** | **3,10** |
| 12149 | 17063 | **NM_008252.3** | **Hmgb2** | **3,10** |
| 5028 | 6543 | **NM_027373.2** | **Afap1** | **3,10** |
| 1834 | 2208 | **null** | **9430052A13Rik** | **3,09** |
| 2812 | 3539 | **NM_021512.2** | **Nup160** | **3,08** |
| 13394 | 18013 | **NM_026975.2** | **Bola1** | **3,08** |
| 5123 | 6723 | **NM_011729.2** | **Ercc5** | **3,06** |
| 9998 | 13767 | **NM_010922.2** | **Mrpl40** | **3,06** |
| 1633 | 1919 | **NM_133232.3** | **Pfkfb3** | **3,05** |
| 1689 | 2056 | **NM_029245.3** | **Ankrd53** | **3,04** |
| 1750 | 2117 | **NM_023290.3** | **Mkrn2** | **3,02** |
| 1216 | 1446 | **NM_011651.2** | **Tsks** | **3,02** |
| 14171 | 18418 | **NM_026306.3** | **Trmt112** | **3,00** |
| 6149 | 7824 | **NM_025692.3** | **Uba5** | **3,00** |
| 4975 | 6148 | **null** | **9030616G12Rik** | **2,99** |
| 9161 | 12740 | **NM_026484.4** | **Ccny** | **2,98** |
| 4427 | 5371 | **NM_009380.3** | **Thrb** | **2,96** |
| 197 | 319 | **NM_001009544.2** | **4921501E09Rik** | **2,96** |
| 6187 | 7971 | **NM_009402.2** | **Pglyrp1** | **2,95** |
| 5418 | 6988 | **NM_010188.5** | **Fcgr3** | **2,94** |
| 9322 | 13384 | **NM_026604.4** | **Fam135a** | **2,93** |
| 1155 | 1317 | **NM_153516.2** | **Bcl2l13** | **2,93** |
| 460 | 597 | **NM_032005.4** | **Tbx19** | **2,93** |
| 3072 | 3691 | **NM_027928.1** | **Chst13** | **2,93** |
| 2356 | 2726 | **NM_010338.2** | **Gpr37** | **2,91** |
| 4409 | 5587 | **NM_011398.3** | **Slc25a14** | **2,91** |
| 16238 | 25544 | **NM_025382.6** | **Maco1** | **2,91** |
| 3637 | 4662 | **NM_019626.3** | **Cbln1** | **2,91** |
| 238 | 347 | **NM_011997.3** | **Casp8ap2** | **2,89** |
| 14331 | 19322 | **null** | **null** | **2,89** |
| 574 | 696 | **NM_009540.3** | **Zfa-ps** | **2,89** |
| 6613 | 8741 | **null** | **null** | **2,89** |
| 509 | 656 | **NM_026219.2** | **Uqcrb** | **2,87** |
| 1497 | 1855 | **NM_138659.2** | **Prpf8** | **2,87** |
| 5155 | 6189 | **null** | **null** | **2,87** |
| 22642 | 33484 | **NM_008470.1** | **Krt16** | **2,87** |
| 26333 | 40589 | **NM_013647.2** | **Rps16** | **2,87** |
| 4636 | 5665 | **NM_024414.2** | **Stx1b** | **2,86** |
| 5100 | 6660 | **null** | **9430006E15Rik** | **2,86** |
| 20643 | 28261 | **null** | **1700031C06Rik** | **2,86** |
| 746 | 879 | **NM_008738.3** | **Nrtn** | **2,85** |
| 6269 | 7943 | **NM_008192.3** | **Gucy2e** | **2,85** |
| 3078 | 3682 | **NM_007789.3** | **Ncan** | **2,85** |
| 6633 | 8667 | **NM_008439.4** | **Khk** | **2,84** |
| 7295 | 9552 | **NM_029942.1** | **Prelid2** | **2,84** |
| 7691 | 9675 | **NM_019693.3** | **Ddx39b** | **2,84** |
| 3534 | 4390 | **NM_145574.3** | **Ccdc136** | **2,83** |
| 6745 | 8624 | **NM_011294.3** | **Sub1** | **2,81** |
| 2070 | 2417 | **NM_175362.2** | **Card11** | **2,81** |
| 981 | 1205 | **NM_026438.4** | **Ppa1** | **2,80** |
| 975 | 1175 | **null** | **Cby3** | **2,78** |
| 4744 | 5749 | **NM_023125.3** | **Kng1** | **2,78** |
| 6740 | 8376 | **NM_011561.3** | **Tdg** | **2,78** |
| 7864 | 9260 | **NM_152810.2** | **Cdc5l** | **2,77** |
| 117 | 240 | **NM_001031772.2** | **Lin28b** | **2,77** |
| 5772 | 6940 | **NM_023258.4** | **Pycard** | **2,77** |
| 13839 | 18243 | **NM_009319.3** | **Tarbp2** | **2,77** |
| 4027 | 4746 | **NM_015786.3** | **Hist1h1c** | **2,76** |
| 7206 | 9592 | **null** | **4921520E09Rik** | **2,76** |
| 18280 | 25870 | **NM_025302.4** | **Mrpl2** | **2,76** |
| 3059 | 3621 | **NM_030132.5** | **Utp23** | **2,75** |
| 4913 | 5790 | **null** | **4833419A21Rik** | **2,75** |
| 4735 | 5694 | **NM_175147.2** | **Etd** | **2,74** |
| 4571 | 5654 | **NM_001081400.3** | **1810013L24Rik** | **2,74** |
| 3640 | 4396 | **NM_019566.3** | **Rhog** | **2,74** |
| 1503 | 1734 | **NM_178224.3** | **Cbs** | **2,73** |
| 12160 | 15842 | **NM_024190.2** | **Chmp1b** | **2,73** |
| 8539 | 10265 | **NM_175102.4** | **Sf3b5** | **2,72** |
| 11186 | 15035 | **NM_028472.2** | **Bmper** | **2,72** |
| 1818 | 2118 | **null** | **4933439N06Rik** | **2,72** |
| 18850 | 27524 | **NM_026171.2** | **Nvl** | **2,72** |
| 2276 | 2653 | **NM_007782.3** | **Csf3r** | **2,71** |
| 118 | 240 | **NM_001081125.1** | **Gli2** | **2,71** |
| 3254 | 3771 | **NM_029561.4** | **Ndfip2** | **2,70** |
| 5631 | 6798 | **NM_009325.4** | **Tbxa2r** | **2,70** |
| 5971 | 7003 | **NM_198937.2** | **Jpt2** | **2,70** |
| 1754 | 2072 | **NM_029961.2** | **Abcb5** | **2,69** |
| 2175 | 2723 | **NM_018826.2** | **Irx5** | **2,69** |
| 17835 | 24741 | **null** | **2210411A11Rik** | **2,69** |
| 12496 | 15981 | **NM_153404.3** | **Liph** | **2,69** |
| 1315 | 1549 | **null** | **9130007G19Rik** | **2,69** |
| 6078 | 7540 | **null** | **1700016A09Rik** | **2,68** |
| 1410 | 1640 | **NM_011939.3** | **Hsf4** | **2,68** |
| 1770 | 2093 | **NM_021521.2** | **Med12** | **2,67** |
| 12591 | 16074 | **NM_133992.3** | **Pan2** | **2,67** |
| 3911 | 4713 | **NM_001361996.1** | **Tlk2** | **2,67** |
| 3106 | 3618 | **NM_007838.3** | **Ddost** | **2,66** |
| 1293 | 1503 | **NM_009437.5** | **Tst** | **2,66** |
| 1208 | 1520 | **NM_021505.3** | **Anapc5** | **2,65** |
| 2616 | 3201 | **NM_015814.3** | **Dkk3** | **2,65** |
| 1650 | 1895 | **NM_172054.4** | **Txndc9** | **2,65** |
| 8036 | 10457 | **null** | **4930538L07Rik** | **2,64** |
| 7159 | 8627 | **NM_011380.2** | **Six2** | **2,64** |
| 17818 | 24510 | **null** | **4930415P13Rik** | **2,64** |
| 20852 | 27198 | **null** | **4930555K05Rik** | **2,64** |
| 307 | 404 | **NM_144925.3** | **Tnrc6a** | **2,64** |
| 9425 | 12427 | **NM_030714.2** | **Dtx3** | **2,64** |
| 503 | 606 | **NM_023336.4** | **Brd3** | **2,64** |
| 8393 | 10622 | **NM_008572.1** | **Mcpt8** | **2,63** |
| 856 | 1023 | **NM_010887.2** | **Ndufs4** | **2,62** |
| 408 | 505 | **NM_008826.5** | **Pfkl** | **2,62** |
| 742 | 866 | **NM_153092.4** | **Nupl2** | **2,62** |
| 5105 | 6098 | **NM_025274.3** | **Dppa5a** | **2,62** |
| 1674 | 1924 | **NM_009757.5** | **Bmp15** | **2,61** |
| 105 | 202 | **NM_008245.3** | **Hhex** | **2,61** |
| 2477 | 2888 | **NM_021567.5** | **Pcbp4** | **2,61** |
| 5953 | 7314 | **NM_178690.4** | **Rab3gap1** | **2,60** |
| 4604 | 5527 | **null** | **2900079G21Rik** | **2,60** |
| 3453 | 4011 | **NM_182992.2** | **Mypn** | **2,60** |
| 5609 | 6853 | **NM_008999.4** | **Rab23** | **2,60** |
| 1060 | 1257 | **NM_008802.3** | **Pde7a** | **2,60** |
| 1489 | 1703 | **NM_010620.1** | **Kif15** | **2,60** |
| 7647 | 9275 | **NM_146137.3** | **Amigo1** | **2,60** |
| 4259 | 4992 | **NM_028303.4** | **Pdzd11** | **2,59** |
| 1410 | 1633 | **null** | **1700010L13Rik** | **2,59** |
| 16397 | 22188 | **NM_019764.2** | **Amotl2** | **2,59** |
| 1516 | 1841 | **NM_019831.3** | **Zmym3** | **2,59** |
| 6257 | 7785 | **NM_008474.2** | **Krt84** | **2,59** |
| 10846 | 13793 | **NM_008465.5** | **Kpna1** | **2,59** |
| 4231 | 5011 | **null** | **4930563H03Rik** | **2,59** |
| 3496 | 4184 | **null** | **5830462O15Rik** | **2,58** |
| 14272 | 20335 | **NM_138751.2** | **Tmem47** | **2,58** |
| 11003 | 14278 | **NM_026588.1** | **Stx19** | **2,57** |
| 404 | 499 | **NM_021470.6** | **Rnf32** | **2,57** |
| 2619 | 3176 | **NM_010157.3** | **Esr2** | **2,56** |
| 20802 | 26514 | **NM_026579.3** | **D10Wsu102e** | **2,56** |
| 3477 | 4240 | **NM_010133.2** | **En1** | **2,56** |
| 1440 | 1663 | **NM_026203.3** | **Ahi1** | **2,55** |
| 2109 | 2532 | **NM_025295.4** | **Btd** | **2,55** |
| 361 | 442 | **NM_010998.2** | **Olfr55** | **2,55** |
| 435 | 535 | **NM_145559.2** | **Slc2a9** | **2,55** |
| 2304 | 2734 | **NM_027800.1** | **Krtap2-4** | **2,55** |
| 3166 | 3615 | **NM_016697.3** | **Gpc3** | **2,54** |
| 10269 | 13569 | **NM_028185.2** | **Lsm11** | **2,54** |
| 909 | 1092 | **NM_172587.3** | **Cdc14b** | **2,54** |
| 5820 | 6990 | **null** | **8030443L12Rik** | **2,54** |
| 2943 | 3443 | **NM_026346.3** | **Fbxo32** | **2,53** |
| 12505 | 15772 | **null** | **6330582A15Rik** | **2,52** |
| 12229 | 17232 | **NM_029361.4** | **Wnk2** | **2,52** |
| 3518 | 4124 | **NM_028778.4** | **Nuak2** | **2,52** |
| 145 | 253 | **NM_023179.3** | **Atp6v1g2** | **2,52** |
| 10505 | 12911 | **NM_021406.5** | **Trem1** | **2,51** |
| 10462 | 13111 | **NM_028055.4** | **Btbd17** | **2,51** |
| 2614 | 3272 | **NM_011083.2** | **Pik3c2a** | **2,51** |
| 1471 | 1676 | **NM_145221.2** | **Appl1** | **2,51** |
| 11725 | 15543 | **NM_026597.3** | **Inka1** | **2,51** |
| 447 | 557 | **NM_133765.4** | **Fbxo31** | **2,50** |
| 5539 | 6673 | **null** | **C430049B03Rik** | **2,50** |
| 2009 | 2329 | **NM_009472.4** | **Unc5c** | **2,50** |
| 6416 | 7796 | **NM_010593.2** | **Jup** | **2,49** |
| 5569 | 6721 | **NM_139270.2** | **Pth2r** | **2,49** |
| 3621 | 4312 | **NM_008832.2** | **Phka1** | **2,49** |
| 7651 | 9353 | **NM_018817.2** | **Smarcal1** | **2,48** |
| 2496 | 2932 | **NM_009154.2** | **Sema5a** | **2,48** |
| 2003 | 2308 | **NM_027673.3** | **Tssk4** | **2,48** |
| 2267 | 2634 | **NM_007992.2** | **Fbln2** | **2,48** |
| 42 | 143 | **null** | **2810047C21Rik1** | **2,48** |
| 1167 | 1342 | **NM_175046.3** | **Bcor** | **2,47** |
| 6049 | 7335 | **null** | **Pip5k1bos** | **2,47** |
| 11563 | 14625 | **NM_021565.2** | **Midn** | **2,46** |
| 433 | 521 | **NM_009324.2** | **Tbx2** | **2,46** |
| 20079 | 25170 | **NM_013776.2** | **Tcl1b5** | **2,45** |
| 2606 | 2917 | **NM_011170.3** | **Prnp** | **2,45** |
| 1450 | 1674 | **NM_012020.2** | **Foxl2** | **2,45** |
| 9126 | 11450 | **NM_025654.2** | **Rdm1** | **2,45** |
| 8823 | 10458 | **NM_026860.1** | **Gkn3** | **2,45** |
| 549 | 668 | **null** | **1700045H11Rik** | **2,44** |
| 3887 | 4779 | **NM_019829.4** | **Stx5a** | **2,44** |
| 4940 | 5640 | **NM_007539.3** | **Bdkrb1** | **2,44** |
| 1101 | 1315 | **null** | **1700110I07Rik** | **2,44** |
| 2786 | 3187 | **NM_029584.1** | **Zfp773** | **2,43** |
| 3288 | 3957 | **NM_001081211.2** | **Ptafr** | **2,43** |
| 5501 | 6862 | **NM_008022.2** | **Foxd4** | **2,43** |
| 17461 | 22913 | **NM_031162.4** | **Cd247** | **2,42** |
| 743 | 866 | **NM_008704.2** | **Nme1** | **2,42** |
| 177 | 288 | **NM_021447.2** | **Trim54** | **2,42** |
| 583 | 687 | **NM_013808.4** | **Csrp3** | **2,42** |
| 17258 | 22405 | **NM_026780.3** | **Syf2** | **2,42** |
| 5445 | 6556 | **NM_058212.2** | **Dpf3** | **2,42** |
| 7161 | 9238 | **NM_009571.2** | **Zfy2** | **2,41** |
| 6453 | 8221 | **NM_028015.2** | **Cers5** | **2,41** |
| 13169 | 17569 | **NM_026871.1** | **Hint2** | **2,41** |
| 8100 | 10214 | **NM_013878.2** | **Cabp2** | **2,41** |
| 5995 | 7067 | **NM_010364.4** | **Gtf2h4** | **2,40** |
| 6001 | 7389 | **NM_007867.4** | **Dlx4** | **2,40** |
| 20145 | 24379 | **NM_019400.3** | **Rabep1** | **2,39** |
| 15424 | 20324 | **NM_001033140.3** | **Sdhaf1** | **2,39** |
| 4613 | 5438 | **null** | **1810062O18Rik** | **2,39** |
| 2427 | 2915 | **NM_016722.4** | **Galns** | **2,39** |
| 1267 | 1425 | **NM_026357.2** | **Ribc2** | **2,39** |
| 1170 | 1338 | **NM_026626.3** | **Efcab2** | **2,39** |
| 2072 | 2392 | **NM_013460.4** | **Adra1d** | **2,38** |
| 1035 | 1193 | **null** | **1110018F16Rik** | **2,38** |
| 4991 | 5930 | **NM_007758.2** | **Cr2** | **2,37** |
| 516 | 636 | **NM_009221.2** | **Snca** | **2,37** |
| 4817 | 5700 | **NM_028171.2** | **Ceacam13** | **2,37** |
| 2017 | 2376 | **NM_023739.3** | **Nfx1** | **2,36** |
| 3890 | 4464 | **NM_028815.4** | **Cep97** | **2,35** |
| 12244 | 15505 | **NM_018778.3** | **Cldn8** | **2,35** |
| 1932 | 2171 | **NM_026731.3** | **Ppp1r14a** | **2,35** |
| 895 | 1033 | **NM_178576.3** | **Cpsf4** | **2,34** |
| 1703 | 1976 | **null** | **Gm8327** | **2,33** |
| 2954 | 3418 | **NM_023867.1** | **8030498B09Rik** | **2,33** |
| 22506 | 30592 | **NM_145371.4** | **Eif2b1** | **2,33** |
| 11137 | 14597 | **NM_016736.3** | **Nub1** | **2,33** |
| 25188 | 37921 | **NM_012021.3** | **Prdx5** | **2,33** |
| 3510 | 4208 | **NM_007784.3** | **Csn1s1** | **2,33** |
| 5310 | 6535 | **NM_013841.3** | **Vps45** | **2,33** |
| 10351 | 12712 | **NM_153527.2** | **Dnajb13** | **2,33** |
| 1621 | 1832 | **NM_009546.2** | **Trim25** | **2,33** |
| 6313 | 7619 | **null** | **1700041L08Rik** | **2,32** |
| 8879 | 10794 | **NM_146072.4** | **Grik1** | **2,32** |
| 3233 | 3688 | **NM_177747.3** | **Zfp711** | **2,32** |
| 1720 | 2030 | **NM_008605.3** | **Mmp12** | **2,32** |
| 12291 | 16950 | **NM_011212.3** | **Ptpre** | **2,32** |
| 1736 | 1997 | **NM_026804.1** | **Cideb** | **2,31** |
| 372 | 465 | **NM_177889.5** | **Zfp82** | **2,31** |
| 16822 | 21863 | **NM_018860.4** | **Rpl41** | **2,31** |
| 512 | 619 | **NM_023129.5** | **Pln** | **2,31** |
| 3668 | 4321 | **NM_009370.3** | **Tgfbr1** | **2,31** |
| 4232 | 4887 | **NM_053085.2** | **Tcf23** | **2,30** |
| 178 | 260 | **NM_009708.2** | **Rnd2** | **2,30** |
| 1963 | 2444 | **NM_016843.4** | **Atxn10** | **2,30** |
| 3324 | 3843 | **NM_023695.3** | **Crybb1** | **2,28** |
| 626 | 733 | **NM_008304.2** | **Sdc2** | **2,28** |
| 4766 | 5613 | **NM_172932.4** | **Nlgn3** | **2,28** |
| 1073 | 1201 | **NM_029638.2** | **Aoc1** | **2,28** |
| 4544 | 5273 | **NM_171824.2** | **Pgbd5** | **2,28** |
| 1042 | 1185 | **NM_013506.3** | **Eif4a2** | **2,28** |
| 778 | 883 | **NM_009347.3** | **Tecta** | **2,28** |
| 6164 | 7385 | **NM_016672.4** | **Ddc** | **2,28** |
| 1137 | 1311 | **NM_001081310.2** | **Tmem236** | **2,27** |
| 3232 | 3738 | **NM_007923.2** | **Elk4** | **2,27** |
| 3885 | 5239 | **null** | **Gm16740** | **2,27** |
| 1984 | 2238 | **NM_028016.3** | **Nanog** | **2,27** |
| 2146 | 2482 | **NM_013903.2** | **Mmp20** | **2,27** |
| 6891 | 8038 | **NM_145359.2** | **Ubald1** | **2,27** |
| 23924 | 31931 | **NM_175178.4** | **Aifm3** | **2,27** |
| 354 | 421 | **null** | **4921528I07Rik** | **2,26** |
| 268 | 360 | **NM_144515.2** | **Zfp52** | **2,26** |
| 4991 | 5705 | **NM_001286028.1** | **Dlk2** | **2,26** |
| 2787 | 3237 | **NM_011405.4** | **Slc7a7** | **2,26** |
| 1115 | 1310 | **NM_008250.2** | **Hlx** | **2,25** |
| 5975 | 7200 | **NM_177643.4** | **Zfp281** | **2,25** |
| 1521 | 1712 | **NM_001081307.1** | **Ppp1r12b** | **2,25** |
| 7513 | 9037 | **NM_009015.3** | **Rad54l** | **2,25** |
| 7836 | 9427 | **NM_010846.1** | **Mx1** | **2,25** |
| 2630 | 2995 | **null** | **Speer9-ps1** | **2,25** |
| 1041 | 1175 | **NM_009358.3** | **Ppp2r5d** | **2,25** |
| 7802 | 9192 | **null** | **4933407A17Rik** | **2,24** |
| 722 | 852 | **NM_009572.4** | **Zhx1** | **2,24** |
| 2599 | 2898 | **NM_025831.3** | **Pxdc1** | **2,24** |
| 829 | 957 | **null** | **4930567H12Rik** | **2,24** |
| 1293 | 1478 | **NM_007567.2** | **Bsn** | **2,24** |
| 15408 | 19684 | **NM_028474.1** | **Ptchd4** | **2,23** |
| 1234 | 1504 | **NM_009911.3** | **Cxcr4** | **2,23** |
| 215 | 301 | **NM_009519.2** | **Wnt11** | **2,23** |
| 1399 | 1616 | **NM_013926.1** | **Cbx8** | **2,23** |
| 2188 | 2503 | **null** | **2810488O17Rik** | **2,22** |
| 1724 | 2037 | **NM_013731.3** | **Sgk2** | **2,22** |
| 1657 | 1997 | **NM_013720.2** | **Mga** | **2,22** |
| 3106,5 | 3572,4 | **NM_008458.2** | **Serpina3c** | **2,22** |
| 1700 | 2001 | **NM_021431.2** | **Nudt11** | **2,22** |
| 13560 | 17532 | **NM_028417.1** | **Ttc9b** | **2,22** |
| 7014 | 8464 | **NM_010007.4** | **Cyp2j5** | **2,22** |
| 2094 | 2440 | **NM_001013373.2** | **Tmprss13** | **2,22** |
| 5882 | 6931 | **NM_028868.3** | **Cxxc1** | **2,21** |
| 1809 | 2174 | **null** | **5430440L12Rik** | **2,21** |
| 826 | 956 | **NM_011996.2** | **Adh4** | **2,21** |
| 8122 | 10256 | **NM_007399.1** | **Adam10** | **2,21** |
| 1697 | 1915 | **NM_019999.2** | **Pnkd** | **2,21** |
| 1872 | 2155 | **NM_018864.6** | **Impa1** | **2,21** |
| 4368 | 5147 | **NM_011244.4** | **Rarg** | **2,21** |
| 2008 | 2425 | **NM_026960.4** | **Gsdmd** | **2,20** |
| 14403 | 17822 | **NM_029301.1** | **Cfap126** | **2,20** |
| 1756 | 1959 | **null** | **4930545E07Rik** | **2,20** |
| 22985 | 30238 | **NM_026154.3** | **Mrpl10** | **2,20** |
| 1569 | 1746 | **NM_013723.3** | **Podxl** | **2,20** |
| 4819 | 5533 | **NM_011929.3** | **Clcn6** | **2,19** |
| 1324 | 1506 | **NM_029979.3** | **Trim35** | **2,19** |
| 9538 | 11592 | **NM_030262.3** | **Pofut2** | **2,19** |
| 5690 | 6815 | **NM_024217.3** | **Cmtm3** | **2,19** |
| 1934 | 2245 | **NM_011370.3** | **Cyfip1** | **2,19** |
| 3671 | 4098 | **NM_008684.2** | **Neo1** | **2,19** |
| 1311 | 1494 | **null** | **LOC641025** | **2,19** |
| 13580 | 16152 | **NM_033565.2** | **Aff4** | **2,19** |
| 1984 | 2237 | **NM_001347610.1** | **Gypc** | **2,19** |
| 6059 | 7266 | **NM_010433.2** | **Hipk2** | **2,18** |
| 15723 | 19651 | **NM_030068.2** | **Iqch** | **2,18** |
| 24235 | 35841 | **null** | **5930409G06Rik** | **2,18** |
| 2572 | 2906 | **null** | **Usp46os1** | **2,18** |
| 2007 | 2277 | **NM_013484.2** | **C2** | **2,18** |
| 35422 | 44130 | **null** | **1110002L01Rik** | **2,18** |
| 25459 | 37417 | **NM_010616.3** | **Kif12** | **2,18** |
| 923 | 1078 | **NM_007811.2** | **Cyp26a1** | **2,18** |
| 2635 | 2992 | **null** | **Copg2os2** | **2,17** |
| 987 | 1143 | **null** | **C030041M11Rik** | **2,17** |
| 2781 | 3307 | **NM_153546.4** | **Mboat1** | **2,17** |
| 14471 | 17051 | **NM_028078.3** | **Igsf5** | **2,17** |
| 9540 | 11873 | **NM_025703.3** | **Tceal8** | **2,17** |
| 5764 | 6755 | **NM_026014.3** | **Cdt1** | **2,16** |
| 1783 | 2052 | **NM_015822.3** | **Fbxl3** | **2,16** |
| 7005 | 8516 | **NM_172712.2** | **Uba6** | **2,16** |
| 1939 | 2250 | **NM_008616.3** | **Zfp239** | **2,16** |
| 7906 | 10181 | **NM_009723.6** | **Atp2b2** | **2,16** |
| 5323 | 6094 | **null** | **4930555M17Rik** | **2,15** |
| 91 | 164 | **NM_029070.2** | **Tmem114** | **2,15** |
| 1382 | 1554 | **NM_033324.2** | **Dgcr8** | **2,15** |
| 750 | 840 | **NM_015818.2** | **Hs6st1** | **2,15** |
| 9582 | 11514 | **NM_013709.5** | **Sh3yl1** | **2,15** |
| 9606 | 11006 | **NM_001357661.1** | **Fcor** | **2,15** |
| 855 | 980 | **NM_029315.1** | **Pou5f2** | **2,15** |
| 39 | 109 | **NM_027462.4** | **Wars2** | **2,14** |
| 12230 | 15237 | **NM_011998.4** | **Chst4** | **2,14** |
| 22997 | 30244 | **NM_026673.4** | **Apoo** | **2,14** |
| 1503 | 1704 | **NM_021608.3** | **Dctn5** | **2,14** |
| 190 | 297 | **NM_009238.3** | **Sox4** | **2,14** |
| 8891 | 10581 | **NM_009072.2** | **Rock2** | **2,14** |
| 4428 | 5142 | **NM_009471.3** | **Umps** | **2,14** |
| 3335 | 4012 | **NM_010572.2** | **Irs4** | **2,14** |
| 4723 | 5365 | **NM_180599.1** | **Mfap3** | **2,14** |
| 1238 | 1390 | **NM_028040.2** | **Rpusd4** | **2,13** |
| 3870 | 4436 | **NM_010816.1** | **Morc1** | **2,13** |
| 993 | 1135 | **NM_198652.2** | **Hjurp** | **2,13** |
| 6043 | 7103 | **NM_021457.3** | **Fzd1** | **2,13** |
| 3158 | 3564 | **NM_080419.2** | **Igsf8** | **2,13** |
| 11642 | 15155 | **NM_022655.3** | **Ireb2** | **2,13** |
| 9106 | 10989 | **NM_025300.4** | **Mrpl15** | **2,13** |
| 982 | 1100 | **NM_001163775.2** | **Taok2** | **2,13** |
| 11664 | 15407 | **NM_009326.3** | **Tcea2** | **2,13** |
| 5939 | 6780 | **NM_198052.2** | **Tbx3** | **2,12** |
| 10433 | 12622 | **NM_030709.2** | **Tmprss5** | **2,12** |
| 2250 | 2487 | **NM_018763.2** | **Chst2** | **2,12** |
| 6917 | 8260 | **null** | **1700025A08Rik** | **2,11** |
| 2024 | 2303 | **null** | **4933431K23Rik** | **2,11** |
| 5363 | 6223 | **null** | **5730460C07Rik** | **2,11** |
| 5002 | 5877 | **NM_026865.3** | **Ptges3l** | **2,11** |
| 9153 | 11081 | **NM_026972.5** | **Cd209b** | **2,11** |
| 3044 | 3456 | **NM_023913.2** | **Ern1** | **2,11** |
| 5547 | 6406 | **NM_029928.2** | **Ptprb** | **2,11** |
| 39 | 107 | **NM_008739.3** | **Nsd1** | **2,10** |
| 1276 | 1406 | **NM_028183.1** | **1810062G17Rik** | **2,10** |
| 6331 | 7424 | **NM_008120.3** | **Gja4** | **2,10** |
| 7968 | 9158 | **NM_026081.5** | **Gprasp1** | **2,09** |
| 8049 | 9911 | **NM_146066.2** | **Gspt1** | **2,09** |
| 13327 | 17329 | **NM_030727.5** | **Slc26a5** | **2,09** |
| 3831 | 4351 | **NM_133882.2** | **C8b** | **2,08** |
| 1453 | 1654 | **null** | **5930436O19Rik** | **2,08** |
| 5417 | 6442 | **NM_183104.2** | **4931429L15Rik** | **2,08** |
| 1709 | 1911 | **NM_019542.2** | **Nagk** | **2,08** |
| 3835 | 4351 | **NM_023557.3** | **Slc44a4** | **2,08** |
| 4076 | 4674 | **NM_010510.1** | **Ifnb1** | **2,08** |
| 4600 | 5273 | **NM_054051.1** | **Pip4k2b** | **2,07** |
| 23929 | 31139 | **null** | **4930599N23Rik** | **2,07** |
| 3765 | 4382 | **NM_022409.2** | **Zfp296** | **2,07** |
| 776 | 875 | **NM_013883.2** | **Scmh1** | **2,07** |
| 18973 | 23937 | **NM_010404.3** | **Hap1** | **2,07** |
| 3514 | 4048 | **null** | **LOC641025** | **2,07** |
| 3819 | 4354 | **NM_009043.2** | **Reg2** | **2,07** |
| 19525 | 24566 | **NM_028614.3** | **Ppp2r1b** | **2,07** |
| 19033 | 23909 | **NM_011584.4** | **Nr1d2** | **2,07** |
| 5194 | 5902 | **NM_172418.2** | **Mamstr** | **2,07** |
| 1856 | 2059 | **NM_011549.3** | **Tfeb** | **2,06** |
| 4529 | 5108 | **null** | **1110038B12Rik** | **2,06** |
| 3360 | 4016 | **NM_029722.1** | **Gje1** | **2,06** |
| 3054 | 3389 | **NM_009005.3** | **Rab7** | **2,06** |
| 3319 | 3938 | **NM_026958.3** | **Slirp** | **2,06** |
| 7548 | 8952 | **NM_145908.4** | **Porcn** | **2,06** |
| 1331 | 1526 | **NM_153399.2** | **Syne1** | **2,06** |
| 12602 | 16718 | **NM_008899.2** | **Pou3f2** | **2,06** |
| 1307 | 1437 | **NM_008721.4** | **Npdc1** | **2,05** |
| 511 | 575 | **NM_012013.2** | **Figla** | **2,05** |
| 3319 | 3937 | **NM_013626.3** | **Pam** | **2,05** |
| 9739 | 10981 | **NM_011727.2** | **Xlr3c** | **2,05** |
| 3527 | 4039 | **null** | **5730512F23Rik** | **2,05** |
| 14608 | 17628 | **NM_024242.3** | **Riok1** | **2,05** |
| 4180 | 4899 | **NM_183014.1** | **Zfp184** | **2,05** |
| 2011 | 2275 | **NM_010235.2** | **Fosl1** | **2,05** |
| 21587 | 26950 | **NM_020049.4** | **Slc6a14** | **2,04** |
| 2636 | 2900 | **NM_053201.4** | **Magee1** | **2,04** |
| 1825 | 2162 | **NM_008601.3** | **Mitf** | **2,04** |
| 10560 | 13214 | **NM_009628.3** | **Adnp** | **2,04** |
| 5165 | 5839 | **NM_019482.2** | **Panx1** | **2,04** |
| 17712 | 21269 | **NM_133975.4** | **Trip12** | **2,04** |
| 2300 | 2600 | **NM_009407.2** | **Tnp1** | **2,03** |
| 2541 | 2837 | **NM_010904.3** | **Nefh** | **2,03** |
| 3383 | 4014 | **NM_010774.2** | **Mbd4** | **2,03** |
| 19062 | 24318 | **NM_011972.2** | **Poli** | **2,03** |
| 1081 | 1198 | **NM_146783.2** | **Olfr26** | **2,03** |
| 17388 | 22099 | **NM_027152.1** | **Cd164l2** | **2,03** |
| 3996 | 4664 | **NM_016851.2** | **Irf6** | **2,03** |
| 6332 | 7447 | **NM_023281.1** | **Sdha** | **2,03** |
| 13617 | 16309 | **NM_133900.4** | **Psph** | **2,03** |
| 1533 | 1714 | **NM_027231.2** | **Polr2f** | **2,03** |
| 1830 | 2160 | **NM_001128151.2** | **Cecr2** | **2,02** |
| 1648 | 1810 | **NM_026335.2** | **Lce1h** | **2,02** |
| 607 | 696 | **NM_144884.2** | **Tor1a** | **2,02** |
| 300 | 376 | **NM_011966.3** | **Psma4** | **2,02** |
| 7971 | 9872 | **null** | **9030625N01Rik** | **2,02** |
| 2670 | 2893 | **NM_001033474.2** | **Atxn7l3b** | **2,02** |
| 2433 | 2710 | **NM_028126.3** | **Strada** | **2,02** |
| 4332 | 5058 | **NM_026775.4** | **Tmed10** | **2,02** |
| 9145 | 10648 | **NM_016699.3** | **Exosc10** | **2,02** |
| 3057 | 3434 | **NM_020021.3** | **Mos** | **2,01** |
| 9567 | 11031 | **NM_013581.3** | **Cog1** | **2,01** |
| 22933 | 27632 | **NM_022033.4** | **Oxct2a** | **2,01** |
| 804 | 905 | **NM_026350.3** | **Ccdc130** | **2,01** |
| 843 | 985 | **NM_153067.2** | **Mrgpra3** | **2,01** |
| 2396 | 2659 | **NM_025669.1** | **Pnisr** | **2,01** |
| 15838 | 19286 | **NM_011885.5** | **Mrps12** | **2,01** |
| 770 | 870 | **NM_026385.4** | **Pllp** | **2,01** |
| 2946 | 3324 | **null** | **1700072O05Rik** | **2,01** |
| 4012 | 4531 | **NM_023733.3** | **Crot** | **2,01** |
| 1231 | 1380 | **NM_013627.6** | **Pax6** | **2,00** |
| 15350 | 21421 | **NM_028679.4** | **Irak3** | **2,00** |
| 3397 | 3795 | **null** | **1600019K03Rik** | **2,00** |
| 2809 | 3208 | **NM_001033122.4** | **Cd69** | **2,00** |
| 1337 | 1521 | **NM_009821.3** | **Runx1** | **2,00** |
| 1788 | 2014 | **NM_145142.2** | **Chst10** | **2,00** |
| 5886 | 6762 | **NM_028872.3** | **Inava** | **2,00** |

| 2 months – mRNA down in SAMP8 (491) | | | | |
| --- | --- | --- | --- | --- |
| Cy3 | **Cy5** | **Accesion number** | **Gene Symbol** | **Zscore** |
| 1670 | 850 | **null** | **2610016A17Rik** | **-6,02** |
| 6669 | 3036 | **NM_028336.3** | **Tmem107** | **-5,58** |
| 17582 | 7579 | **NM_008980.2** | **Ptpra** | **-5,54** |
| 12741 | 6334 | **NM_021881.2** | **Qk** | **-5,38** |
| 1285 | 816 | **null** | **4833422M21Rik** | **-5,26** |
| 30189 | 14005 | **NM_133677.1** | **Zbtb11os1** | **-4,70** |
| 409 | 256 | **NM_009155.4** | **Selenop** | **-4,52** |
| 839 | 608 | **NM_009995.2** | **Cyp21a1** | **-4,51** |
| 2314 | 1796 | **NM_009278.4** | **Ssb** | **-4,35** |
| 1582 | 1240 | **NM_029735.2** | **Eprs** | **-4,17** |
| 6443 | 4994 | **null** | **2010009K17Rik** | **-4,16** |
| 747 | 500 | **NM_020007.4** | **Mbnl1** | **-4,10** |
| 901 | 642 | **NM_016783.4** | **Pgrmc1** | **-4,02** |
| 7694 | 5483 | **NM_008306.5** | **Ndst1** | **-3,98** |
| 451 | 330 | **NM_009605.5** | **Adipoq** | **-3,97** |
| 6212 | 4969 | **null** | **4930546E12Rik** | **-3,95** |
| 21388 | 12531 | **NM_001014836.3** | **4930404N11Rik** | **-3,95** |
| 285 | 44 | **NM_010440.3** | **Hmg20b** | **-3,89** |
| 544 | 413 | **NM_013872.4** | **Pmm1** | **-3,85** |
| 13524 | 8568 | **null** | **4930527E20Rik** | **-3,83** |
| 1210 | 937 | **NM_181588.4** | **Cmbl** | **-3,83** |
| 4639 | 3761 | **null** | **2310011C19Rik** | **-3,79** |
| 499 | 350 | **NM_013686.4** | **Tcp1** | **-3,76** |
| 20475 | 13718 | **null** | **2610011I18Rik** | **-3,73** |
| 2240 | 1783 | **NM_009196.4** | **Slc16a1** | **-3,72** |
| 4450 | 3543 | **NM_009298.4** | **Surf6** | **-3,68** |
| 2886 | 2246 | **NM_080451.2** | **Synpo2** | **-3,63** |
| 10835 | 7279 | **NM_008140.3** | **Gnat1** | **-3,61** |
| 296 | 160 | **null** | **4930551O13Rik** | **-3,57** |
| 878 | 691 | **null** | **Kbtbd8os** | **-3,55** |
| 310 | 171 | **NM_009943.2** | **Cox6a2** | **-3,54** |
| 820 | 594 | **NM_029639.2** | **Plet1** | **-3,48** |
| 2575 | 1987 | **null** | **A230083N12Rik** | **-3,45** |
| 6690 | 5065 | **NM_011491.3** | **Stc2** | **-3,43** |
| 1353 | 1076 | **NM_028787.5** | **Slc35f5** | **-3,40** |
| 5373 | 4365 | **NM_144536.3** | **Cdkal1** | **-3,40** |
| 2710 | 2262 | **NM_011221.3** | **Purb** | **-3,40** |
| 10889 | 8263 | **NM_175280.3** | **Cfap61** | **-3,39** |
| 1146 | 927 | **null** | **Kcnmb4os2** | **-3,38** |
| 426 | 315 | **NM_011478.2** | **Sprr3** | **-3,36** |
| 9622 | 7638 | **NM_028947.1** | **Arl13a** | **-3,32** |
| 837 | 659 | **NM_145151.3** | **Crebzf** | **-3,29** |
| 1357 | 1134 | **null** | **4933421A08Rik** | **-3,29** |
| 4835 | 3722 | **NM_001271569.1** | **1700065D16Rik** | **-3,26** |
| 1049 | 858 | **NM_023651.5** | **Pex13** | **-3,26** |
| 180 | 40 | **NM_080847.3** | **Asb15** | **-3,25** |
| 4484 | 3628 | **NM_145598.2** | **Nxnl1** | **-3,25** |
| 6811 | 5311 | **null** | **C030004M13Rik** | **-3,24** |
| 2461 | 2026 | **NM_011249.2** | **Rbl1** | **-3,23** |
| 1615 | 1302 | **NM_008765.3** | **Orc2** | **-3,23** |
| 5680 | 4486 | **NM_053188.2** | **Srd5a2** | **-3,21** |
| 14269 | 10341 | **NM_010870.2** | **Naip5** | **-3,20** |
| 3950 | 3220 | **NM_028835.4** | **Atg7** | **-3,19** |
| 14416 | 10518 | **NM_009822.3** | **Runx1t1** | **-3,17** |
| 6823 | 5266 | **null** | **2310039F13Rik** | **-3,15** |
| 1015 | 811 | **NM_026370.2** | **Kat8** | **-3,14** |
| 1778 | 1508 | **NM_011835.2** | **Katna1** | **-3,13** |
| 287 | 141 | **NM_172430.4** | **Sphkap** | **-3,11** |
| 4236 | 3537 | **null** | **1700084E18Rik** | **-3,09** |
| 210 | 112 | **NM_013743.2** | **Pdk4** | **-3,09** |
| 325 | 180 | **NM_053103.5** | **Entpd7** | **-3,08** |
| 4929 | 4027 | **NM_019460.2** | **Sfmbt1** | **-3,08** |
| 1068 | 864 | **NM_007996.2** | **Fdx1** | **-3,05** |
| 1844 | 1507 | **NM_008423.2** | **Kcnd1** | **-3,04** |
| 2560 | 2035 | **null** | **LOC67527** | **-3,03** |
| 11468 | 8787 | **NM_009646.2** | **Aire** | **-3,02** |
| 497 | 398 | **NM_010811.2** | **Ndst2** | **-3,02** |
| 2322 | 1877 | **null** | **2900084C01Rik** | **-2,99** |
| 4060 | 3279 | **null** | **1700013M08Rik** | **-2,96** |
| 1389 | 1159 | **NM_029104.1** | **Mss51** | **-2,95** |
| 8878 | 7084 | **NM_031868.2** | **Ppp1ca** | **-2,94** |
| 2200 | 1861 | **NM_008114.3** | **Gfi1b** | **-2,93** |
| 2812 | 2449 | **NM_027142.1** | **Zfp33b** | **-2,93** |
| 6645 | 5335 | **NM_029457.3** | **Senp2** | **-2,92** |
| 359 | 233 | **NM_011934.4** | **Esrrb** | **-2,92** |
| 5928 | 4893 | **NM_001145537.1** | **4930544D05Rik** | **-2,91** |
| 2989 | 2377 | **NM_010419.4** | **Hes5** | **-2,91** |
| 9216 | 7074 | **null** | **4930451A11Rik** | **-2,91** |
| 4815 | 4056 | **null** | **4930599N24Rik** | **-2,90** |
| 1069 | 871 | **NM_019426.2** | **Atf7ip** | **-2,90** |
| 1370 | 1154 | **NM_175174.4** | **Klhl5** | **-2,89** |
| 1253 | 1070 | **NM_023742.2** | **Dtx2** | **-2,87** |
| 1610 | 1360 | **NM_023881.4** | **Retnlb** | **-2,87** |
| 11395 | 8719 | **NM_011381.4** | **Six3** | **-2,86** |
| 797 | 610 | **NM_011300.3** | **Rps7** | **-2,85** |
| 1483 | 1264 | **NM_007642.4** | **Cd28** | **-2,85** |
| 1798 | 1597 | **NM_007459.3** | **Ap2a2** | **-2,85** |
| 4247 | 3486 | **NM_026404.2** | **Slc35a4** | **-2,84** |
| 406 | 311 | **null** | **1700016B01Rik** | **-2,84** |
| 2115 | 1771 | **NM_028478.3** | **Rassf6** | **-2,82** |
| 3555 | 2883 | **null** | **Ighg** | **-2,80** |
| 513 | 401 | **NM_010176.4** | **Fah** | **-2,80** |
| 132 | 36 | **NM_134438.3** | **Gpr37l1** | **-2,79** |
| 3034 | 2637 | **NM_026317.2** | **Prss37** | **-2,79** |
| 4061 | 3355 | **NM_025610.3** | **Asrgl1** | **-2,78** |
| 5494 | 4561 | **null** | **4930595D18Rik** | **-2,78** |
| 5846 | 4679 | **NM_144543.2** | **Thyn1** | **-2,78** |
| 1887 | 1550 | **NM_177296.5** | **Tnpo3** | **-2,77** |
| 229 | 47 | **NM_023046.4** | **Asb1** | **-2,77** |
| 3282 | 2876 | **null** | **Dancr** | **-2,77** |
| 611 | 501 | **NM_009877.2** | **Cdkn2a** | **-2,76** |
| 602 | 455 | **NM_198957.2** | **Rbm12b2** | **-2,75** |
| 1442 | 1220 | **NM_153792.2** | **Traf7** | **-2,75** |
| 4379 | 3780 | **NM_008024.2** | **Foxl1** | **-2,74** |
| 363 | 243 | **NM_013475.4** | **Apoh** | **-2,73** |
| 6119 | 5224 | **NM_008169.3** | **Grin1** | **-2,72** |
| 31802 | 22369 | **NM_027355.2** | **Rnf168** | **-2,71** |
| 199 | 50 | **NM_010220.4** | **Fkbp5** | **-2,70** |
| 2087 | 1703 | **null** | **Stamos** | **-2,70** |
| 2882 | 2441 | **NM_025768.2** | **Grtp1** | **-2,70** |
| 1852 | 1603 | **NM_011258.2** | **Rfc1** | **-2,69** |
| 256 | 141 | **NM_010715.2** | **Lig1** | **-2,68** |
| 2034 | 1710 | **NM_175180.3** | **Wdr44** | **-2,68** |
| 4843 | 3855 | **NM_001360951.1** | **Gm21814** | **-2,67** |
| 8730 | 7095 | **NM_007426.4** | **Angpt2** | **-2,67** |
| 287 | 185 | **NM_033476.3** | **Tfcp2** | **-2,66** |
| 7900 | 6337 | **NM_001033176.1** | **Slc6a21** | **-2,66** |
| 2327 | 1986 | **NM_026612.4** | **Ndufb2** | **-2,65** |
| 4182 | 3393 | **NM_019825.3** | **Ncoa6** | **-2,65** |
| 944 | 817 | **null** | **5830407P18Rik** | **-2,64** |
| 106 | 28 | **NM_011211.3** | **Ptprd** | **-2,64** |
| 924 | 786 | **null** | **2610035D17Rik** | **-2,64** |
| 12663 | 10096 | **NM_033175.3** | **Lce3c** | **-2,64** |
| 7050 | 5750 | **NM_026084.2** | **3110070M22Rik** | **-2,64** |
| 1226 | 1048 | **NM_009544.2** | **Zfp105** | **-2,64** |
| 5835 | 4930 | **NM_011655.5** | **Tubb5** | **-2,62** |
| 26809 | 19971 | **NM_024413.2** | **Plekhf1** | **-2,62** |
| 2404 | 2096 | **null** | **5430434I15Rik** | **-2,61** |
| 4282 | 3584 | **null** | **Wbscr25** | **-2,61** |
| 1561 | 1333 | **NM_183264.4** | **Tespa1** | **-2,61** |
| 101 | 27 | **NM_008657.3** | **Myf6** | **-2,61** |
| 5428 | 4573 | **NM_028892.4** | **Spag17** | **-2,61** |
| 2813 | 2376 | **NM_028219.1** | **Cnfn** | **-2,59** |
| 2137 | 1795 | **null** | **1700020G17Rik** | **-2,59** |
| 597 | 456 | **NM_008264.1** | **Hoxa13** | **-2,59** |
| 790 | 664 | **NM_010311.4** | **Gnaz** | **-2,59** |
| 4676 | 4082 | **NM_134109.2** | **Ildr1** | **-2,58** |
| 3205 | 2749 | **NM_001004139.2** | **Zfp619** | **-2,58** |
| 6913 | 5692 | **NM_021422.4** | **Dnaja4** | **-2,58** |
| 1713 | 1501 | **NM_010193.4** | **Fem1b** | **-2,58** |
| 2532 | 2165 | **NM_011211.3** | **Ptprd** | **-2,57** |
| 478 | 405 | **NM_011133.2** | **Pole2** | **-2,57** |
| 1620 | 1371 | **NM_010571.3** | **Irs3** | **-2,57** |
| 10915 | 8867 | **NM_008232.3** | **Hdgfl1** | **-2,56** |
| 704 | 619 | **NM_019740.2** | **Foxo3** | **-2,56** |
| 7831 | 6283 | **NM_013511.3** | **Epb41l2** | **-2,55** |
| 4177 | 3478 | **NM_011203.3** | **Ptpn12** | **-2,55** |
| 14743 | 11414 | **null** | **null** | **-2,55** |
| 648 | 533 | **NM_011098.4** | **Pitx2** | **-2,54** |
| 369 | 252 | **NM_011252.4** | **Rbmx** | **-2,54** |
| 981 | 829 | **NM_139294.5** | **Braf** | **-2,53** |
| 564 | 453 | **NM_027946.3** | **Dcaf7** | **-2,53** |
| 1619 | 1404 | **NM_008575.4** | **Mdm4** | **-2,53** |
| 7507 | 6144 | **NM_133768.5** | **Asl** | **-2,52** |
| 2641 | 2190 | **NM_026459.4** | **Ccdc70** | **-2,52** |
| 8176 | 6801 | **null** | **5430439C14Rik** | **-2,52** |
| 3647 | 3057 | **null** | **2900074G08Rik** | **-2,51** |
| 4047 | 3413 | **NM_013823.2** | **Kl** | **-2,51** |
| 43154 | 33345 | **NM_028874.2** | **Snx19** | **-2,51** |
| 918 | 766 | **NM_011911.1** | **Vmn1r49** | **-2,50** |
| 1685 | 1441 | **null** | **4831407H17Rik** | **-2,50** |
| 11382 | 9030 | **NM_007688.2** | **Cfl2** | **-2,50** |
| 4161 | 3406 | **null** | **2810442N19Rik** | **-2,49** |
| 5193 | 4446 | **NM_032000.2** | **Trps1** | **-2,49** |
| 1197 | 1047 | **NM_010106.2** | **Eef1a1** | **-2,49** |
| 3446 | 3048 | **NM_010701.3** | **Cnmd** | **-2,49** |
| 334 | 235 | **NM_008928.5** | **Map2k3** | **-2,48** |
| 15616 | 12068 | **NM_026830.2** | **Rreb1** | **-2,48** |
| 2645 | 2344 | **NM_023328.3** | **Agtpbp1** | **-2,48** |
| 141 | 43 | **NM_008982.5** | **Ptprj** | **-2,48** |
| 4261 | 3578 | **NM_009246.3** | **Serpina1d** | **-2,47** |
| 373 | 300 | **NM_029998.3** | **6030458C11Rik** | **-2,47** |
| 298 | 195 | **null** | **5730437C11Rik** | **-2,47** |
| 5411 | 4674 | **null** | **4930594C11Rik** | **-2,46** |
| 3617 | 3059 | **NM_175464.2** | **Pkp4** | **-2,46** |
| 753 | 622 | **NM_008813.4** | **Enpp1** | **-2,46** |
| 3010 | 2678 | **NM_027494.3** | **Zcchc8** | **-2,45** |
| 5784 | 4895 | **NM_177816.3** | **Sh2d4b** | **-2,44** |
| 1064 | 906 | **NM_019910.2** | **Dcpp1** | **-2,44** |
| 4039 | 3422 | **NM_145609.2** | **Msantd4** | **-2,44** |
| 2943 | 2538 | **NM_133962.3** | **Arhgef18** | **-2,44** |
| 1881 | 1644 | **NM_011897.3** | **Spry2** | **-2,43** |
| 1184 | 987 | **NM_001081011.2** | **Srgap2** | **-2,43** |
| 27289 | 20664 | **NM_010790.2** | **Melk** | **-2,43** |
| 3518 | 3022 | **NM_007554.3** | **Bmp4** | **-2,43** |
| 5387 | 4479 | **NM_001310536.1** | **Rapgef2** | **-2,43** |
| 3145 | 2628 | **NM_015803.3** | **Atp8a2** | **-2,43** |
| 2894 | 2534 | **NM_008237.4** | **Hes3** | **-2,43** |
| 1387 | 1190 | **NM_197992.1** | **Pcgf1** | **-2,43** |
| 18315 | 13799 | **NM_001276494.1** | **Dag1** | **-2,43** |
| 2247 | 1959 | **null** | **4930572K03Rik** | **-2,43** |
| 2209 | 1921 | **NM_173863.2** | **Crtc3** | **-2,43** |
| 6879 | 5615 | **NM_009268.1** | **Mucl1** | **-2,42** |
| 4711 | 3995 | **null** | **1700074I03Rik** | **-2,42** |
| 5858 | 5119 | **NM_053262.3** | **Hsd17b11** | **-2,42** |
| 2210 | 1938 | **null** | **9430037O13Rik** | **-2,41** |
| 882 | 757 | **null** | **1700016P22Rik** | **-2,41** |
| 11051 | 8596 | **NM_178373.4** | **Cidec** | **-2,41** |
| 29656 | 20879 | **NM_026527.3** | **Chac2** | **-2,41** |
| 3424 | 3024 | **NM_008150.2** | **Gpc4** | **-2,41** |
| 48426 | 37823 | **NM_173390.3** | **Nhsl1** | **-2,40** |
| 3008 | 2595 | **NM_173374.4** | **Srsf1** | **-2,40** |
| 190 | 114 | **NM_025466.1** | **Gkn1** | **-2,40** |
| 1534 | 1321 | **NM_025992.2** | **Herc6** | **-2,39** |
| 416 | 330 | **null** | **1110014L15Rik** | **-2,39** |
| 4982 | 4223 | **NM_011254.5** | **Rbp1** | **-2,38** |
| 5747 | 4758 | **NM_175164.4** | **Arhgap26** | **-2,38** |
| 313 | 224 | **NM_080556.3** | **Tm9sf2** | **-2,38** |
| 3945 | 3295 | **NM_007681.3** | **Cenpa** | **-2,38** |
| 4749 | 3921 | **NM_009017.1** | **Raet1b** | **-2,37** |
| 2514 | 2089 | **NM_010241.5** | **Aktip** | **-2,36** |
| 1340 | 1163 | **NM_026934.3** | **Zc3h15** | **-2,36** |
| 1100 | 935 | **NM_007686.3** | **Cfi** | **-2,36** |
| 5013 | 4381 | **NM_007832.4** | **Dck** | **-2,36** |
| 1571 | 1342 | **NM_008548.4** | **Man1a** | **-2,35** |
| 7069 | 5842 | **NM_029078.3** | **Pcf11** | **-2,35** |
| 14749 | 11130 | **NM_010747.2** | **Lyn** | **-2,34** |
| 361 | 270 | **NM_007557.3** | **Bmp7** | **-2,34** |
| 4123 | 3622 | **NM_027230.5** | **Zmynd8** | **-2,34** |
| 1860 | 1560 | **NM_011291.5** | **Rpl7** | **-2,34** |
| 1679 | 1472 | **null** | **6330563C09Rik** | **-2,34** |
| 5808 | 5071 | **NM_178278.4** | **Caps2** | **-2,34** |
| 1636 | 1408 | **NM_029659.4** | **Styxl1** | **-2,34** |
| 1713 | 1518 | **NM_053122.4** | **Immp2l** | **-2,33** |
| 7430 | 6231 | **null** | **4930588G17Rik** | **-2,33** |
| 8462 | 6846 | **null** | **4933406F09Rik** | **-2,33** |
| 6462 | 5448 | **null** | **null** | **-2,33** |
| 1890 | 1651 | **NM_010755.4** | **Maff** | **-2,32** |
| 4497 | 3923 | **NM_177191.3** | **Sycp2** | **-2,32** |
| 1673 | 1447 | **NM_145483.2** | **Zfp160** | **-2,32** |
| 5055 | 4269 | **NM_023735.2** | **Actr3** | **-2,31** |
| 1109 | 981 | **NM_001163625.1** | **Sult6b1** | **-2,31** |
| 8995 | 7207 | **NM_010171.3** | **F3** | **-2,31** |
| 11368 | 9194 | **NM_011906.2** | **Tpra1** | **-2,30** |
| 1680 | 1491 | **NM_173782.3** | **Gm9725** | **-2,30** |
| 801 | 678 | **null** | **4930563F08Rik** | **-2,30** |
| 6255 | 5322 | **NM_172476.4** | **Tmc7** | **-2,30** |
| 835 | 689 | **NM_021273.4** | **Ckb** | **-2,29** |
| 3055 | 2598 | **NM_007559.5** | **Bmp8b** | **-2,29** |
| 2058 | 1779 | **NM_024215.2** | **Zfp593** | **-2,29** |
| 9431 | 8061 | **null** | **Ctcflos** | **-2,29** |
| 5841 | 5064 | **null** | **Mettl5os** | **-2,29** |
| 7253 | 5884 | **NM_181849.3** | **Fgb** | **-2,28** |
| 3294 | 2811 | **NM_001162923.1** | **Rasl12** | **-2,28** |
| 2957 | 2524 | **NM_019642.4** | **Rpn2** | **-2,28** |
| 430 | 363 | **NM_021305.4** | **Sec61a2** | **-2,28** |
| 2679 | 2369 | **NM_139218.1** | **Dppa3** | **-2,28** |
| 17161 | 13024 | **null** | **9530086P17Rik** | **-2,28** |
| 4421 | 3895 | **NM_019786.4** | **Tbk1** | **-2,27** |
| 450 | 372 | **NM_175472.3** | **Tut4** | **-2,27** |
| 17583 | 14018 | **NM_029508.3** | **Pcgf5** | **-2,27** |
| 13559 | 10953 | **null** | **4930517G19Rik** | **-2,27** |
| 3560 | 3069 | **null** | **4930441J16Rik** | **-2,27** |
| 1873 | 1619 | **NM_001357549.1** | **Kmt2a** | **-2,27** |
| 1959 | 1617 | **NM_009333.4** | **Tcf7l2** | **-2,26** |
| 2851 | 2533 | **null** | **Igh-VJ558** | **-2,26** |
| 2309 | 1996 | **NM_020259.4** | **Hhip** | **-2,26** |
| 10110 | 9039 | **NM_027926.3** | **Cpa4** | **-2,26** |
| 32846 | 24663 | **NM_177765.3** | **Ttll13** | **-2,26** |
| 12528 | 10231 | **NM_008929.3** | **Dnajc3** | **-2,26** |
| 1671 | 1454 | **NM_015767.4** | **Ttpa** | **-2,25** |
| 463 | 391 | **NM_001278671.1** | **Kbtbd12** | **-2,25** |
| 1174 | 1020 | **NM_025763.1** | **4933436I01Rik** | **-2,25** |
| 10091 | 7847 | **NM_025606.3** | **Mrpl16** | **-2,25** |
| 10843 | 8969 | **NM_027860.3** | **0610010F05Rik** | **-2,25** |
| 4347 | 3704 | **NM_009145.2** | **Nptn** | **-2,25** |
| 1267 | 1132 | **null** | **4930562M23Rik** | **-2,25** |
| 2446 | 1973 | **null** | **Igkv4-91** | **-2,24** |
| 14738 | 11611 | **NM_026576.4** | **Etaa1** | **-2,24** |
| 161 | 90 | **null** | **C630043D15Rik** | **-2,24** |
| 622 | 528 | **NM_001360520.1** | **Slc4a7** | **-2,24** |
| 1415 | 1241 | **NM_027442.5** | **Ddo** | **-2,24** |
| 13284 | 10903 | **NM_009990.3** | **Clip2** | **-2,24** |
| 1385 | 1230 | **null** | **Nrg3os** | **-2,24** |
| 1365 | 1183 | **NM_013584.2** | **Lifr** | **-2,24** |
| 1377 | 1210 | **NM_009713.4** | **Arsa** | **-2,24** |
| 935 | 803 | **NM_021898.2** | **Tsga8** | **-2,24** |
| 955 | 826 | **NM_028058.4** | **Fundc1** | **-2,23** |
| 2991 | 2608 | **NM_177868.4** | **Fhad1** | **-2,22** |
| 6832 | 5684 | **NM_011239.2** | **Ranbp1** | **-2,22** |
| 8285 | 6491 | **null** | **Igh-V7183** | **-2,22** |
| 8508 | 6955 | **NM_001163793.1** | **C530008M17Rik** | **-2,22** |
| 8093 | 6876 | **NM_019398.2** | **Rnase2b** | **-2,21** |
| 5138 | 4385 | **null** | **2900097C17Rik** | **-2,21** |
| 1436 | 1263 | **NM_010072.4** | **Dpm1** | **-2,21** |
| 2998 | 2670 | **NM_146386.3** | **Myocd** | **-2,21** |
| 7643 | 6398 | **NM_030026.2** | **Mccc2** | **-2,21** |
| 1717 | 1529 | **NM_009322.3** | **Tbr1** | **-2,21** |
| 1484 | 1291 | **NM_026789.4** | **Cfap57** | **-2,21** |
| 3750 | 3234 | **null** | **Mszf17** | **-2,21** |
| 1625 | 1415 | **NM_145852.2** | **Ropn1l** | **-2,20** |
| 8610 | 6958 | **NM_030690.3** | **Rai14** | **-2,20** |
| 2488 | 2162 | **NM_133203.5** | **Klra17** | **-2,20** |
| 831 | 756 | **NM_145956.4** | **Brcc3** | **-2,20** |
| 10522 | 8392 | **null** | **1700014D06Rik** | **-2,20** |
| 1302 | 1152 | **NM_011859.3** | **Osr1** | **-2,20** |
| 4727 | 3973 | **NM_028169.1** | **Fam71e1** | **-2,20** |
| 1290 | 1144 | **NM_008768.2** | **Orm1** | **-2,20** |
| 3749 | 3328 | **NM_025299.3** | **Txnl4a** | **-2,19** |
| 21526 | 16860 | **NM_025287.2** | **Spop** | **-2,19** |
| 2048 | 1851 | **NM_001003918.2** | **Usp7** | **-2,19** |
| 311 | 225 | **NM_027106.4** | **Avpi1** | **-2,19** |
| 1375 | 1228 | **null** | **5430425E15Rik** | **-2,19** |
| 7742 | 6458 | **NM_009084.4** | **Rpl37a** | **-2,18** |
| 2623 | 2262 | **NM_009592.1** | **Abcb7** | **-2,18** |
| 2497 | 2185 | **NM_028108.3** | **Naa50** | **-2,18** |
| 5690 | 4878 | **NM_007422.3** | **Adss** | **-2,18** |
| 29512 | 22566 | **NM_011283.2** | **Rp1** | **-2,18** |
| 8237 | 6541 | **null** | **4933427I22Rik** | **-2,18** |
| 26221 | 21001 | **NM_145537.2** | **Edem2** | **-2,18** |
| 3467 | 3050 | **NM_026982.1** | **Tmem256** | **-2,18** |
| 5188 | 4584 | **null** | **4833418N17Rik** | **-2,18** |
| 6605 | 5666 | **NM_019743.3** | **Rybp** | **-2,18** |
| 3398 | 2986 | **NM_009403.3** | **Tnfsf8** | **-2,17** |
| 5135 | 4617 | **NM_029851.3** | **Dync2h1** | **-2,17** |
| 1929 | 1637 | **NM_026508.2** | **Trap1** | **-2,17** |
| 2560 | 2209 | **NM_008141.3** | **Gnat2** | **-2,17** |
| 3044 | 2665 | **NM_134137.3** | **Lars** | **-2,16** |
| 2726 | 2409 | **NM_145121.3** | **Cacnb1** | **-2,16** |
| 983 | 847 | **NM_028511.1** | **Cldn34b2** | **-2,16** |
| 2235 | 1970 | **NM_008182.3** | **Gsta2** | **-2,16** |
| 4676 | 4103 | **NM_009338.3** | **Acat2** | **-2,16** |
| 22050 | 17154 | **NM_133656.5** | **Crk** | **-2,16** |
| 5069 | 4391 | **NM_026380.3** | **Rgs8** | **-2,15** |
| 2067 | 1814 | **NM_009806.3** | **Cask** | **-2,15** |
| 1039 | 892 | **NM_011668.3** | **Ube3a** | **-2,15** |
| 1342 | 1185 | **NM_023045.3** | **Xpo7** | **-2,15** |
| 1764 | 1610 | **NM_025814.2** | **Serbp1** | **-2,14** |
| 251 | 156 | **NM_029949.4** | **Snapc3** | **-2,14** |
| 704 | 633 | **NM_026147.6** | **Rps20** | **-2,14** |
| 3737 | 3140 | **null** | **1300002E11Rik** | **-2,14** |
| 4120 | 3526 | **NM_007853.5** | **Degs1** | **-2,14** |
| 1490 | 1303 | **NM_028708.2** | **Jakmip3** | **-2,14** |
| 1074 | 944 | **NM_152812.4** | **Otud6b** | **-2,14** |
| 1481 | 1340 | **NM_009521.2** | **Wnt3** | **-2,14** |
| 1352 | 1190 | **NM_001081137.2** | **Sis** | **-2,13** |
| 2802 | 2486 | **NM_031159.3** | **Apobec1** | **-2,13** |
| 2283 | 2069 | **NM_001081290.1** | **Prrc2c** | **-2,13** |
| 22120 | 15133 | **NM_177323.4** | **Rint1** | **-2,13** |
| 64 | 26 | **NM_021368.1** | **Olfr1264** | **-2,13** |
| 1854 | 1625 | **NM_013454.3** | **Abca1** | **-2,12** |
| 1738 | 1582 | **NM_010143.1** | **Ephb3** | **-2,12** |
| 1733 | 1567 | **NM_008205.1** | **H2-M9** | **-2,12** |
| 3580 | 3197 | **NM_009076.3** | **Rpl12** | **-2,12** |
| 5218 | 4598 | **NM_019659.3** | **Kcnj1** | **-2,12** |
| 1429 | 1263 | **NM_001005510.2** | **Syne2** | **-2,11** |
| 6021 | 5022 | **NM_011854.2** | **Oasl2** | **-2,11** |
| 5994 | 5276 | **NM_007947.3** | **Lcn5** | **-2,11** |
| 80 | 32 | **NM_011904.3** | **Tll2** | **-2,11** |
| 1570 | 1384 | **NM_054083.2** | **Stim2** | **-2,11** |
| 3560 | 3181 | **NM_153406.3** | **Specc1l** | **-2,10** |
| 1704 | 1535 | **NM_030248.2** | **Cdk5rap3** | **-2,10** |
| 2488 | 2157 | **NM_031193.2** | **Ren2** | **-2,10** |
| 6940 | 5875 | **NM_008165.4** |  | **-2,10** |
| 1494 | 1332 | **NM_022979.2** | **Nup98** | **-2,10** |
| 4467 | 3769 | **NM_021389.6** | **Sh3kbp1** | **-2,10** |
| 3078 | 2730 | **NM_178447.3** | **Zfp2** | **-2,10** |
| 1937 | 1634 | **NM_183293.1** | **Ccdc83** | **-2,10** |
| 5369 | 4709 | **NM_144517.4** | **Tbc1d19** | **-2,09** |
| 12875 | 10449 | **NM_054063.4** | **Psg28** | **-2,09** |
| 572 | 511 | **NM_010807.4** | **Marcksl1** | **-2,09** |
| 3041 | 2601 | **NM_175446.3** | **Zmat1** | **-2,09** |
| 904 | 776 | **NM_183123.2** | **Spinkl** | **-2,09** |
| 2928 | 2615 | **NM_010064.4** | **Dync1i2** | **-2,09** |
| 73 | 31 | **NM_013457.4** | **Add1** | **-2,09** |
| 21336 | 16208 | **NM_011426.3** | **Siglec1** | **-2,09** |
| 2095 | 1777 | **NM_001081086.1** | **Ppig** | **-2,08** |
| 820 | 723 | **NM_009789.2** | **S100g** | **-2,08** |
| 4723 | 4160 | **NM_008989.4** | **Pura** | **-2,08** |
| 7154 | 6044 | **NM_013603.2** | **Mt3** | **-2,08** |
| 1519 | 1335 | **NM_013563.4** | **Il2rg** | **-2,08** |
| 1949 | 1721 | **NM_134071.3** | **Slf1** | **-2,08** |
| 5141 | 4615 | **NM_146235.3** | **Ercc6l** | **-2,07** |
| 3254 | 2860 | **NM_033623.6** | **Dcun1d1** | **-2,07** |
| 3904 | 3348 | **NM_028027.3** | **Arhgef25** | **-2,07** |
| 10770 | 8937 | **NM_133728.3** | **Asnsd1** | **-2,07** |
| 1631 | 1445 | **null** | **1700066D14Rik** | **-2,07** |
| 4568 | 3891 | **NM_009915.2** | **Ccr2** | **-2,07** |
| 21147 | 17099 | **NM_011265.3** | **Rfx3** | **-2,06** |
| 1906 | 1677 | **NM_008685.3** | **Nfe2** | **-2,06** |
| 942 | 848 | **NM_146232.1** | **Slc22a26** | **-2,06** |
| 16404 | 12643 | **NM_027840.3** | **Snx20** | **-2,06** |
| 4916 | 4260 | **NM_025952.4** | **Magt1** | **-2,06** |
| 1618 | 1449 | **NM_027469.4** | **Gfod2** | **-2,05** |
| 695 | 628 | **NM_172338.2** | **Dnajc16** | **-2,05** |
| 218 | 142 | **NM_173750.2** | **Arl14ep** | **-2,05** |
| 2138 | 1886 | **null** | **9530025H10Rik** | **-2,05** |
| 13037 | 10928 | **NM_145357.1** | **BC023105** | **-2,05** |
| 301 | 209 | **null** | **4930507D10Rik** | **-2,04** |
| 4298 | 3822 | **NM_027184.2** | **Ipmk** | **-2,04** |
| 19504 | 15570 | **NM_144513.2** | **Meg3** | **-2,04** |
| 5129 | 4500 | **NM_010477.4** | **Hspd1** | **-2,04** |
| 5512 | 4681 | **NM_026486.3** | **Tctn2** | **-2,04** |
| 94 | 35 | **NM_010080.3** | **Dspp** | **-2,04** |
| 8007 | 6929 | **NM_033509.4** | **Vangl2** | **-2,04** |
| 23469 | 19413 | **NM_010549.3** | **Il11ra1** | **-2,04** |
| 5670 | 4863 | **NM_011636.2** | **Plscr1** | **-2,04** |
| 11619 | 10188 | **null** | **4921508D12Rik** | **-2,04** |
| 5102 | 4422 | **null** | **4933425B07Rik** | **-2,03** |
| 63 | 27 | **NM_029086.2** | **Trmo** | **-2,03** |
| 4375 | 3931 | **null** | **1810073O08Rik** | **-2,03** |
| 1861 | 1648 | **NM_001254953.1** | **Ankrd66** | **-2,03** |
| 3136 | 2786 | **NM_027374.3** | **Ppil3** | **-2,03** |
| 402 | 333 | **NM_013673.4** | **Sp100** | **-2,03** |
| 1656 | 1484 | **null** | **Igh-VS107** | **-2,03** |
| 12392 | 10482 | **NM_013862.5** | **Rabgap1l** | **-2,03** |
| 1203 | 1089 | **NM_010806.1** | **Afdn** | **-2,03** |
| 1611 | 1440 | **NM_029163.3** | **Usp50** | **-2,03** |
| 739 | 635 | **NM_010750.3** | **Mab21l1** | **-2,02** |
| 2613 | 2180 | **NM_053106.2** | **Lmod1** | **-2,02** |
| 1069 | 932 | **NM_028377.3** | **Bbof1** | **-2,02** |
| 3217 | 2810 | **NM_027782.3** | **Kctd6** | **-2,02** |
| 2750 | 2491 | **NM_146231.1** | **Zfp825** | **-2,02** |
| 7828 | 6973 | **NM_026330.3** | **Nsmce1** | **-2,01** |
| 10249 | 9013 | **NM_172134.2** | **Pdxk** | **-2,01** |
| 9197 | 7958 | **NM_010518.2** | **Igfbp5** | **-2,01** |
| 1847 | 1622 | **null** | **1700003I22Rik** | **-2,01** |
| 4100 | 3539 | **NM_029936.2** | **Ddx10** | **-2,01** |
| 9002 | 7414 | **NM_007708.3** | **Cit** | **-2,00** |
| 4955 | 4284 | **NM_020583.5** | **Isg20** | **-2,00** |
| 36040 | 29938 | **NM_172544.3** | **Nrxn3** | **-2,00** |
| 2457 | 2094 | **NM_007854.3** | **Slc29a2** | **-2,00** |
| 3133 | 2787 | **NM_033604.3** | **Rnf111** | **-2,00** |
| 11210 | 9588 | **NM_019468.2** | **G6pd2** | **-2,00** |
| 4893 | 4264 | **null** | **1700042D02Rik** | **-2,00** |
| 5702 | 5000 | **NM_001289926.1** | **2010111I01Rik** | **-2,00** |
| 1456 | 1281 | **NM_013552.2** | **Hmmr** | **-2,00** |

| 9 months – mRNA up in SAMP8 (346) | | | | |
| --- | --- | --- | --- | --- |
| Cy3 | **Cy5** | **Accesion number** | **Gene Symbol** | **Zscore** |
| 29 | 87 | **NM_001163635.1** | **Tnks2** | **4,27** |
| 153 | 536 | **NM_027832.3** | **Sval1** | **4,21** |
| 51 | 243 | **NM_027185.3** | **Def6** | **4,19** |
| 79 | 253 | **NM_009514.5** | **Vpreb3** | **4,03** |
| 110 | 310 | **NM_022317.3** | **Slc28a3** | **3,93** |
| 45 | 117 | **NM_010676.2** | **Krtap19-5** | **3,92** |
| 5397 | 30121 | **null** | **null** | **3,83** |
| 47 | 126 | **null** | **Olfr29-ps1** | **3,81** |
| 49 | 95 | **NM_130890.2** | **Capn8** | **3,75** |
| 63 | 155 | **NM_028479.2** | **Mrgbp** | **3,75** |
| 138 | 350 | **NM_007602.4** | **Capn5** | **3,62** |
| 128 | 303 | **NM_146178.2** | **Ccdc106** | **3,43** |
| 266 | 815 | **NM_023785.3** | **Ppbp** | **3,36** |
| 607 | 2313 | **NM_026678.4** | **Blvra** | **3,31** |
| 750 | 3074 | **NM_025639.4** | **Cenpm** | **3,31** |
| 8011 | 25634 | **NM_016867.1** | **Gipc2** | **3,27** |
| 212 | 589 | **null** | **Igkv9-120** | **3,24** |
| 142 | 362 | **NM_019680.2** | **Elf4** | **3,24** |
| 21 | 57 | **NM_023059.3** | **Sigirr** | **3,22** |
| 141 | 316 | **null** | **4930558C23Rik** | **3,15** |
| 432 | 1341 | **NM_025960.4** | **Trappc6a** | **3,10** |
| 1732 | 8018 | **null** | **D330022H12Rik** | **3,06** |
| 53 | 104 | **NM_025304.3** | **Lcmt1** | **3,04** |
| 23 | 63 | **NM_013473.4** | **Anxa8** | **3,02** |
| 331 | 769 | **NM_001355549.1** | **Ccdc171** | **3,01** |
| 67 | 130 | **NM_133985.2** | **Oxsr1** | **3,01** |
| 109 | 215 | **NM_010010.2** | **Cyp46a1** | **2,99** |
| 22 | 50 | **null** | **4933407C09Rik** | **2,98** |
| 1432 | 4346 | **NM_028175.2** | **Lrrc8e** | **2,97** |
| 132 | 320 | **NM_001040699.1** | **Mtmr7** | **2,97** |
| 160 | 325 | **NM_011508.2** | **Eif1** | **2,97** |
| 924 | 2321 | **NM_027276.3** | **Cdc16** | **2,96** |
| 51 | 110 | **NM_008865.3** | **Prl3b1** | **2,96** |
| 38 | 73 | **NM_178212.3** | **Hist2h2aa2** | **2,94** |
| 68 | 132 | **NM_008583.2** | **Men1** | **2,93** |
| 44 | 89 | **NM_145933.4** | **St6gal1** | **2,93** |
| 14 | 53 | **NM_027948.1** | **1700003E16Rik** | **2,93** |
| 30 | 71 | **NM_011706.2** | **Trpv2** | **2,91** |
| 214 | 520 | **NM_010919.2** | **Nkx2-2** | **2,90** |
| 63 | 125 | **NM_010722.5** | **Lmnb2** | **2,88** |
| 263 | 619 | **null** | **Adh6-ps1** | **2,88** |
| 117 | 242 | **NM_030208.3** | **Trmt44** | **2,83** |
| 37 | 78 | **NM_013835.2** | **Trove2** | **2,83** |
| 271 | 595 | **null** | **5330417P21Rik** | **2,82** |
| 199 | 368 | **NM_017397.3** | **Ddx20** | **2,81** |
| 90 | 176 | **NM_172426.2** | **Slc24a2** | **2,81** |
| 993 | 2213 | **NM_146124.4** | **Arhgap1** | **2,81** |
| 77 | 148 | **NM_011875.4** | **Psmd13** | **2,81** |
| 312 | 759 | **NM_153410.5** | **Gpsm1** | **2,81** |
| 1143 | 3202 | **null** | **4930439D14Rik** | **2,80** |
| 196 | 344 | **null** | **Igh-V15** | **2,79** |
| 24 | 52 | **null** | **4921528I07Rik** | **2,78** |
| 220 | 435 | **NM_013697.5** | **Ttr** | **2,73** |
| 79 | 168 | **NM_011750.2** | **Sf1** | **2,73** |
| 50 | 92 | **NM_146126.4** | **Sord** | **2,72** |
| 140 | 286 | **NM_019566.3** | **Rhog** | **2,72** |
| 39 | 72 | **NM_145920.3** | **Evc2** | **2,70** |
| 85 | 153 | **NM_011044.3** | **Pck1** | **2,70** |
| 53 | 98 | **NM_030706.3** | **Trim2** | **2,69** |
| 78 | 133 | **NM_013924.3** | **Abt1** | **2,67** |
| 18 | 48 | **NM_009733.2** | **Axin1** | **2,67** |
| 33 | 67 | **NM_176942.4** | **Gabra5** | **2,67** |
| 241 | 575 | **NM_009670.4** | **Ank3** | **2,67** |
| 20 | 47 | **NM_011992.2** | **Rcn2** | **2,66** |
| 51 | 95 | **NM_013891.4** | **Spdef** | **2,64** |
| 39 | 84 | **NM_007751.3** | **Cox8b** | **2,63** |
| 498 | 1413 | **null** | **4633401L03Rik** | **2,63** |
| 303 | 634 | **NM_008540.3** | **Smad4** | **2,63** |
| 144 | 248 | **null** | **4930591E09Rik** | **2,62** |
| 140 | 270 | **NM_029623.2** | **Rmc1** | **2,61** |
| 22 | 53 | **NM_009605.5** | **Adipoq** | **2,61** |
| 34 | 67 | **NM_054040.3** | **Tulp4** | **2,61** |
| 53 | 89 | **NM_008598.2** | **Mgmt** | **2,61** |
| 252 | 463 | **null** | **Rsph10b** | **2,61** |
| 147 | 255 | **NM_133208.2** | **Zfp287** | **2,61** |
| 7897 | 17592 | **NM_207203.2** | **Prr36** | **2,61** |
| 240 | 511 | **null** | **3110015C05Rik** | **2,60** |
| 253 | 540 | **NM_025626.4** | **Fam107b** | **2,59** |
| 25 | 54 | **NM_018784.3** | **St3gal6** | **2,58** |
| 16732 | 37434 | **NM_001081406.2** | **Lrr1** | **2,58** |
| 211 | 450 | **NM_145130.2** | **Lpcat3** | **2,57** |
| 20 | 49 | **NM_008133.4** | **Glud1** | **2,56** |
| 42 | 87 | **NM_011278.5** | **Rnf4** | **2,56** |
| 50 | 81 | **NM_019643.4** | **Sinhcaf** | **2,56** |
| 61 | 100 | **null** | **4930535B17Rik** | **2,56** |
| 68 | 112 | **NM_199312.4** | **Fndc11** | **2,55** |
| 68 | 180 | **NM_029492.5** | **Zdhhc20** | **2,55** |
| 22 | 58 | **NM_007649.5** | **Cd48** | **2,55** |
| 827 | 2152 | **NM_028093.1** | **Entpd8** | **2,54** |
| 28 | 56 | **NM_001033181.1** | **Jrkl** | **2,54** |
| 32 | 64 | **NM_030720.1** | **Gpr84** | **2,53** |
| 34 | 64 | **NM_181277.3** | **Col14a1** | **2,52** |
| 320 | 617 | **null** | **Snora15** | **2,51** |
| 2814 | 7417 | **NM_029791.4** | **Bicd2** | **2,51** |
| 47 | 90 | **NM_008323.1** | **Idh3g** | **2,50** |
| 34 | 64 | **NM_010886.3** | **Ndufa4** | **2,49** |
| 225 | 494 | **NM_008334.3** | **Ifna7** | **2,48** |
| 17 | 46 | **NM_011359.2** | **Sftpc** | **2,48** |
| 13 | 39 | **NM_015828.3** | **Gne** | **2,47** |
| 358 | 675 | **NM_011111.4** | **Serpinb2** | **2,47** |
| 51 | 98 | **NM_013638.2** | **Prm3** | **2,46** |
| 44 | 78 | **NM_023628.2** | **Anxa9** | **2,46** |
| 151 | 335 | **null** | **4833422M21Rik** | **2,45** |
| 37 | 73 | **NM_007640.2** | **Cd1d2** | **2,45** |
| 59 | 113 | **NM_011780.3** | **Adam23** | **2,45** |
| 203 | 378 | **NM_029842.5** | **Kdm8** | **2,44** |
| 86 | 135 | **NM_011261.2** | **Reln** | **2,44** |
| 123 | 209 | **NM_197979.3** | **Uqcr10** | **2,43** |
| 33 | 70 | **NM_019587.2** | **Plxnb3** | **2,43** |
| 79 | 142 | **NM_008590.2** | **Mest** | **2,43** |
| 104 | 183 | **NM_028544.1** | **Rasip1** | **2,43** |
| 21618 | 46037 | **NM_023815.4** | **Trp53rkb** | **2,42** |
| 222 | 378 | **NM_011244.4** | **Rarg** | **2,42** |
| 431 | 1120 | **null** | **5830462O15Rik** | **2,42** |
| 132 | 252 | **NM_172291.2** | **Foxred1** | **2,41** |
| 133 | 236 | **NM_028814.4** | **Khdc4** | **2,40** |
| 139 | 237 | **NM_148917.3** | **Pabpc4** | **2,39** |
| 41 | 80 | **NM_029963.2** | **Mrps5** | **2,39** |
| 379 | 926 | **NM_025852.3** | **Rexo1** | **2,39** |
| 156 | 282 | **NM_027442.5** | **Ddo** | **2,39** |
| 173 | 312 | **null** | **Snord16a** | **2,38** |
| 11869 | 26174 | **NM_080708.1** | **Bmp2k** | **2,38** |
| 43 | 77 | **NM_029782.3** | **Calr3** | **2,38** |
| 172 | 316 | **NM_007484.2** | **Rhoc** | **2,37** |
| 11789 | 44796 | **NM_027925.3** | **Trnau1ap** | **2,37** |
| 2188 | 6526 | **null** | **4930555K05Rik** | **2,36** |
| 7686 | 16771 | **null** | **4930456K20Rik** | **2,36** |
| 12586 | 33300 | **null** | **1700128I11Rik** | **2,36** |
| 830 | 2209 | **NM_181316.4** | **Bbs9** | **2,35** |
| 23 | 49 | **NM_007872.4** | **Dnmt3a** | **2,35** |
| 45 | 86 | **NM_138666.4** | **Nlgn1** | **2,35** |
| 66 | 104 | **NM_028622.2** | **Lce1c** | **2,35** |
| 8421 | 16708 | **NM_001163728.1** | **4930563E22Rik** | **2,35** |
| 245 | 490 | **NM_010364.4** | **Gtf2h4** | **2,35** |
| 9350 | 20750 | **NM_133992.3** | **Pan2** | **2,33** |
| 31 | 55 | **NM_011879.2** | **Ik** | **2,33** |
| 396 | 767 | **NM_019912.2** | **Ube2d2a** | **2,33** |
| 84 | 151 | **null** | **1700012E03Rik** | **2,33** |
| 619 | 1585 | **null** | **6330563C09Rik** | **2,32** |
| 26 | 54 | **NM_015744.4** | **Enpp2** | **2,32** |
| 4300 | 12281 | **NM_007505.2** | **Atp5a1** | **2,32** |
| 51 | 82 | **NM_008413.3** | **Jak2** | **2,32** |
| 82 | 127 | **NM_172146.2** | **Ppat** | **2,32** |
| 26 | 51 | **NM_001039544.2** | **Mup3** | **2,32** |
| 124 | 202 | **NM_008637.2** | **Nudt1** | **2,32** |
| 103 | 177 | **null** | **1110013H19Rik** | **2,31** |
| 44 | 82 | **NM_024219.1** | **Hsbp1** | **2,31** |
| 53 | 108 | **NM_023167.2** | **Mrpl4** | **2,31** |
| 51 | 82 | **NM_175155.4** | **Sash1** | **2,30** |
| 482 | 948 | **NM_028751.3** | **Tjap1** | **2,30** |
| 60 | 104 | **NM_013558.2** | **Hspa1l** | **2,30** |
| 2412 | 6194 | **NM_007478.3** | **Arf3** | **2,30** |
| 53 | 92 | **NM_017479.3** | **Kat6b** | **2,30** |
| 118 | 205 | **NM_029875.2** | **Slc35e3** | **2,30** |
| 29 | 54 | **NM_027097.1** | **Klk12** | **2,29** |
| 56 | 97 | **NM_027815.4** | **Vps35l** | **2,29** |
| 70 | 111 | **NM_009070.2** | **Rnps1** | **2,28** |
| 390 | 893 | **NM_054040.3** | **Tulp4** | **2,28** |
| 62 | 105 | **null** | **4933416M06Rik** | **2,28** |
| 69 | 128 | **null** | **C330002G04Rik** | **2,28** |
| 57 | 90 | **NM_010920.2** | **Nkx2-6** | **2,27** |
| 7222 | 16212 | **NM_175105.3** | **Aqp11** | **2,27** |
| 15 | 38 | **NM_009292.2** | **Stra8** | **2,27** |
| 56 | 107 | **NM_009648.2** | **Akap1** | **2,27** |
| 47 | 88 | **NM_008161.4** | **Gpx3** | **2,27** |
| 38 | 65 | **NM_199042.2** | **Thap1** | **2,27** |
| 109 | 211 | **NM_029545.3** | **Dtd2** | **2,27** |
| 44 | 87 | **NM_021560.4** | **Bhlhe22** | **2,26** |
| 82 | 153 | **null** | **8030448I15Rik** | **2,26** |
| 3008 | 7853 | **NM_008218.2** | **Hba-a1** | **2,26** |
| 776 | 1506 | **NM_013750.2** | **Phlda3** | **2,26** |
| 33 | 54 | **NM_021288.4** | **Tyms** | **2,25** |
| 13967 | 28804 | **NM_013647.2** | **Rps16** | **2,25** |
| 249 | 562 | **NM_021337.2** | **Skiv2l** | **2,25** |
| 249 | 430 | **NM_029771.3** | **Gper1** | **2,25** |
| 7831 | 21454 | **null** | **5930409G06Rik** | **2,25** |
| 65 | 106 | **NM_028148.2** | **Scaf11** | **2,24** |
| 93 | 161 | **null** | **null** | **2,24** |
| 69 | 110 | **NM_026831.1** | **Mybphl** | **2,24** |
| 114 | 189 | **NM_008361.4** | **Il1b** | **2,24** |
| 73 | 105 | **null** | **4930480C01Rik** | **2,23** |
| 91 | 150 | **NM_007655.4** | **Cd79a** | **2,23** |
| 10408 | 24518 | **NM_175103.3** | **Bola2** | **2,23** |
| 287 | 565 | **NM_011725.4** | **Xlr** | **2,23** |
| 1792 | 4045 | **NM_011584.4** | **Nr1d2** | **2,23** |
| 11170 | 41063 | **NM_012021.3** | **Prdx5** | **2,23** |
| 118 | 176 | **NM_175185.4** | **Hsdl1** | **2,23** |
| 26 | 48 | **NM_175675.3** | **Slc35f6** | **2,23** |
| 19934 | 39903 | **NM_025551.4** | **Ndufa12** | **2,23** |
| 174 | 310 | **null** | **Pard3bos1** | **2,22** |
| 26 | 52 | **NM_009313.5** | **Tacr1** | **2,22** |
| 951 | 2191 | **NM_007561.4** | **Bmpr2** | **2,22** |
| 59 | 98 | **NM_001358562.1** | **Ccdc162** | **2,22** |
| 126 | 208 | **NM_009329.4** | **Zfp354a** | **2,22** |
| 27 | 54 | **NM_007479.4** | **Arf4** | **2,21** |
| 160 | 310 | **NM_175130.4** | **Trpm4** | **2,21** |
| 1159 | 3520 | **NM_023284.3** | **Nuf2** | **2,20** |
| 1629 | 4114 | **NM_028473.2** | **Snorc** | **2,20** |
| 92 | 159 | **NM_009618.3** | **Adam2** | **2,20** |
| 63 | 97 | **NM_001195633.2** | **Epg5** | **2,19** |
| 206 | 336 | **NM_133828.2** | **Creb1** | **2,19** |
| 7814 | 16160 | **NM_007517.4** | **Aup1** | **2,19** |
| 116 | 180 | **NM_152220.2** | **Stx3** | **2,19** |
| 195 | 328 | **NM_009407.2** | **Tnp1** | **2,19** |
| 69 | 120 | **NM_009712.3** | **Arsb** | **2,18** |
| 382 | 816 | **NM_021514.4** | **Pfkm** | **2,18** |
| 78 | 143 | **NM_009591.3** | **Aanat** | **2,18** |
| 89 | 163 | **null** | **4930455M05Rik** | **2,18** |
| 130 | 199 | **null** | **2810452K05Rik** | **2,18** |
| 107 | 177 | **NM_010885.5** | **Ndufa2** | **2,18** |
| 27 | 54 | **NM_054070.3** | **Afg3l1** | **2,17** |
| 106 | 181 | **NM_029336.1** | **Spata45** | **2,17** |
| 55 | 81 | **null** | **4933423N12Rik** | **2,17** |
| 38 | 70 | **null** | **4632404H12Rik** | **2,17** |
| 40 | 69 | **NM_133188.2** | **Dazap1** | **2,16** |
| 45 | 76 | **null** | **4930432N10Rik** | **2,16** |
| 122 | 200 | **NM_053182.5** | **Pag1** | **2,16** |
| 224 | 461 | **NM_028320.4** | **Adipor1** | **2,15** |
| 172 | 269 | **NM_016906.4** | **Sec61a1** | **2,15** |
| 84 | 144 | **NM_027135.2** | **Sec24d** | **2,15** |
| 285 | 443 | **NM_030235.1** | **Avl9** | **2,15** |
| 44 | 79 | **NM_028758.2** | **Gga2** | **2,14** |
| 22 | 47 | **NM_010681.4** | **Lama4** | **2,14** |
| 751 | 1828 | **NM_207110.1** | **Rnf216** | **2,13** |
| 904 | 1831 | **NM_009636.3** | **Aebp1** | **2,12** |
| 119 | 201 | **null** | **4930557F10Rik** | **2,12** |
| 27 | 51 | **NM_021408.3** | **Ush2a** | **2,12** |
| 466 | 955 | **NM_026163.2** | **Pkp2** | **2,12** |
| 56 | 94 | **NM_025698.1** | **Tmed7** | **2,12** |
| 264 | 439 | **NM_021390.3** | **Sall1** | **2,11** |
| 58 | 95 | **NM_183098.1** | **1700084P21Rik** | **2,11** |
| 71 | 118 | **null** | **Copg2os2** | **2,11** |
| 202 | 337 | **NM_011888.2** | **Ccl19** | **2,11** |
| 69 | 105 | **null** | **1700057A11Rik** | **2,11** |
| 41 | 75 | **NM_020623.2** | **Pth** | **2,10** |
| 32 | 55 | **NM_010774.2** | **Mbd4** | **2,10** |
| 149 | 254 | **NM_021521.2** | **Med12** | **2,10** |
| 212 | 401 | **null** | **2810411K19Rik** | **2,09** |
| 2399 | 5234 | **NM_026820.3** | **Ifitm1** | **2,09** |
| 85 | 149 | **NM_028444.1** | **Cavin3** | **2,09** |
| 86 | 132 | **NM_029158.2** | **Zcchc13** | **2,08** |
| 47 | 88 | **null** | **4921517O11Rik** | **2,08** |
| 1116 | 2382 | **NM_177077.2** | **Exoc6b** | **2,08** |
| 74 | 125 | **NM_008550.2** | **Man2b2** | **2,08** |
| 97 | 157 | **NM_028973.2** | **Lrrc15** | **2,08** |
| 765 | 1730 | **NM_008169.3** | **Grin1** | **2,08** |
| 24 | 47 | **NM_011021.3** | **Otp** | **2,07** |
| 7374 | 15267 | **NM_008440.4** | **Kif1a** | **2,07** |
| 781 | 1480 | **NM_172308.4** | **Mthfd1l** | **2,07** |
| 49 | 87 | **NM_198415.3** | **Ckmt2** | **2,07** |
| 33 | 53 | **NM_009390.3** | **Tll1** | **2,06** |
| 7291 | 15850 | **NM_019677.2** | **Plcb1** | **2,06** |
| 24 | 48 | **NM_007482.3** | **Arg1** | **2,06** |
| 190 | 313 | **NM_011258.2** | **Rfc1** | **2,06** |
| 1848 | 5016 | **null** | **4933424G05Rik** | **2,06** |
| 195 | 326 | **null** | **4930467K11Rik** | **2,06** |
| 99 | 164 | **NM_008679.3** | **Ncoa3** | **2,06** |
| 96 | 163 | **NM_008092.4** | **Gata4** | **2,06** |
| 46 | 84 | **NM_011091.1** | **Pira4** | **2,06** |
| 309 | 612 | **NM_027946.3** | **Dcaf7** | **2,05** |
| 7465 | 15185 | **NM_001033141.1** | **Ecscr** | **2,05** |
| 342 | 623 | **NM_021349.2** | **Tnfrsf13b** | **2,05** |
| 101 | 159 | **NM_176833.4** | **Ppm1f** | **2,05** |
| 17 | 35 | **NM_013813.2** | **Epb41l3** | **2,05** |
| 14 | 34 | **NM_026993.3** | **Ddah1** | **2,04** |
| 17 | 44 | **NM_010656.3** | **Sspn** | **2,04** |
| 328 | 548 | **NM_023211.5** | **Usmg5** | **2,04** |
| 36 | 67 | **NM_028688.1** | **1700029M20Rik** | **2,04** |
| 36 | 67 | **NM_024427.4** | **Tpm1** | **2,04** |
| 5453 | 13248 | **NM_029357.3** | **Pcdh1** | **2,03** |
| 120 | 197 | **NM_134131.2** | **Tnfaip8** | **2,03** |
| 1135 | 2112 | **NM_027036.3** | **Hmgb4** | **2,03** |
| 42 | 66 | **null** | **Zcwpw2** | **2,03** |
| 36 | 67 | **NM_013820.3** | **Hk2** | **2,03** |
| 145 | 242 | **NM_019661.4** | **Ykt6** | **2,03** |
| 331 | 603 | **NM_026618.2** | **Coa3** | **2,03** |
| 63 | 93 | **NM_011300.3** | **Rps7** | **2,03** |
| 52 | 87 | **NM_009285.3** | **Stc1** | **2,03** |
| 22 | 48 | **NM_029286.2** | **Ccdc30** | **2,03** |
| 256 | 478 | **NM_144519.4** | **Zfp639** | **2,03** |
| 56 | 85 | **NM_175224.4** | **Metap1** | **2,02** |
| 43 | 68 | **NM_130452.1** | **Bbox1** | **2,02** |
| 2749 | 5823 | **NM_026259.3** | **Rnf41** | **2,02** |
| 63 | 98 | **NM_013901.2** | **Slc39a1** | **2,02** |
| 28 | 51 | **NM_011983.2** | **Homer2** | **2,02** |
| 96 | 156 | **NM_010896.2** | **Neurog1** | **2,02** |
| 273 | 416 | **NM_030702.4** | **Senp3** | **2,01** |
| 134 | 201 | **NM_011485.5** | **Star** | **2,01** |
| 384 | 731 | **NM_011759.3** | **Zfp41** | **2,01** |
| 51 | 89 | **NM_030708.2** | **Zfhx4** | **2,01** |
| 606 | 1192 | **null** | **4930425F17Rik** | **2,00** |
| 45 | 79 | **NM_028119.5** | **Ddb2** | **2,00** |
| 55 | 88 | **NM_023483.4** | **1110032A03Rik** | **2,00** |
| 1197 | 2490 | **null** | **5830461L22Rik** | **2,00** |
| 45 | 81 | **NM_009530.2** | **Atrx** | **2,00** |
| 14 | 33 | **NM_026028.5** | **Ccdc77** | **2,00** |

| 9 months – mRNA down in SAMP8 (687) | | | | |
| --- | --- | --- | --- | --- |
| Cy3 | **Cy5** | **Accesion number** | **Gene Symbol** | **Zscore** |
| 427 | 58 | **NM_027677.2** | **Gpr39** | **-5,36** |
| 212 | 68 | **NM_010564.5** | **Inha** | **-4,72** |
| 129 | 15 | **NM_010889.1** | **Neb** | **-4,71** |
| 650 | 123 | **NM_021526.2** | **Psmd14** | **-4,55** |
| 150 | 45 | **NM_009321.2** | **Tbca** | **-4,44** |
| 2570 | 395 | **NM_031183.3** | **Sp6** | **-4,40** |
| 19953 | 2470 | **NM_025319.2** | **0610009B22Rik** | **-4,36** |
| 154 | 37 | **NM_010569.4** | **Invs** | **-4,34** |
| 442 | 150 | **NM_172424.4** | **Med13l** | **-4,32** |
| 3429 | 604 | **NM_172990.5** | **Pank4** | **-4,28** |
| 4746 | 896 | **NM_001159275.1** | **Slc25a2** | **-4,20** |
| 763 | 163 | **NM_010577.4** | **Itga5** | **-4,12** |
| 1807 | 431 | **NM_029172.1** | **Zc2hc1b** | **-4,06** |
| 2064 | 386 | **null** | **D530014G21Rik** | **-4,05** |
| 347 | 153 | **null** | **1700025H01Rik** | **-4,04** |
| 1493 | 342 | **NM_028788.4** | **Stra6l** | **-4,04** |
| 1476 | 299 | **NM_173350.3** | **Osbpl9** | **-4,04** |
| 2865 | 554 | **NM_007675.4** | **Ceacam10** | **-4,01** |
| 1821 | 402 | **NM_010240.2** | **Ftl1** | **-4,01** |
| 5325 | 1244 | **NM_011665.4** | **Ube2i** | **-4,00** |
| 268 | 103 | **NM_008116.3** | **Ggt1** | **-4,00** |
| 958 | 235 | **NM_134082.3** | **Farp1** | **-3,99** |
| 330 | 120 | **NM_023597.3** | **Wdr31** | **-3,93** |
| 97 | 33 | **null** | **1700101I11Rik** | **-3,93** |
| 114 | 47 | **NM_027341.2** | **Dzip3** | **-3,86** |
| 862 | 316 | **NM_022430.2** | **Ms4a8a** | **-3,84** |
| 502 | 170 | **NM_013547.3** | **Hgd** | **-3,82** |
| 2471 | 339 | **NM_031403.3** | **Dbr1** | **-3,81** |
| 311 | 131 | **NM_021893.3** | **Cd274** | **-3,81** |
| 1639 | 533 | **null** | **4933424L07Rik** | **-3,80** |
| 806 | 264 | **null** | **4930483K19Rik** | **-3,79** |
| 606 | 245 | **NM_175171.3** | **Mast4** | **-3,77** |
| 270 | 111 | **NM_023858.3** | **Mtmr2** | **-3,73** |
| 477 | 129 | **NM_008528.5** | **Blnk** | **-3,70** |
| 7286 | 1634 | **null** | **6330531I01Rik** | **-3,69** |
| 677 | 205 | **NM_153393.2** | **Col23a1** | **-3,66** |
| 1071 | 370 | **NM_001080942.2** | **Bsph2** | **-3,64** |
| 5454 | 745 | **NM_025756.1** | **4933427E11Rik** | **-3,59** |
| 1909 | 596 | **NM_013590.4** | **Lyz1** | **-3,59** |
| 209 | 101 | **NM_001007567.2** | **Slc7a6os** | **-3,58** |
| 232 | 88 | **NM_028509.1** | **1700034J05Rik** | **-3,57** |
| 655 | 265 | **NM_008854.5** | **Prkaca** | **-3,55** |
| 676 | 213 | **null** | **4930570E01Rik** | **-3,55** |
| 105 | 39 | **null** | **Igk-V21** | **-3,52** |
| 375 | 160 | **NM_028570.1** | **1700091H14Rik** | **-3,51** |
| 231 | 98 | **NM_013820.3** | **Hk2** | **-3,50** |
| 1349 | 361 | **null** | **2700038G22Rik** | **-3,50** |
| 7796 | 1290 | **NM_028900.4** | **Gcc1** | **-3,50** |
| 163 | 49 | **NM_020590.4** | **Gabarapl1** | **-3,48** |
| 2015 | 493 | **NM_027250.4** | **Coa7** | **-3,48** |
| 1320 | 516 | **NM_153123.2** | **Atf7ip2** | **-3,46** |
| 2511 | 678 | **NM_025564.2** | **Magohb** | **-3,45** |
| 89 | 35 | **null** | **2410087M07Rik** | **-3,44** |
| 365 | 189 | **NM_009191.4** | **Clpb** | **-3,44** |
| 1504 | 463 | **NM_008392.1** | **Acod1** | **-3,44** |
| 6726 | 1346 | **NM_029025.3** | **Tmem81** | **-3,42** |
| 1048 | 367 | **NM_018767.3** | **Cd160** | **-3,40** |
| 430 | 145 | **null** | **1700022C07Rik** | **-3,39** |
| 628 | 242 | **NM_030249.4** | **Cttnbp2nl** | **-3,37** |
| 288 | 116 | **NM_019779.4** | **Cyp11a1** | **-3,37** |
| 148 | 62 | **NM_028892.4** | **Spag17** | **-3,36** |
| 484 | 222 | **null** | **C030011I16Rik** | **-3,32** |
| 140 | 61 | **NM_025519.2** | **Chmp4c** | **-3,31** |
| 216 | 89 | **NM_026059.2** | **2900005J15Rik** | **-3,26** |
| 434 | 178 | **NM_025712.5** | **Snx31** | **-3,24** |
| 372 | 154 | **null** | **1700011C11Rik** | **-3,23** |
| 401 | 208 | **NM_025962.3** | **Mmachc** | **-3,23** |
| 654 | 237 | **NM_026907.3** | **Sectm1b** | **-3,22** |
| 419 | 181 | **null** | **Jpx** | **-3,20** |
| 4291 | 1362 | **NM_025376.3** | **Mymk** | **-3,18** |
| 461 | 214 | **NM_009680.3** | **Ap3b1** | **-3,18** |
| 1372 | 402 | **NM_008719.2** | **Npas2** | **-3,17** |
| 179 | 70 | **NM_024260.5** | **Vps50** | **-3,17** |
| 850 | 403 | **NM_031874.5** | **Rab3d** | **-3,16** |
| 958 | 330 | **NM_009628.3** | **Adnp** | **-3,16** |
| 105 | 42 | **NM_001080971.2** | **Tubb1** | **-3,14** |
| 240 | 130 | **NM_013596.2** | **Mc5r** | **-3,14** |
| 735 | 336 | **null** | **4930558N01Rik** | **-3,14** |
| 95 | 40 | **NM_183123.2** | **Spinkl** | **-3,14** |
| 560 | 232 | **null** | **Snhg7** | **-3,12** |
| 111 | 54 | **NM_023655.2** | **Trim29** | **-3,12** |
| 133 | 55 | **NM_010580.2** | **Itgb5** | **-3,11** |
| 1365 | 449 | **NM_030168.3** | **Rictor** | **-3,10** |
| 3114 | 1069 | **NM_030254.4** | **Tusc3** | **-3,10** |
| 103 | 48 | **NM_009196.4** | **Slc16a1** | **-3,09** |
| 3239 | 961 | **null** | **Redrum** | **-3,09** |
| 161 | 81 | **NM_007804.2** | **Cux2** | **-3,09** |
| 244 | 120 | **NM_010295.2** | **Gclc** | **-3,09** |
| 243 | 109 | **NM_026195.3** | **Atic** | **-3,07** |
| 571 | 321 | **NM_011309.3** | **S100a1** | **-3,07** |
| 268 | 115 | **null** | **1700049J03Rik** | **-3,06** |
| 118 | 61 | **null** | **1700030C12Rik** | **-3,06** |
| 13323 | 3315 | **NM_001007569.2** | **Pnmal1** | **-3,06** |
| 440 | 197 | **NM_028815.4** | **Cep97** | **-3,05** |
| 265 | 122 | **NM_007855.3** | **Twist2** | **-3,05** |
| 335 | 165 | **null** | **null** | **-3,05** |
| 95 | 44 | **NM_153792.2** | **Traf7** | **-3,05** |
| 264 | 128 | **NM_028326.1** | **Zfp618** | **-3,04** |
| 12766 | 2813 | **NM_028431.2** | **Pmpcb** | **-3,04** |
| 29 | 8 | **NM_029458.1** | **Hormad2** | **-3,04** |
| 726 | 259 | **NM_030685.3** | **Serp1** | **-3,02** |
| 294 | 179 | **null** | **9330154F10Rik** | **-3,02** |
| 829 | 244 | **NM_009549.3** | **Zfp185** | **-3,00** |
| 494 | 190 | **NM_001101471.1** | **Akap5** | **-3,00** |
| 1227 | 484 | **NM_010798.3** | **Mif** | **-3,00** |
| 6052 | 1328 | **null** | **9430065F17Rik** | **-2,99** |
| 79 | 38 | **NM_133665.4** | **Mef2d** | **-2,99** |
| 147 | 80 | **NM_021415.4** | **Cacna1h** | **-2,99** |
| 358 | 177 | **NM_144857.1** | **Rrp36** | **-2,98** |
| 457 | 200 | **null** | **1700018G05Rik** | **-2,98** |
| 81 | 32 | **NM_021275.4** | **Kcna4** | **-2,98** |
| 3650 | 1024 | **NM_029235.1** | **4930556L07Rik** | **-2,97** |
| 877 | 350 | **NM_027094.1** | **Dydc1** | **-2,97** |
| 134 | 61 | **null** | **2600010L24Rik** | **-2,97** |
| 82 | 33 | **NM_029371.1** | **Htatsf1** | **-2,96** |
| 454 | 204 | **NM_028039.2** | **Esco2** | **-2,96** |
| 3491 | 1323 | **NM_174992.4** | **Smagp** | **-2,95** |
| 304 | 155 | **NM_011204.2** | **Ptpn13** | **-2,93** |
| 1018 | 344 | **NM_028263.1** | **Fgfbp3** | **-2,92** |
| 1014 | 360 | **NM_001347138.1** | **Ccdc190** | **-2,92** |
| 61 | 21 | **NM_012009.5** | **Sh2d1b1** | **-2,92** |
| 233 | 115 | **NM_028943.5** | **Sgms2** | **-2,91** |
| 988 | 394 | **NM_023900.2** | **Plekhj1** | **-2,91** |
| 154 | 92 | **NM_145574.3** | **Ccdc136** | **-2,91** |
| 1112 | 512 | **NM_146035.2** | **Mgat2** | **-2,90** |
| 4361 | 1095 | **NM_009398.2** | **Tnfaip6** | **-2,90** |
| 28 | 8 | **NM_011564.1** | **Sry** | **-2,90** |
| 1183 | 483 | **null** | **4930519K11Rik** | **-2,89** |
| 390 | 166 | **NM_176999.3** | **Atp10b** | **-2,89** |
| 3673 | 1222 | **NM_133765.4** | **Fbxo31** | **-2,88** |
| 304 | 132 | **NM_026519.3** | **Emc4** | **-2,88** |
| 28 | 11 | **null** | **LOC102632821** | **-2,87** |
| 827 | 343 | **NM_008165.4** | **Gria1** | **-2,87** |
| 741 | 342 | **null** | **2900093K20Rik** | **-2,86** |
| 67 | 28 | **null** | **1700101I19Rik** | **-2,86** |
| 3401 | 954 | **NM_007761.2** | **Crcp** | **-2,86** |
| 1986 | 711 | **null** | **null** | **-2,86** |
| 286 | 146 | **NM_013815.2** | **Baz1a** | **-2,86** |
| 216 | 92 | **NM_175464.2** | **Pkp4** | **-2,85** |
| 132 | 69 | **NM_008245.3** | **Hhex** | **-2,84** |
| 1856 | 678 | **NM_133197.5** | **Mcf2** | **-2,84** |
| 3935 | 1527 | **NM_025655.2** | **Tmigd1** | **-2,84** |
| 507 | 203 | **NM_026124.3** | **Rab5if** | **-2,83** |
| 60 | 26 | **NM_025558.5** | **Cyb5b** | **-2,83** |
| 324 | 150 | **null** | **2900084C01Rik** | **-2,83** |
| 51 | 17 | **NM_023624.4** | **Lrat** | **-2,82** |
| 3796 | 1401 | **NM_001290273.1** | **Marc1** | **-2,82** |
| 115 | 57 | **NM_019481.2** | **Slc13a1** | **-2,82** |
| 107 | 47 | **NM_009554.4** | **Zfp37** | **-2,81** |
| 130 | 64 | **NM_021489.3** | **F12** | **-2,81** |
| 215 | 119 | **NM_026612.4** | **Ndufb2** | **-2,81** |
| 24 | 9 | **NM_016862.4** | **Vti1a** | **-2,79** |
| 1083 | 481 | **null** | **LOC100901407** | **-2,79** |
| 518 | 213 | **null** | **1700037F03Rik** | **-2,78** |
| 396 | 192 | **NM_133840.3** | **Clp1** | **-2,78** |
| 3538 | 1001 | **NM_007878.3** | **Drd4** | **-2,77** |
| 120 | 63 | **null** | **null** | **-2,77** |
| 474 | 198 | **NM_022025.4** | **Slc5a7** | **-2,77** |
| 2027 | 591 | **NM_029809.2** | **Rnf225** | **-2,77** |
| 1907 | 676 | **NM_178071.6** | **Nme7** | **-2,77** |
| 311 | 173 | **null** | **4930529N20Rik** | **-2,76** |
| 1269 | 483 | **NM_010394.4** | **H2-Q7** | **-2,76** |
| 168 | 79 | **NM_197986.2** | **Tmem140** | **-2,76** |
| 5792 | 1965 | **NM_008023.1** | **Foxb2** | **-2,76** |
| 2051 | 762 | **NM_009361.3** | **Tfdp1** | **-2,75** |
| 55 | 24 | **null** | **2610016A17Rik** | **-2,75** |
| 2613 | 881 | **null** | **4930448K20Rik** | **-2,74** |
| 1309 | 622 | **NM_011381.4** | **Six3** | **-2,74** |
| 222 | 103 | **NM_028835.4** | **Atg7** | **-2,74** |
| 542 | 289 | **NM_172652.3** | **Kansl3** | **-2,74** |
| 78 | 37 | **NM_011956.3** | **Nubp2** | **-2,73** |
| 708 | 408 | **null** | **4930554C24Rik** | **-2,73** |
| 90 | 44 | **null** | **4930428D20Rik** | **-2,73** |
| 75 | 38 | **NM_008760.4** | **Ogn** | **-2,72** |
| 1631 | 709 | **NM_029376.2** | **Speer4a** | **-2,71** |
| 143 | 70 | **NM_011898.3** | **Spry4** | **-2,71** |
| 1075 | 429 | **NM_025882.3** | **Pole4** | **-2,71** |
| 5654 | 1664 | **NM_026309.2** | **Lsm3** | **-2,71** |
| 122 | 71 | **null** | **3110027N22Rik** | **-2,71** |
| 561 | 319 | **null** | **null** | **-2,70** |
| 520 | 210 | **NM_009731.2** | **Akr1b7** | **-2,70** |
| 165 | 88 | **null** | **4930433B08Rik** | **-2,70** |
| 1335 | 566 | **NM_011944.3** | **Map2k7** | **-2,69** |
| 275 | 148 | **NM_029021.1** | **Ocstamp** | **-2,68** |
| 98 | 50 | **NM_025505.4** | **Blzf1** | **-2,68** |
| 1715 | 859 | **NM_025273.4** | **Pcbd1** | **-2,68** |
| 78 | 46 | **null** | **3110005L24Rik** | **-2,67** |
| 347 | 168 | **NM_009931.2** | **Col4a1** | **-2,66** |
| 1134 | 475 | **NM_030021.3** | **Cutal** | **-2,66** |
| 1017 | 365 | **NM_027926.3** | **Cpa4** | **-2,66** |
| 12428 | 4496 | **NM_023556.3** | **Mvk** | **-2,65** |
| 1299 | 483 | **null** | **Igh-V7183** | **-2,65** |
| 7025 | 2303 | **NM_010600.3** | **Kcnh1** | **-2,64** |
| 90 | 53 | **NM_007604.3** | **Capza2** | **-2,64** |
| 518 | 283 | **null** | **Igh-VJ558** | **-2,64** |
| 621 | 257 | **NM_026083.2** | **Zc3h13** | **-2,63** |
| 187 | 86 | **null** | **4933425B07Rik** | **-2,63** |
| 413 | 210 | **NM_011828.4** | **Hs2st1** | **-2,63** |
| 70 | 35 | **null** | **4930425O10Rik** | **-2,63** |
| 448 | 206 | **NM_175225.5** | **Tasp1** | **-2,62** |
| 46 | 11 | **NM_177821.6** | **Ep300** | **-2,62** |
| 858 | 472 | **null** | **1700127F24Rik** | **-2,61** |
| 169 | 94 | **NM_001163061.1** | **Zfp949** | **-2,60** |
| 174 | 104 | **NM_001319156.1** | **Ryr3** | **-2,60** |
| 182 | 114 | **NM_008097.2** | **Gcdh** | **-2,60** |
| 747 | 401 | **NM_009142.3** | **Cx3cl1** | **-2,60** |
| 80 | 45 | **null** | **4930573O16Rik** | **-2,60** |
| 75 | 42 | **NM_009364.4** | **Tfpi2** | **-2,60** |
| 87 | 42 | **NM_030150.2** | **Dhx58** | **-2,60** |
| 512 | 202 | **NM_007688.2** | **Cfl2** | **-2,59** |
| 853 | 329 | **NM_009677.6** | **Ap1g1** | **-2,59** |
| 216 | 106 | **NM_016757.2** | **Wbp1** | **-2,58** |
| 3497 | 1286 | **NM_029631.3** | **Abhd14b** | **-2,58** |
| 140 | 80 | **NM_028004.2** | **Ttn** | **-2,58** |
| 107 | 51 | **NM_011855.4** | **Tenm1** | **-2,58** |
| 2836 | 1048 | **NM_018821.4** | **Socs6** | **-2,58** |
| 4148 | 1191 | **NM_016959.4** | **Rps3a1** | **-2,57** |
| 578 | 295 | **NM_054063.4** | **Psg28** | **-2,56** |
| 128 | 75 | **null** | **Igkv4-77** | **-2,56** |
| 186 | 87 | **null** | **4921501M06Rik** | **-2,56** |
| 169 | 104 | **NM_011803.2** | **Klf6** | **-2,55** |
| 292 | 152 | **NM_010188.5** | **Fcgr3** | **-2,55** |
| 174 | 90 | **null** | **9330198N18Rik** | **-2,55** |
| 233 | 120 | **NM_022311.2** | **Tcte2** | **-2,54** |
| 203 | 106 | **NM_175215.4** | **Lysmd4** | **-2,54** |
| 99 | 51 | **NM_011802.3** | **Clpx** | **-2,54** |
| 132 | 74 | **NM_021788.2** | **Sap30** | **-2,53** |
| 744 | 303 | **NM_029037.4** | **Pomk** | **-2,53** |
| 112 | 59 | **null** | **4930440C22Rik** | **-2,53** |
| 108 | 62 | **NM_134109.2** | **Ildr1** | **-2,53** |
| 773 | 384 | **NM_025770.3** | **Atg10** | **-2,53** |
| 160 | 87 | **null** | **4633402D09Rik** | **-2,53** |
| 416 | 215 | **NM_021481.3** | **Treh** | **-2,52** |
| 137 | 74 | **NM_011915.2** | **Wif1** | **-2,52** |
| 605 | 274 | **NM_133966.3** | **Taf5l** | **-2,52** |
| 586 | 264 | **null** | **1500002J14Rik** | **-2,52** |
| 44 | 21 | **NM_010440.3** | **Hmg20b** | **-2,52** |
| 77 | 26 | **NM_054079.2** | **Iltifb** | **-2,51** |
| 157 | 86 | **NM_172403.2** | **2810021J22Rik** | **-2,51** |
| 646 | 302 | **NM_011701.4** | **Vim** | **-2,51** |
| 128 | 66 | **NM_023113.5** | **Aspa** | **-2,51** |
| 81 | 38 | **null** | **2700008E08Rik** | **-2,50** |
| 85 | 41 | **NM_009567.4** | **Zfp93** | **-2,50** |
| 337 | 147 | **null** | **Igh-V7183** | **-2,50** |
| 2122 | 1052 | **NM_175244.4** | **Hectd3** | **-2,50** |
| 171 | 104 | **NM_030713.2** | **Zfp202** | **-2,49** |
| 6527 | 2331 | **null** | **1700085D22Rik** | **-2,49** |
| 2589 | 1007 | **NM_199241.3** | **Sema6d** | **-2,49** |
| 75 | 40 | **null** | **4930579G18Rik** | **-2,49** |
| 153 | 86 | **NM_029922.3** | **Parp6** | **-2,49** |
| 415 | 207 | **NM_013730.4** | **Slamf1** | **-2,49** |
| 493 | 198 | **NM_146027.2** | **Scrn2** | **-2,48** |
| 570 | 305 | **NM_009674.3** | **Anxa7** | **-2,48** |
| 194 | 113 | **null** | **1700085B13Rik** | **-2,48** |
| 450 | 257 | **NM_025546.2** | **Rsl1d1** | **-2,48** |
| 1060 | 397 | **NM_013546.3** | **Hebp1** | **-2,47** |
| 944 | 395 | **null** | **4930594C11Rik** | **-2,47** |
| 194 | 113 | **NM_010792.1** | **Mettl1** | **-2,47** |
| 388 | 227 | **null** | **4930563M20Rik** | **-2,47** |
| 9795 | 4115 | **NM_027041.4** | **Cfap206** | **-2,47** |
| 382 | 229 | **NM_021528.3** | **Chst12** | **-2,47** |
| 133 | 81 | **NM_001360520.1** | **Slc4a7** | **-2,47** |
| 96 | 46 | **NM_027513.1** | **Nup205** | **-2,46** |
| 6707 | 2555 | **NM_008450.2** | **Klc1** | **-2,46** |
| 488 | 244 | **NM_010583.3** | **Itk** | **-2,46** |
| 437 | 243 | **NM_172263.2** | **Pde8b** | **-2,46** |
| 128 | 69 | **null** | **1110065H08Rik** | **-2,46** |
| 2138 | 925 | **NM_133975.4** | **Trip12** | **-2,45** |
| 1166 | 479 | **NM_008060.2** | **Ganab** | **-2,45** |
| 335 | 207 | **null** | **null** | **-2,45** |
| 783 | 417 | **NM_010230.2** | **Fmn1** | **-2,45** |
| 4548 | 1869 | **NM_023386.5** | **Rtp4** | **-2,45** |
| 128 | 55 | **null** | **5730437C12Rik** | **-2,45** |
| 1417 | 629 | **NM_013742.5** | **Cars** | **-2,44** |
| 388 | 198 | **NM_028176.1** | **Cda** | **-2,44** |
| 23176 | 8900 | **NM_026527.3** | **Chac2** | **-2,44** |
| 178 | 93 | **NM_021487.1** | **Kcne1l** | **-2,43** |
| 254 | 137 | **NM_025407.2** | **Uqcrc1** | **-2,43** |
| 1149 | 477 | **NM_025763.1** | **4933436I01Rik** | **-2,43** |
| 1609 | 644 | **null** | **4930467J12Rik** | **-2,43** |
| 526 | 261 | **null** | **C030026O17Rik** | **-2,43** |
| 1106 | 480 | **NM_183162.2** | **Helz2** | **-2,42** |
| 87 | 54 | **null** | **0610039K10Rik** | **-2,42** |
| 155 | 82 | **NM_011182.4** | **Cyth3** | **-2,40** |
| 109 | 72 | **NM_009163.4** | **Sgpl1** | **-2,40** |
| 6901 | 1925 | **NM_010069.1** | **Doc2a** | **-2,40** |
| 25 | 9 | **NM_013457.4** | **Add1** | **-2,40** |
| 382 | 229 | **NM_172402.3** | **Slc25a32** | **-2,40** |
| 5268 | 1463 | **NM_007786.5** | **Csn3** | **-2,40** |
| 482 | 252 | **NM_011602.5** | **Tln1** | **-2,39** |
| 1396 | 665 | **NM_028982.4** | **Fam234b** | **-2,39** |
| 282 | 165 | **NM_026228.5** | **Slc39a8** | **-2,39** |
| 594 | 236 | **NM_029384.1** | **Tmem238** | **-2,38** |
| 200 | 102 | **NM_133767.3** | **Mtif2** | **-2,38** |
| 111 | 51 | **NM_023876.4** | **Elp4** | **-2,38** |
| 125 | 68 | **NM_001162923.1** | **Rasl12** | **-2,38** |
| 1512 | 711 | **null** | **9030625N01Rik** | **-2,38** |
| 90 | 47 | **null** | **A430106A12Rik** | **-2,38** |
| 8982 | 3645 | **NM_009424.3** | **Traf6** | **-2,38** |
| 261 | 142 | **NM_008920.4** | **Prg2** | **-2,37** |
| 46 | 18 | **NM_025620.2** | **Rep15** | **-2,37** |
| 2716 | 1112 | **null** | **1700010K24Rik** | **-2,37** |
| 2391 | 964 | **NM_028230.4** | **Shmt2** | **-2,37** |
| 23 | 9 | **NM_007524.3** | **Nkx3-2** | **-2,37** |
| 2332 | 780 | **null** | **4930528H21Rik** | **-2,37** |
| 2022 | 858 | **NM_012051.4** | **Etv3** | **-2,36** |
| 8506 | 3521 | **NM_029704.2** | **Ttc19** | **-2,36** |
| 114 | 61 | **NM_028990.5** | **Tmem168** | **-2,36** |
| 180 | 96 | **NM_029205.2** | **Spaca6** | **-2,36** |
| 41 | 13 | **NM_008674.2** | **Nat3** | **-2,36** |
| 1238 | 578 | **NM_145154.2** | **Angptl6** | **-2,36** |
| 92 | 56 | **NM_177284.2** | **Nrxn1** | **-2,36** |
| 68 | 36 | **NM_018855.2** | **Gas8** | **-2,35** |
| 252 | 174 | **NM_152839.3** | **Jchain** | **-2,35** |
| 104 | 61 | **NM_001007570.2** | **Slc25a42** | **-2,35** |
| 493 | 197 | **NM_053262.3** | **Hsd17b11** | **-2,35** |
| 2616 | 1039 | **NM_144858.2** | **Dus3l** | **-2,34** |
| 1380 | 682 | **null** | **1700080G11Rik** | **-2,34** |
| 220 | 120 | **null** | **1700063J08Rik** | **-2,34** |
| 1895 | 797 | **null** | **4930431L21Rik** | **-2,34** |
| 6932 | 3109 | **NM_181649.6** | **Gpatch11** | **-2,34** |
| 82 | 41 | **NM_008309.5** | **Htr1d** | **-2,34** |
| 54 | 32 | **NM_027492.2** | **Naa30** | **-2,34** |
| 425 | 249 | **null** | **4930432O09Rik** | **-2,34** |
| 161 | 86 | **NM_011691.4** | **Vav1** | **-2,33** |
| 25539 | 11465 | **NM_013797.5** | **Slco1a1** | **-2,33** |
| 195 | 121 | **NM_007811.2** | **Cyp26a1** | **-2,33** |
| 552 | 223 | **null** | **E130201H02Rik** | **-2,33** |
| 423 | 239 | **null** | **C030013D06Rik** | **-2,33** |
| 658 | 363 | **NM_009088.3** | **Polr1a** | **-2,33** |
| 2606 | 1073 | **NM_001362062.1** | **Zfp746** | **-2,32** |
| 159 | 95 | **NM_153406.3** | **Specc1l** | **-2,32** |
| 122 | 78 | **NM_028821.3** | **Dnal1** | **-2,32** |
| 2998 | 1074 | **NM_010685.4** | **Lamp2** | **-2,31** |
| 122 | 78 | **null** | **2900092N11Rik** | **-2,31** |
| 1336 | 477 | **NM_025372.4** | **Tipin** | **-2,31** |
| 786 | 397 | **NM_032398.2** | **Plvap** | **-2,31** |
| 27 | 10 | **null** | **Defa16** | **-2,30** |
| 994 | 468 | **NM_029633.2** | **Clasp2** | **-2,30** |
| 548 | 248 | **NM_144536.3** | **Cdkal1** | **-2,30** |
| 169 | 105 | **NM_053181.3** | **Pdxdc1** | **-2,30** |
| 1962 | 991 | **NM_010719.5** | **Lipe** | **-2,30** |
| 181 | 102 | **NM_194055.3** | **Esrp1** | **-2,29** |
| 153 | 88 | **NM_027158.1** | **Upk3bl** | **-2,29** |
| 782 | 400 | **NM_026845.4** | **Ppil1** | **-2,29** |
| 77 | 45 | **NM_007384.3** | **Asic2** | **-2,29** |
| 1920 | 713 | **NM_134156.2** | **Actn1** | **-2,29** |
| 3363 | 1314 | **NM_133986.2** | **Tcta** | **-2,29** |
| 27 | 10 | **NM_007826.3** | **Dach1** | **-2,28** |
| 1171 | 516 | **null** | **C030004M13Rik** | **-2,28** |
| 69 | 34 | **NM_009131.3** | **Clec11a** | **-2,28** |
| 749 | 372 | **null** | **4930552F14Rik** | **-2,27** |
| 4991 | 1532 | **NM_008601.3** | **Mitf** | **-2,27** |
| 1341 | 707 | **null** | **6030442E23Rik** | **-2,27** |
| 296 | 180 | **NM_016843.4** | **Atxn10** | **-2,27** |
| 8485 | 3348 | **NM_011692.2** | **Vbp1** | **-2,27** |
| 146 | 88 | **null** | **1700019P21Rik** | **-2,27** |
| 1038 | 429 | **null** | **1700052M18Rik** | **-2,27** |
| 99 | 53 | **NM_172252.4** | **Mrpl21** | **-2,27** |
| 251 | 141 | **NM_019536.1** | **Dnah10** | **-2,27** |
| 959 | 448 | **NM_030243.5** | **Rbm43** | **-2,26** |
| 237 | 135 | **NM_001042670.1** | **Mterf1b** | **-2,26** |
| 980 | 474 | **NM_178577.5** | **Tmem205** | **-2,26** |
| 1596 | 689 | **null** | **4930520O04Rik** | **-2,26** |
| 460 | 209 | **NM_023816.2** | **Ankrd36** | **-2,25** |
| 69 | 37 | **NM_025921.3** | **2610002M06Rik** | **-2,25** |
| 255 | 133 | **NM_008365.2** | **Il18r1** | **-2,25** |
| 161 | 99 | **null** | **AF357399** | **-2,25** |
| 86 | 45 | **NM_001270496.1** | **Tmem254b** | **-2,25** |
| 191 | 112 | **NM_133768.5** | **Asl** | **-2,25** |
| 363 | 199 | **NM_021280.3** | **Plcg1** | **-2,25** |
| 562 | 293 | **NM_026486.3** | **Tctn2** | **-2,24** |
| 65 | 37 | **NM_133435.2** | **Nmnat1** | **-2,24** |
| 332 | 206 | **NM_134015.3** | **Fbxw11** | **-2,24** |
| 517 | 260 | **NM_026084.2** | **3110070M22Rik** | **-2,24** |
| 557 | 253 | **NM_025387.3** | **Tmem14c** | **-2,24** |
| 21 | 8 | **NM_010729.3** | **Loxl1** | **-2,24** |
| 1085 | 623 | **NM_024282.3** | **Desi2** | **-2,24** |
| 159 | 84 | **NM_001081097.2** | **Grik3** | **-2,24** |
| 655 | 335 | **NM_146118.3** | **Slc25a25** | **-2,24** |
| 1055 | 426 | **NM_026367.4** | **Gpatch2** | **-2,24** |
| 1441 | 639 | **NM_144958.4** | **Eif4a1** | **-2,24** |
| 277 | 145 | **NM_178726.3** | **Ppm1l** | **-2,23** |
| 198 | 101 | **NM_010311.4** | **Gnaz** | **-2,23** |
| 104 | 62 | **null** | **1700003H04Rik** | **-2,23** |
| 801 | 416 | **null** | **null** | **-2,23** |
| 932 | 447 | **NM_016666.3** | **Aip** | **-2,23** |
| 543 | 250 | **NM_025868.4** | **Tmx2** | **-2,23** |
| 110 | 71 | **null** | **6720483E21Rik** | **-2,22** |
| 662 | 326 | **null** | **1700039I01Rik** | **-2,22** |
| 250 | 123 | **NM_008581.1** | **Mela** | **-2,22** |
| 208 | 143 | **null** | **Gm12238** | **-2,21** |
| 5679 | 2212 | **null** | **8430439B09Rik** | **-2,21** |
| 3736 | 1334 | **NM_009737.3** | **Bcat2** | **-2,21** |
| 429 | 239 | **NM_025438.2** | **Rsrc2** | **-2,21** |
| 384 | 221 | **NM_026496.4** | **Grhl2** | **-2,20** |
| 21 | 8 | **NM_026925.4** | **Pnlip** | **-2,20** |
| 189 | 125 | **NM_007569.2** | **Btg1** | **-2,20** |
| 87 | 53 | **NM_144542.1** | **Esco1** | **-2,20** |
| 429 | 249 | **NM_010769.2** | **Matn1** | **-2,20** |
| 1823 | 737 | **NM_030565.6** | **Fam20c** | **-2,20** |
| 98 | 62 | **NM_028801.2** | **Muc5b** | **-2,20** |
| 2489 | 1051 | **NM_011232.3** | **Rad1** | **-2,20** |
| 53 | 25 | **NM_134078.4** | **Chmp7** | **-2,20** |
| 5076 | 2258 | **NM_053098.2** | **Lmod2** | **-2,20** |
| 3903 | 1628 | **NM_028923.3** | **Gle1** | **-2,19** |
| 258 | 151 | **NM_175240.4** | **Fam187b** | **-2,19** |
| 5045 | 1898 | **null** | **A730035I17Rik** | **-2,19** |
| 71 | 43 | **NM_028752.3** | **Hvcn1** | **-2,19** |
| 1607 | 528 | **null** | **9330169L03Rik** | **-2,19** |
| 770 | 437 | **NM_019454.3** | **Dll4** | **-2,19** |
| 131 | 76 | **NM_178111.3** | **Trp53inp2** | **-2,19** |
| 64 | 33 | **NM_001163615.1** | **Krtap20-2** | **-2,19** |
| 144 | 99 | **NM_175152.4** | **Thap3** | **-2,18** |
| 130 | 79 | **NM_020026.4** | **B3galnt1** | **-2,17** |
| 778 | 417 | **NM_009171.2** | **Shmt1** | **-2,17** |
| 550 | 310 | **NM_145149.4** | **Rasgrp4** | **-2,17** |
| 437 | 184 | **NM_028611.3** | **Ndufaf7** | **-2,17** |
| 140 | 86 | **null** | **A230083N12Rik** | **-2,17** |
| 98 | 60 | **NM_001160386.1** | **Dnah7b** | **-2,17** |
| 2508 | 986 | **NM_023894.1** | **Rhox9** | **-2,16** |
| 169 | 103 | **NM_026108.3** | **Pudp** | **-2,16** |
| 6886 | 2635 | **null** | **2700033N17Rik** | **-2,16** |
| 400 | 225 | **NM_016736.3** | **Nub1** | **-2,16** |
| 80 | 42 | **null** | **0610008J02Rik** | **-2,16** |
| 704 | 287 | **null** | **Rab26os** | **-2,15** |
| 336 | 210 | **NM_139229.4** | **Cog8** | **-2,15** |
| 178 | 104 | **null** | **1700010J16Rik** | **-2,15** |
| 10858 | 4047 | **null** | **null** | **-2,15** |
| 235 | 123 | **NM_019961.3** | **Pex3** | **-2,15** |
| 280 | 152 | **NM_019460.2** | **Sfmbt1** | **-2,15** |
| 195 | 124 | **NM_007459.3** | **Ap2a2** | **-2,15** |
| 214 | 123 | **NM_007512.4** | **Atpif1** | **-2,15** |
| 1054 | 503 | **null** | **5730405A17Rik** | **-2,14** |
| 11448 | 4625 | **NM_026549.4** | **Pdcd2l** | **-2,14** |
| 2312 | 1028 | **null** | **C030001C17Rik** | **-2,14** |
| 240 | 127 | **NM_133724.3** | **Bloc1s4** | **-2,14** |
| 1623 | 887 | **null** | **6720490N10Rik** | **-2,14** |
| 718 | 367 | **null** | **4930481A15Rik** | **-2,14** |
| 632 | 333 | **NM_183089.3** | **Dscc1** | **-2,14** |
| 72 | 40 | **NM_001002894.2** | **Nlrp14** | **-2,14** |
| 9660 | 4284 | **NM_025584.2** | **Cd99** | **-2,14** |
| 714 | 411 | **NM_027922.2** | **Ankle2** | **-2,13** |
| 180 | 90 | **NM_019417.3** | **Pdlim4** | **-2,13** |
| 169 | 99 | **null** | **1810059H22Rik** | **-2,13** |
| 195 | 125 | **NM_026360.3** | **Ddx47** | **-2,13** |
| 1059 | 477 | **NM_027815.4** | **Vps35l** | **-2,12** |
| 1409 | 651 | **NM_008343.2** | **Igfbp3** | **-2,12** |
| 54 | 33 | **NM_145151.3** | **Crebzf** | **-2,12** |
| 222 | 140 | **NM_028632.2** | **Fcf1** | **-2,11** |
| 485 | 264 | **NM_198645.2** | **Ccdc58** | **-2,11** |
| 91 | 48 | **NM_026278.3** | **Lrp2bp** | **-2,11** |
| 394 | 243 | **null** | **4933405E24Rik** | **-2,11** |
| 205 | 145 | **null** | **2900041H08Rik** | **-2,11** |
| 297 | 185 | **NM_013545.3** | **Ptpn6** | **-2,11** |
| 428 | 251 | **NM_015781.5** | **Nap1l1** | **-2,11** |
| 1360 | 652 | **NM_008285.4** | **Hrh1** | **-2,10** |
| 1434 | 630 | **NM_138653.1** | **Bspry** | **-2,10** |
| 274 | 156 | **NM_177739.3** | **Zfp507** | **-2,10** |
| 74 | 42 | **NM_028927.3** | **Tktl2** | **-2,10** |
| 21 | 9 | **null** | **1600012P17Rik** | **-2,10** |
| 191 | 98 | **NM_033075.3** | **D17H6S56E-5** | **-2,10** |
| 3898 | 1796 | **NM_008929.3** | **Dnajc3** | **-2,10** |
| 117 | 63 | **NM_025588.2** | **Exoc2** | **-2,10** |
| 105 | 73 | **NM_027421.3** | **Ints2** | **-2,09** |
| 496 | 301 | **NM_007944.3** | **Eps15l1** | **-2,09** |
| 3832 | 1907 | **NM_009228.2** | **Snta1** | **-2,09** |
| 128 | 84 | **NM_008768.2** | **Orm1** | **-2,09** |
| 231 | 123 | **NM_025951.3** | **Pi4k2b** | **-2,09** |
| 3536 | 1641 | **null** | **null** | **-2,09** |
| 250 | 148 | **NM_145595.2** | **Cbr4** | **-2,09** |
| 217 | 128 | **null** | **1700121L03Rik** | **-2,08** |
| 1907 | 1013 | **NM_029932.3** | **Spns3** | **-2,08** |
| 1752 | 686 | **NM_028796.1** | **Rab10os** | **-2,08** |
| 1064 | 635 | **NM_009942.2** | **Cox5b** | **-2,08** |
| 4878 | 1636 | **NM_011503.5** | **Stxbp2** | **-2,08** |
| 2521 | 1093 | **NM_016898.2** | **Cd164** | **-2,08** |
| 352 | 176 | **NM_053071.2** | **Cox6c** | **-2,08** |
| 84 | 51 | **NM_009208.3** | **Slc4a3** | **-2,07** |
| 33 | 11 | **null** | **LOC641025** | **-2,07** |
| 244 | 155 | **NM_028914.1** | **Terb2** | **-2,07** |
| 6935 | 2794 | **NM_007392.3** | **Acta2** | **-2,07** |
| 606 | 322 | **null** | **4930485G23Rik** | **-2,07** |
| 26 | 10 | **NM_007616.4** | **Cav1** | **-2,06** |
| 82 | 53 | **NM_025786.3** | **Rnf186** | **-2,06** |
| 47 | 23 | **NM_054090.1** | **Olfr73** | **-2,06** |
| 1684 | 742 | **NM_010392.2** | **H2-Q2** | **-2,06** |
| 1386 | 659 | **NM_153503.2** | **Rnf113a1** | **-2,06** |
| 83 | 50 | **NM_026369.2** | **Arpc5** | **-2,06** |
| 130 | 78 | **NM_029557.1** | **Tsen54** | **-2,06** |
| 619 | 390 | **NM_178613.3** | **Gskip** | **-2,06** |
| 105 | 73 | **NM_008986.2** | **Cavin1** | **-2,06** |
| 107 | 59 | **NM_028314.2** | **2700097O09Rik** | **-2,06** |
| 345 | 147 | **NM_011119.3** | **Pa2g4** | **-2,05** |
| 2978 | 1072 | **NM_145400.3** | **Ube4a** | **-2,05** |
| 82 | 53 | **null** | **1700121I08Rik** | **-2,05** |
| 63 | 37 | **NM_011889.3** | **Sept3** | **-2,05** |
| 2367 | 1022 | **NM_025974.2** | **Rpl14** | **-2,05** |
| 1305 | 722 | **NM_028859.1** | **Ccdc182** | **-2,05** |
| 33 | 11 | **NM_011360.3** | **Sgce** | **-2,05** |
| 52 | 29 | **NM_013773.2** | **Tcl1b1** | **-2,05** |
| 1118 | 499 | **NM_010319.3** | **Gng7** | **-2,04** |
| 83 | 56 | **NM_013561.2** | **Htr3a** | **-2,04** |
| 2303 | 1050 | **NM_026146.4** | **Eps8l1** | **-2,04** |
| 9118 | 2934 | **NM_021713.2** | **Myg1** | **-2,04** |
| 353 | 235 | **NM_011487.5** | **Stat4** | **-2,04** |
| 45 | 23 | **NM_030740.1** | **Vmn1r56** | **-2,04** |
| 94 | 57 | **NM_025946.6** | **Romo1** | **-2,03** |
| 841 | 453 | **NM_030180.2** | **Usp54** | **-2,03** |
| 58 | 32 | **NM_027371.3** | **Rpf1** | **-2,03** |
| 2694 | 1279 | **NM_008609.4** | **Mmp15** | **-2,03** |
| 70 | 45 | **NM_001008798.1** | **Whrn** | **-2,02** |
| 15680 | 5320 | **NM_027353.4** | **Cd2bp2** | **-2,02** |
| 61 | 31 | **NM_053152.2** | **Klra22** | **-2,02** |
| 69 | 38 | **NM_008574.4** | **Smcp** | **-2,02** |
| 45 | 23 | **NM_008607.2** | **Mmp13** | **-2,02** |
| 15028 | 6701 | **NM_008830.2** | **Abcb4** | **-2,02** |
| 60 | 35 | **NM_025738.3** | **Cypt1** | **-2,02** |
| 88 | 55 | **null** | **4933437I04Rik** | **-2,02** |
| 3618 | 1813 | **NM_008342.3** | **Igfbp2** | **-2,02** |
| 1032 | 514 | **NM_008232.3** | **Hdgfl1** | **-2,02** |
| 6520 | 3511 | **NM_025310.3** | **Ftsj3** | **-2,02** |
| 78 | 45 | **NM_026348.4** | **Itgb3bp** | **-2,01** |
| 78 | 43 | **null** | **2610021J01Rik** | **-2,01** |
| 26252 | 13142 | **NM_025404.3** | **Arl4d** | **-2,01** |
| 1614 | 621 | **NM_019979.2** | **Selenok** | **-2,01** |
| 7608 | 3094 | **NM_028568.1** | **Dusp21** | **-2,01** |
| 64 | 35 | **NM_013529.3** | **Gfpt2** | **-2,01** |
| 523 | 291 | **NM_018888.4** | **Uqcc1** | **-2,01** |
| 1537 | 733 | **NM_028198.2** | **Xpo5** | **-2,00** |
| 240 | 144 | **NM_001008798.1** | **Whrn** | **-2,00** |
| 782 | 408 | **NM_009204.2** | **Slc2a4** | **-2,00** |
| 70 | 43 | **NM_028121.2** | **Adpgk** | **-2,00** |
| 21926 | 10363 | **NM_009226.4** | **Snrpd1** | **-2,00** |
| 2429 | 1102 | **NM_026921.4** | **Isca1** | **-2,00** |
| 83 | 50 | **null** | **4930597A21Rik** | **-2,00** |
| 538 | 323 | **NM_009298.4** | **Surf6** | **-2,00** |
| 3679 | 1731 | **NM_026592.4** | **B230118H07Rik** | **-2,00** |
| 224 | 126 | **null** | **2310034P14Rik** | **-2,00** |
