# Supplementary material for: Temporal Integrative Analysis of mRNA and microRNAs Expression Profiles and Epigenetic Alterations in Female SAMP8, a Model of Age-Related Cognitive Decline
Source: Front Genet. 2018 Dec 11;9:596. doi: 10.3389/fgene.2018.00596 (PMC6297390; doi:10.3389/fgene.2018.00596)
Supplement: Supplementary file 4 [file Table_4.docx]

Supplementary material 4. MicroRNAs differentially expressed in the hippocampus of SAMP8 compared to SAMR1 at 2 and 9 months of age.

| miRNAs | t-test R1vsP8  (9 months) | t-test R1vsP8  (2 months) | SAMP8 Mean  (9 months) | SAMP8 Mean  (2 months) |
| --- | --- | --- | --- | --- |
| *mmu-let-7b-5p* | **0,046213337** | **0,025296738** | 0,710930997 | 1,31154142 |
| *mmu-let-7c-5p* | **0,019629361** | 0,72939246 | 0,580510171 | 1,079552392 |
| *mmu-let-7d-5p* | **0,022263707** | 0,991917977 | 0,707097411 | 1,000488694 |
| *mmu-let-7e-5p* | **0,054712499** | **0,05** | 0,738337767 | 1,504667839 |
| *mmu-let-7i-5p* | 0,985963312 | 0,664759186 | 1,051096173 | 0,947546061 |
| *mmu-miR-101a-3p* | 0,645120903 | 0,728315948 | 3,784539443 | 0,986977272 |
| *mmu-miR-101b-3p* | **0,034074277** | **0,020054446** | 3,520195675 | 0,59846885 |
| *mmu-miR-105* | 0,16637105 | 0,082844693 | 0,519606206 | 0,313553321 |
| *mmu-miR-106b-5p* | 0,106624014 | 0,123178035 | 1,861689423 | 0,720407427 |
| *mmu-miR-107-3p* | **0,049595602** | **0,038757862** | 1,397608006 | 0,615645199 |
| *mmu-miR-124-3p* | 0,97359897 | 0,714906916 | 1,620876471 | 0,918021702 |
| *mmu-miR-125b-5p* | 0,799718142 | 0,373215369 | 1,005857508 | 0,002643986 |
| *mmu-miR-126a-5p* | 0,548492059 | 0,120284403 | 1,213842047 | 0,673524603 |
| *mmu-miR-128-3p* | **0,049137195** | 0,107497101 | 1,398217456 | 0,70290111 |
| *mmu-miR-130a-3p* | **0,026766511** | 0,337093524 | 2,361510061 | 1,210941056 |
| *mmu-miR-132-3p* | 0,809432696 | **0,049157255** | 0,959083619 | 0,836497347 |
| *mmu-miR-133b-3p* | 0,816927307 | 0,105806215 | 2,209415377 | 0,482747436 |
| *mmu-miR-134-5p* | 0,99449877 | **0,00031572** | 1,178201679 | 0,621953559 |
| *mmu-miR-135b-5p* | 0,131056431 | 0,841520837 | 1,149201989 | 1,055520091 |
| *mmu-miR-138-5p* | 0,624565332 | 0,319366317 | 2,040685568 | 0,848799131 |
| *mmu-miR-139-5p* | 0,790963378 | 0,451187424 | 0,974049411 | 0,853347148 |
| *mmu-miR-140-5p* | **0,047064629** | 0,082621657 | 2,616729417 | 0,606738693 |
| *mmu-miR-146a-5p* | 0,500429997 | **0,005989177** | 0,827438499 | 0,436161751 |
| *mmu-miR-146b-5p* | 0,77626551 | 0,454660558 | 0,961655797 | 0,897350044 |
| *mmu-miR-148b-3p* | **0,045045696** | **0,019782919** | 1,498560482 | 1,38452976 |
| *mmu-miR-151-3p* | **0,042968227** | **0,031549256** | 1,627757318 | 1,209669776 |
| *mmu-miR-152-3p* | 0,173723656 | 0,513865669 | 0,757629554 | 1,118562014 |
| *mmu-miR-15a-5p* | 0,879390777 | 0,100844237 | 2,207244567 | 0,579604095 |
| *mmu-miR-15b-5p* | 0,77775484 | **0,042961179** | 2,297048545 | 0,887207115 |
| *mmu-miR-181a-5p* | 0,169870757 | **0,013953499** | 0,550389287 | 0,339832988 |
| *mmu-miR-181a-1-3p* | 0,211514603 | **0,044237411** | 0,814324371 | 0,742811653 |
| *mmu-miR-181c-5p* | 0,652274407 | 0,42057463 | 2,800748862 | 0,923682802 |
| *mmu-miR-181d-5p* | 0,212755947 | **0,000602611** | 0,834629055 | 0,625426073 |
| *mmu-miR-191-5p* | **0,035494378** | 0,153775505 | 2,761688583 | 0,804763425 |
| *mmu-miR-193b-3p* | 0,409193162 | 0,067877601 | 2,847316718 | 0,773670175 |
| *mmu-miR-194-5p* | 0,479705765 | **0,016969877** | 1,390108524 | 0,86077921 |
| *mmu-miR-195a-5p* | 0,394693929 | 0,126473448 | 0,853153675 | 0,787983755 |
| *mmu-miR-19b-3p* | **0,039700343** | 0,277425246 | 2,016372865 | 0,812292235 |
| *mmu-miR-203-3p* | 0,53094531 | 0,119495407 | 1,200847117 | 0,702891344 |
| *mmu-miR-20a-5p* | **0,024458678** | 0,06073945 | 1,338914265 | 0,7823803 |
| *mmu-miR-20b-5p* | 0,797212733 | 0,098108795 | 1,31538624 | 0,733000042 |
| *mmu-miR-22-3p* | 0,795004122 | **0,00886307** | 2,41053699 | 0,637621526 |
| *mmu-miR-24-3p* | 0,82667534 | **0,035125855** | 0,996757146 | 0,716196411 |
| *mmu-miR-26b-5p* | 0,367896111 | **0,008187101** | 0,830605489 | 0,263395171 |
| *mmu-miR-27a-3p* | 0,830559927 | **0,005091937** | 1,449123986 | 0,71481915 |
| *mmu-miR-28a-5p* | 0,298240021 | 0,164731778 | 1,734594924 | 1,181571638 |
| *mmu-miR-298-5p* | 0,869851128 | **0,042178984** | 0,976148369 | 1,337260629 |
| *mmu-miR-29a-3p* | 0,772869223 | **0,01387036** | 0,523588658 | 0,170917158 |
| *mmu-miR-29b-3p* | 0,465798831 | 0,060754789 | 4,723532054 | 0,672916378 |
| *mmu-miR-29c-3p* | 0,46008146 | **0,023146758** | 0,782336845 | 3,56502723 |
| *mmu-miR-302a-5p* | 0,88764157 | 4,42543E-50 | 1,00870583 | 6,24963E+11 |
| *mmu-miR-302b-5p* | 0,237379949 | 0,116116524 | 0,2842556 | 812606,7018 |
| *mmu-miR-30a-5p* | 0,790479036 | **0,037042251** | 1,243080916 | 0,720100849 |
| *mmu-miR-30d-5p* | 0,382837492 | 0,121996456 | 1,200126113 | 0,690784163 |
| *mmu-miR-30e-5p* | 0,693390508 | **0,048200345** | 1,373611124 | 0,788119848 |
| *mmu-miR-320-3p* | 0,130251198 | 0,565928204 | 0,620204541 | 0,898436922 |
| *mmu-miR-328-3p* | 0,952354739 | 0,141287319 | 1,4954844 | 0,7999852 |
| *mmu-miR-33-5p* | 0,628418157 | 0,490645125 | 9,961011677 | 1,30664168 |
| *mmu-miR-337-3p* | 0,085462685 | 0,845920999 | 2,848309331 | 0,989788987 |
| *mmu-miR-338-3p* | 0,800901965 | **0,015677328** | 1,489224689 | 0,705953718 |
| *mmu-miR-339-5p* | 0,606425617 | 0,18675646 | 2,933336166 | 0,694920971 |
| *mmu-miR-342-3p* | **0,049067179** | 0,177716523 | 1,090664979 | 0,843794881 |
| *mmu-miR-346-5p* | 0,071156133 | 0,451455772 | 1,328065849 | 0,868192493 |
| *mmu-miR-34a-5p* | 0,096053944 | 0,587015107 | 2,311631867 | 0,940866943 |
| *mmu-miR-376b-3p* | 0,973154244 | 0,453989952 | 1,020424045 | 0,929779504 |
| *mmu-miR-381-3p* | 0,098907096 | 0,290688718 | 2,082155807 | 0,896618772 |
| *mmu-miR-409-3p* | 0,799097302 | 0,992927362 | 1,093785844 | 0,999892214 |
| *mmu-miR-431-5p* | 0,611766915 | **0,004668884** | 2,06389252 | 0,787709429 |
| *mmu-miR-433-3p* | 0,887685314 | 0,438597846 | 1,020848704 | 0,920215894 |
| *mmu-miR-455-5p* | 0,474207012 | 0,678457688 | 4,311397834 | 1,136911984 |
| *mmu-miR-484* | 0,728846018 | **0,033220662** | 1,428202676 | 0,579202639 |
| *mmu-miR-485-5p* | 0,840599734 | 0,18051833 | 0,991218673 | 1,233053166 |
| *mmu-miR-485-3p* | 0,703884115 | 0,242607964 | 1,218265374 | 0,84763453 |
| *mmu-miR-488-3p* | 0,854849545 | 0,181512201 | 2,283883195 | 0,852372625 |
| *mmu-miR-489-3p* | 0,808695504 | 0,065855614 | 1,092704787 | 0,467400778 |
| *mmu-miR-509-3p* | 0,373900966 | 0,999998999 | 94217146557 | 419836,1042 |
| *mmu-miR-598-3p* | 0,724370043 | 0,145269896 | 1,276096135 | 0,750863833 |
| *mmu-miR-652-3p* | 0,631684795 | 0,230772217 | 0,91858756 | 0,810508206 |
| *mmu-miR-7a-5p* | 0,918597941 | **0,030302065** | 1,039318548 | 0,611254285 |
| *mmu-miR-9-5p* | 0,241301747 | 0,191017753 | 0,822895244 | 0,860051537 |
| *mmu-miR-9-3p* | 0,085538707 | 0,184704023 | 1,426371553 | 0,874611424 |
| *mmu-miR-92a-3p* | 0,692347049 | **0,035449394** | 0,946633277 | 0,667891811 |
| *mmu-miR-93-5p* | **0,035285677** | 0,453574817 | 1,656645285 | 0,936756031 |
| *mmu-miR-98-5p* | **0,003475929** | 0,874931381 | 3,819656326 | 1,024587694 |
