# Supplementary material for: Temporal Integrative Analysis of mRNA and microRNAs Expression Profiles and Epigenetic Alterations in Female SAMP8, a Model of Age-Related Cognitive Decline
Source: Front Genet. 2018 Dec 11;9:596. doi: 10.3389/fgene.2018.00596 (PMC6297390; doi:10.3389/fgene.2018.00596)
Supplement: Supplementary file 5 [file Table_5.docx]

Supplementary material 5. Complete list of Go Biological processes associated with SAMP8 mice mRNA:miRNA predicted pairs for each condition (downregulated mRNA and upregulated miRNA, or upregulated mRNA and downregulated miRNA) and age (2- and 9-month-old).

| mirNAs up:mRNAs down: (GO Biological process) SAMP8 2 months | p-value | Z.score | Combined score |
| --- | --- | --- | --- |
| regulation of sulfur metabolic process (GO:0042762) | 0.0009151 | -2.81 | 5.99 |
| lipid translocation (GO:0034204) | 0.001015 | -2.74 | 5.86 |
| phospholipid translocation (GO:0045332) | 0.001015 | -2.73 | 5.83 |
| positive regulation of extrinsic apoptotic signaling pathway via death domain receptors (GO:1902043) | 0.0008200 | -2.69 | 5.75 |
| multi-organism reproductive process (GO:0044703) | 0.0003618 | -2.23 | 4.77 |
| cellular response to starvation (GO:0009267) | 0.0007969 | -2.22 | 4.74 |
| cellular response to nutrient levels (GO:0031669) | 0.001796 | -2.27 | 3.91 |
| response to starvation (GO:0042594) | 0.002172 | -2.24 | 3.72 |
| multi-multicellular organism process (GO:0044706) | 0.003131 | -2.18 | 3.53 |
| phagosome maturation (GO:0090382) | 0.004247 | -2.44 | 3.43 |
| response to nutrient levels (GO:0031667) | 0.005057 | -2.43 | 3.41 |
| humoral immune response (GO:0006959) | 0.1306 | -2.76 | 3.40 |
| response to extracellular stimulus (GO:0009991) | 0.006509 | -2.42 | 3.39 |
| cellular response to external stimulus (GO:0071496) | 0.009924 | -2.31 | 3.24 |
| cation homeostasis (GO:0055080) | 0.02435 | -2.30 | 3.23 |
| phospholipid transport (GO:0015914) | 0.006260 | -2.26 | 3.17 |
| anion transport (GO:0006820) | 0.02083 | -2.24 | 3.15 |
| iron ion transport (GO:0006826) | 0.008348 | -2.24 | 3.15 |
| steroid metabolic process (GO:0008202) | 0.01969 | -2.23 | 3.13 |
| cellular iron ion homeostasis (GO:0006879) | 0.01166 | -2.20 | 3.10 |
| positive regulation of extrinsic apoptotic signaling pathway (GO:2001238) | 0.008628 | -2.19 | 3.08 |
| iron ion homeostasis (GO:0055072) | 0.02093 | -2.19 | 3.08 |
| regulation of tissue remodeling (GO:0034103) | 0.008912 | -2.18 | 3.06 |
| organophosphate ester transport (GO:0015748) | 0.01298 | -2.11 | 2.96 |
| cellular metal ion homeostasis (GO:0006875) | 0.05477 | -2.09 | 2.94 |
| organic hydroxy compound transport (GO:0015850) | 0.02527 | -2.07 | 2.90 |
| transition metal ion transport (GO:0000041) | 0.02482 | -2.06 | 2.90 |
| glycoprotein metabolic process (GO:0009100) | 0.01437 | -2.06 | 2.89 |
| cholesterol metabolic process (GO:0008203) | 0.02757 | -2.05 | 2.88 |
| positive regulation of epithelial cell proliferation (GO:0050679) | 0.04696 | -2.05 | 2.88 |
| response to purine-containing compound (GO:0014074) | 0.04023 | -2.04 | 2.87 |
| cellular response to acid chemical (GO:0071229) | 0.04870 | -2.03 | 2.85 |
| transition metal ion homeostasis (GO:0055076) | 0.03392 | -2.02 | 2.84 |
| cellular cation homeostasis (GO:0030003) | 0.06547 | -2.00 | 2.81 |
| regulation of extrinsic apoptotic signaling pathway (GO:2001236) | 0.05713 | -2.00 | 2.81 |
| inorganic anion transmembrane transport (GO:0098661) | 0.02804 | -1.99 | 2.79 |
| regulation of cellular response to stress (GO:0080135) | 0.07158 | -1.99 | 2.79 |
| sterol metabolic process (GO:0016125) | 0.03341 | -1.98 | 2.79 |
| positive regulation of apoptotic signaling pathway (GO:2001235) | 0.06153 | -1.96 | 2.76 |
| cellular ion homeostasis (GO:0006873) | 0.07075 | -1.96 | 2.76 |
| regulation of canonical Wnt signaling pathway (GO:0060828) | 0.05165 | -1.93 | 2.71 |
| regulation of cellular ketone metabolic process (GO:0010565) | 0.06801 | -1.90 | 2.67 |
| monocarboxylic acid biosynthetic process (GO:0072330) | 0.06090 | -1.89 | 2.66 |
| inorganic anion transport (GO:0015698) | 0.03755 | -1.89 | 2.66 |
| sulfur compound biosynthetic process (GO:0044272) | 0.04753 | -1.89 | 2.65 |
| regulation of cellular response to growth factor stimulus (GO:0090287) | 0.06669 | -1.88 | 2.64 |
| response to defenses of other organism involved in symbiotic interaction (GO:0052173) | 0.03280 | -1.88 | 2.64 |
| xenobiotic metabolic process (GO:0006805) | 0.05407 | -1.87 | 2.63 |
| metal ion homeostasis (GO:0055065) | 0.07923 | -1.87 | 2.63 |
| response to host defenses (GO:0052200) | 0.03280 | -1.86 | 2.61 |
| negative regulation of cytokine production (GO:0001818) | 0.07267 | -1.85 | 2.60 |
| purinergic nucleotide receptor signaling pathway (GO:0035590) | 0.04653 | -1.85 | 2.60 |
| negative regulation of macrophage derived foam cell differentiation (GO:0010745) | 0.03280 | -1.84 | 2.58 |
| defense response to bacterium (GO:0042742) | 0.06867 | -1.81 | 2.55 |

| miRNAs down:mRNAs up: (GO Biological process) SAMP8 2 months | p-value | Z.score | Combined score |
| --- | --- | --- | --- |
| chondroitin sulfate catabolic process (GO:0030207) | 0.0001330 | -2.82 | 8.72 |
| regulation of catecholamine metabolic process (GO:0042069) | 0.0001589 | -2.75 | 8.51 |
| regulation of dopamine metabolic process (GO:0042053) | 0.0001589 | -2.73 | 8.46 |
| organ morphogenesis (GO:0009887) | 0.0001829 | -2.35 | 7.26 |
| regulation of angiogenesis (GO:0045765) | 0.0001244 | -2.30 | 7.12 |
| sulfur compound catabolic process (GO:0044273) | 0.0001762 | -2.30 | 7.11 |
| regulation of vasculature development (GO:1901342) | 0.0002201 | -2.30 | 7.01 |
| cellular response to organic cyclic compound (GO:0071407) | 0.0004199 | -2.49 | 6.88 |
| regulation of protein binding (GO:0043393) | 0.0001511 | -2.21 | 6.83 |
| regulation of cell size (GO:0008361) | 0.001184 | -2.48 | 6.56 |
| gland development (GO:0048732) | 0.0004207 | -2.32 | 6.43 |
| regulation of epithelial cell proliferation (GO:0050678) | 0.001050 | -2.41 | 6.38 |
| regulation of binding (GO:0051098) | 0.0003990 | -2.30 | 6.38 |
| regulation of cell adhesion (GO:0030155) | 0.001050 | -2.41 | 6.37 |
| pattern specification process (GO:0007389) | 0.0005759 | -2.35 | 6.33 |
| taxis (GO:0042330) | 0.001170 | -2.34 | 6.19 |
| chemotaxis (GO:0006935) | 0.001170 | -2.34 | 6.18 |
| cytokine-mediated signaling pathway (GO:0019221) | 0.001173 | -2.33 | 6.16 |
| positive regulation of angiogenesis (GO:0045766) | 0.0004334 | -2.19 | 6.06 |
| positive regulation of growth (GO:0045927) | 0.001113 | -2.28 | 6.02 |
| ameboidal-type cell migration (GO:0001667) | 0.001021 | -2.26 | 5.98 |
| cellular response to steroid hormone stimulus (GO:0071383) | 0.001021 | -2.24 | 5.94 |
| steroid hormone mediated signaling pathway (GO:0043401) | 0.0005471 | -2.19 | 5.92 |
| response to cocaine (GO:0042220) | 0.001394 | -2.35 | 5.92 |
| calcium ion homeostasis (GO:0055074) | 0.001473 | -2.33 | 5.82 |
| response to ammonium ion (GO:0060359) | 0.0008158 | -2.18 | 5.76 |
| patterning of blood vessels (GO:0001569) | 0.001507 | -2.30 | 5.75 |
| embryonic organ morphogenesis (GO:0048562) | 0.0009512 | -2.16 | 5.72 |
| mucopolysaccharide metabolic process (GO:1903510) | 0.001057 | -2.14 | 5.67 |
| glycosaminoglycan catabolic process (GO:0006027) | 0.0005810 | -2.09 | 5.65 |
| aminoglycan catabolic process (GO:0006026) | 0.0008606 | -2.07 | 5.49 |
| positive regulation of cellular component movement (GO:0051272) | 0.002259 | -2.42 | 5.34 |
| cellular response to cytokine stimulus (GO:0071345) | 0.002281 | -2.38 | 5.24 |
| divalent inorganic cation homeostasis (GO:0072507) | 0.002057 | -2.31 | 5.20 |
| cellular response to lipid (GO:0071396) | 0.003170 | -2.42 | 5.16 |
| branching morphogenesis of an epithelial tube (GO:0048754) | 0.001830 | -2.15 | 5.03 |
| regulation of anatomical structure size (GO:0090066) | 0.003043 | -2.35 | 5.00 |
| sulfur compound metabolic process (GO:0006790) | 0.003116 | -2.22 | 4.74 |
| regulation of cellular component size (GO:0032535) | 0.003538 | -2.27 | 4.71 |
| positive regulation of binding (GO:0051099) | 0.002728 | -2.17 | 4.69 |
| hormone-mediated signaling pathway (GO:0009755) | 0.002728 | -2.14 | 4.61 |
| morphogenesis of a branching epithelium (GO:0061138) | 0.003279 | -2.14 | 4.56 |
| skeletal system development (GO:0001501) | 0.003279 | -2.13 | 4.53 |
| glycosaminoglycan metabolic process (GO:0030203) | 0.003279 | -2.11 | 4.50 |
| enteric nervous system development (GO:0048484) | 0.003083 | -2.10 | 4.48 |
| phosphatidylinositol-3-phosphate biosynthetic process (GO:0036092) | 0.004554 | -2.21 | 4.42 |
| dopamine biosynthetic process (GO:0042416) | 0.002653 | -2.04 | 4.39 |
| cellular calcium ion homeostasis (GO:0006874) | 0.005326 | -2.23 | 4.37 |
| cytosolic calcium ion homeostasis (GO:0051480) | 0.004617 | -2.17 | 4.34 |
| positive regulation of steroid hormone secretion (GO:2000833) | 0.002653 | -2.01 | 4.34 |
| cellular response to hormone stimulus (GO:0032870) | 0.007099 | -2.30 | 4.34 |
| morphogenesis of a branching structure (GO:0001763) | 0.004003 | -2.15 | 4.34 |
| mammary gland epithelial cell differentiation (GO:0060644) | 0.005103 | -2.19 | 4.33 |
| cellular component disassembly (GO:0022411) | 0.005557 | -2.19 | 4.29 |
| DNA catabolic process (GO:0006308) | 0.003755 | -2.11 | 4.29 |
| cellular divalent inorganic cation homeostasis (GO:0072503) | 0.006348 | -2.19 | 4.29 |
| positive regulation of cell migration (GO:0030335) | 0.007384 | -2.27 | 4.29 |
| endothelial cell migration (GO:0043542) | 0.003923 | -2.12 | 4.28 |
| aminoglycan metabolic process (GO:0006022) | 0.004101 | -2.10 | 4.25 |
| response to steroid hormone (GO:0048545) | 0.007317 | -2.25 | 4.24 |
| epithelial cell maturation (GO:0002070) | 0.005103 | -2.13 | 4.21 |
| regulation of synaptic transmission, dopaminergic (GO:0032225) | 0.006288 | -2.15 | 4.20 |
| positive regulation of cell growth (GO:0030307) | 0.004426 | -2.10 | 4.19 |
| germ cell migration (GO:0008354) | 0.002653 | -1.94 | 4.18 |
| positive regulation of cell motility (GO:2000147) | 0.008270 | -2.25 | 4.16 |
| intracellular receptor signaling pathway (GO:0030522) | 0.005784 | -2.13 | 4.16 |
| regulation of chemotaxis (GO:0050920) | 0.006539 | -2.13 | 4.13 |
| columnar/cuboidal epithelial cell differentiation (GO:0002065) | 0.004135 | -2.04 | 4.12 |
| hindbrain development (GO:0030902) | 0.006288 | -2.09 | 4.09 |
| catecholamine biosynthetic process (GO:0042423) | 0.006288 | -2.07 | 4.05 |
| positive regulation of protein binding (GO:0032092) | 0.004582 | -2.02 | 4.04 |
| catechol-containing compound biosynthetic process (GO:0009713) | 0.006288 | -2.06 | 4.03 |
| response to alkaloid (GO:0043279) | 0.005018 | -2.04 | 4.02 |
| response to copper ion (GO:0046688) | 0.007586 | -2.12 | 4.00 |
| regulation of steroid hormone secretion (GO:2000831) | 0.005682 | -2.02 | 3.95 |
| cellular component disassembly involved in execution phase of apoptosis (GO:0006921) | 0.004582 | -1.96 | 3.92 |
| regulation of dopamine secretion (GO:0014059) | 0.006288 | -1.98 | 3.87 |
| electron transport chain (GO:0022900) | 0.005661 | -1.98 | 3.87 |
| chondroitin sulfate metabolic process (GO:0030204) | 0.006093 | -1.97 | 3.85 |
| apoptotic DNA fragmentation (GO:0006309) | 0.007586 | -2.04 | 3.85 |
| N-acetylglucosamine metabolic process (GO:0006044) | 0.005103 | -1.94 | 3.83 |
| regulation of receptor biosynthetic process (GO:0010869) | 0.007586 | -2.03 | 3.82 |
| glandular epithelial cell differentiation (GO:0002067) | 0.007586 | -2.01 | 3.79 |
| epithelial cell migration (GO:0010631) | 0.007869 | -1.98 | 3.71 |
| positive regulation of locomotion (GO:0040017) | 0.01074 | -2.23 | 3.68 |
| morphogenesis of an epithelium (GO:0002009) | 0.009519 | -2.12 | 3.65 |
| DNA catabolic process, endonucleolytic (GO:0000737) | 0.007869 | -1.93 | 3.61 |
| epithelial cell differentiation (GO:0030855) | 0.01058 | -2.16 | 3.57 |
| positive regulation of filopodium assembly (GO:0051491) | 0.01051 | -2.13 | 3.52 |
| dorsal/ventral pattern formation (GO:0009953) | 0.008191 | -1.84 | 3.40 |
| regulation of protein import into nucleus (GO:0042306) | 0.01174 | -2.09 | 3.39 |
| metal ion homeostasis (GO:0055065) | 0.01441 | -2.13 | 3.35 |
| adult behavior (GO:0030534) | 0.01120 | -2.03 | 3.35 |
| regulation of response to reactive oxygen species (GO:1901031) | 0.009739 | -1.96 | 3.35 |
| cell cycle arrest (GO:0007050) | 0.01094 | -2.03 | 3.34 |
| response to hypoxia (GO:0001666) | 0.01609 | -2.14 | 3.33 |
| response to decreased oxygen levels (GO:0036293) | 0.01713 | -2.12 | 3.30 |
| regulation of cell growth (GO:0001558) | 0.01388 | -2.07 | 3.25 |
| negative regulation of neuron death (GO:1901215) | 0.01527 | -2.06 | 3.24 |
| negative regulation of insulin receptor signaling pathway (GO:0046627) | 0.01213 | -1.99 | 3.19 |
| positive regulation of cytosolic calcium ion concentration (GO:0007204) | 0.01433 | -2.01 | 3.16 |
| regionalization (GO:0003002) | 0.01767 | -2.04 | 3.15 |
| regulation of extrinsic apoptotic signaling pathway (GO:2001236) | 0.01727 | -2.03 | 3.15 |
| positive regulation of epithelial cell migration (GO:0010634) | 0.01310 | -1.99 | 3.13 |
| dopamine metabolic process (GO:0042417) | 0.01385 | -1.99 | 3.13 |
| lung epithelial cell differentiation (GO:0060487) | 0.01475 | -1.99 | 3.12 |
| rhythmic process (GO:0048511) | 0.01907 | -2.07 | 3.12 |
| negative regulation of cell growth (GO:0030308) | 0.01373 | -1.97 | 3.10 |
| negative regulation of cellular response to insulin stimulus (GO:1900077) | 0.01385 | -1.97 | 3.0 |

| miRNAs up:mRNAs down: (GO Biological process) SAMP8 9 months | p-value | Z.score | Combined score |
| --- | --- | --- | --- |
| activation of immune response (GO:0002253) | 0.02822 | -3.10 | 5.12 |
| protein import into nucleus (GO:0006606) | 0.0007097 | -2.15 | 4.33 |
| single-organism nuclear import (GO:1902593) | 0.0007097 | -2.14 | 4.33 |
| nuclear import (GO:0051170) | 0.0008275 | -2.14 | 4.31 |
| regulation of MAP kinase activity (GO:0043405) | 0.004046 | -2.36 | 3.95 |
| protein import (GO:0017038) | 0.001899 | -2.20 | 3.91 |
| nucleotide-binding domain, leucine rich repeat containing receptor signaling pathway (GO:0035872) | 0.005781 | -2.33 | 3.90 |
| response to progesterone (GO:0032570) | 0.003109 | -2.32 | 3.89 |
| protein localization to nucleus (GO:0034504) | 0.001899 | -2.15 | 3.82 |
| regulation of transmembrane transport (GO:0034762) | 0.007564 | -2.31 | 3.80 |
| regulation of anatomical structure size (GO:0090066) | 0.01803 | -2.28 | 3.77 |
| pattern recognition receptor signaling pathway (GO:0002221) | 0.004857 | -2.25 | 3.76 |
| negative regulation of protein kinase activity (GO:0006469) | 0.01051 | -2.27 | 3.75 |
| innate immune response-activating signal transduction (GO:0002758) | 0.005045 | -2.24 | 3.74 |
| negative regulation of kinase activity (GO:0033673) | 0.01254 | -2.27 | 3.74 |
| positive regulation of innate immune response (GO:0045089) | 0.01065 | -2.26 | 3.72 |
| positive regulation of protein kinase activity (GO:0045860) | 0.02287 | -2.24 | 3.70 |
| regulation of protein serine/threonine kinase activity (GO:0071900) | 0.01697 | -2.24 | 3.70 |
| negative regulation of phosphate metabolic process (GO:0045936) | 0.02320 | -2.24 | 3.69 |
| response to endoplasmic reticulum stress (GO:0034976) | 0.005142 | -2.21 | 3.69 |
| negative regulation of phosphorus metabolic process (GO:0010563) | 0.02320 | -2.24 | 3.69 |
| receptor internalization (GO:0031623) | 0.005097 | -2.20 | 3.69 |
| toll-like receptor signaling pathway (GO:0002224) | 0.003203 | -2.20 | 3.68 |
| negative regulation of transporter activity (GO:0032410) | 0.005097 | -2.19 | 3.67 |
| negative regulation of protein phosphorylation (GO:0001933) | 0.02420 | -2.20 | 3.63 |
| positive regulation of kinase activity (GO:0033674) | 0.02695 | -2.19 | 3.62 |
| lipid transport (GO:0006869) | 0.01111 | -2.19 | 3.62 |
| MAPK cascade (GO:0000165) | 0.007911 | -2.19 | 3.61 |
| regulation of innate immune response (GO:0045088) | 0.02279 | -2.17 | 3.58 |
| signal transduction by phosphorylation (GO:0023014) | 0.01254 | -2.17 | 3.58 |
| response to virus (GO:0009615) | 0.02188 | -2.16 | 3.57 |
| positive regulation of defense response (GO:0031349) | 0.02715 | -2.16 | 3.56 |
| positive regulation of extrinsic apoptotic signaling pathway (GO:2001238) | 0.008628 | -2.16 | 3.56 |
| regulation of blood vessel size (GO:0050880) | 0.01040 | -2.14 | 3.54 |
| nucleocytoplasmic transport (GO:0006913) | 0.01287 | -2.14 | 3.53 |
| negative regulation of transferase activity (GO:0051348) | 0.03029 | -2.14 | 3.53 |
| angiogenesis (GO:0001525) | 0.01885 | -2.14 | 3.53 |
| nuclear transport (GO:0051169) | 0.01372 | -2.13 | 3.52 |
| cellular response to tumor necrosis factor (GO:0071356) | 0.01969 | -2.13 | 3.51 |
| regulation of tube size (GO:0035150) | 0.01070 | -2.12 | 3.50 |
| negative regulation of transmembrane transport (GO:0034763) | 0.007011 | -2.11 | 3.48 |
| body fluid secretion (GO:0007589) | 0.01298 | -2.11 | 3.47 |
| secretion by tissue (GO:0032941) | 0.009790 | -2.10 | 3.47 |
| mesenchyme development (GO:0060485) | 0.008072 | -2.10 | 3.47 |
| activation of signaling protein activity involved in unfolded protein response (GO:0006987) | 0.01070 | -2.10 | 3.47 |
| vascular process in circulatory system (GO:0003018) | 0.01849 | -2.10 | 3.46 |
| positive regulation of nuclease activity (GO:0032075) | 0.01133 | -2.09 | 3.44 |
| regulation of nuclease activity (GO:0032069) | 0.01332 | -2.08 | 3.43 |
| response to heat (GO:0009408) | 0.01264 | -2.08 | 3.43 |
| positive regulation of Wnt signaling pathway (GO:0030177) | 0.01849 | -2.07 | 3.42 |
| MyD88-dependent toll-like receptor signaling pathway (GO:0002755) | 0.01694 | -2.07 | 3.41 |
| negative regulation of phosphorylation (GO:0042326) | 0.04240 | -2.04 | 3.37 |
| negative regulation of MAP kinase activity (GO:0043407) | 0.01401 | -2.04 | 3.36 |
| toll-like receptor 9 signaling pathway (GO:0034162) | 0.01367 | -2.03 | 3.35 |
| endoplasmic reticulum unfolded protein response (GO:0030968) | 0.01732 | -2.02 | 3.34 |
| cellular response to topologically incorrect protein (GO:0035967) | 0.02093 | -2.02 | 3.33 |
| response to calcium ion (GO:0051592) | 0.02438 | -1.97 | 3.25 |
| regulation of ion transmembrane transport (GO:0034765) | 0.03894 | -1.96 | 3.23 |
| membrane depolarization (GO:0051899) | 0.02710 | -1.95 | 3.2 |

| miRNAs down:mRNAs up: (GO Biological process) SAMP8 9 months | p-value | Z.score | Combined score |
| --- | --- | --- | --- |
| androgen receptor signaling pathway (GO:0030521) | 0.01200 | -2.11 | 7.07 |
| regulation of receptor activity (GO:0010469) | 0.02445 | -2.14 | 6.24 |
| steroid biosynthetic process (GO:0006694) | 0.03021 | -2.12 | 6.14 |
| hemopoiesis (GO:0030097) | 0.02665 | -2.11 | 6.13 |
| embryonic organ development (GO:0048568) | 0.03322 | -2.13 | 6.08 |
| cytokinesis (GO:0000910) | 0.02224 | -2.04 | 5.97 |
| peptidyl-lysine acetylation (GO:0018394) | 0.03021 | -2.06 | 5.97 |
| internal protein amino acid acetylation (GO:0006475) | 0.03103 | -2.05 | 5.95 |
| internal peptidyl-lysine acetylation (GO:0018393) | 0.02966 | -2.05 | 5.94 |
| anterior/posterior pattern specification (GO:0009952) | 0.04167 | -2.18 | 5.91 |
| protein acetylation (GO:0006473) | 0.03404 | -2.06 | 5.85 |
| peptidyl-lysine modification (GO:0018205) | 0.04248 | -2.12 | 5.76 |
| protein acylation (GO:0043543) | 0.04276 | -2.09 | 5.67 |
| Intracellular receptor signaling pathway (GO:0030522) | 0.05041 | -2.15 | 5.61 |
