# Supplementary material for: Temporal Integrative Analysis of mRNA and microRNAs Expression Profiles and Epigenetic Alterations in Female SAMP8, a Model of Age-Related Cognitive Decline
Source: Front Genet. 2018 Dec 11;9:596. doi: 10.3389/fgene.2018.00596 (PMC6297390; doi:10.3389/fgene.2018.00596)
Supplement: Supplementary file 6 [file Table_6.docx]

**Supplementary material 6.** Venn diagram highlighting hippocampal genes concurrently altered in our study with females SAMP8 at 2 and 9 months of age in comparison with two studies (Zhen et al., 2008 and Cheng et al., 2013) with males SAMP8 at 2, 6 and 12 months of age.
